# Supplementary material for: Genomes of trombidid mites reveal novel predicted allergens and laterally transferred genes associated with secondary metabolism
Source: Gigascience. 2018 Nov 15;7(12):giy127. doi: 10.1093/gigascience/giy127 (PMC6275457; doi:10.1093/gigascience/giy127)
Supplement: GIGA-D-18-00044_Revision_3.pdf [file giy127_giga-d-18-00044_revision_3.pdf]

GigaScience

Genomes of trombidid mites reveal novel predicted allergens and laterally-transferred genes associated with secondary metabolism

--Manuscript Draft--

**Manuscript Number:**	GIGA-D-18-00044R3
**Full Title:**	Genomes of trombidid mites reveal novel predicted allergens and laterally-transferred genes associated with secondary metabolism
**Article Type:**	Research
**Funding Information:**	Bayer — Not applicable
**Abstract:**	Background<br>Trombidid mites have a unique lifecycle in which only the larval stage is ectoparasitic. In the superfamily Trombiculoidea ('chiggers'), the larvae feed preferentially on vertebrates, including humans. Species in the genus Leptotrombidium are vectors of a potentially fatal bacterial infection, scrub typhus, which affects 1 million people annually. Moreover, chiggers can cause pruritic dermatitis (trombiculiasis) in humans and domesticated animals. In the Trombidioidea (velvet mites), the larvae feed on other arthropods and are potential biological control agents for agricultural pests. Here, we present the first trombidid mites genomes, obtained both for a chigger, Leptotrombidium deliense, and for a velvet mite, Dinothrombium tinctorium.<br>Results<br>Sequencing was performed using Illumina technology. A 180 Mb draft assembly for D. tinctorium was generated from two paired-end and one mate-pair library using a single adult specimen. For L. deliense, a lower-coverage draft assembly (117 Mb) was obtained using pooled, engorged larvae with a single paired-end library. Remarkably, both genomes exhibited evidence of ancient lateral gene transfer from soil-derived bacteria or fungi. The transferred genes confer functions that are rare in animals, including terpene and carotenoid synthesis. Thirty-seven allergenic protein families were predicted in the L. deliense genome, of which nine were unique. Preliminary proteomic analyses identified several of these putative allergens in larvae.<br>Conclusions<br>Trombidid mite genomes appear to be more dynamic than those of other acariform mites. A priority for future research is to determine the biological function of terpene synthesis in this taxon and its potential for exploitation in disease control.
**Corresponding Author:**	Benjamin L Makepeace, MSc PhD<br>University of Liverpool<br>Liverpool, Merseyside UNITED KINGDOM
**Corresponding Author Secondary Information:**	
**Corresponding Author's Institution:**	University of Liverpool
**Corresponding Author's Secondary Institution:**	
**First Author:**	Xiaofeng Dong
**First Author Secondary Information:**	
**Order of Authors:**	Xiaofeng Dong<br>Kittipong Chaisiri<br>Dong Xia<br>Stuart D Armstrong<br>Yongxiang Fang<br>Martin J Donnelly<br>Tatsuhiko Kadowaki

Powered by Editorial Manager® and ProduXion Manager® from Aries Systems Corporation

	John W McGarry
	Alistair C Darby
	Benjamin L Makepeace
**Order of Authors Secondary Information:**	
**Response to Reviewers:**	Dear Dr Zauner,<br><br>Following our e-mail discussion, I have removed the "data not shown" statement pertaining to the Bayesian tree method. I have also uploaded new figures at higher resolution.<br><br>With thanks for your assistance,<br>Ben Makepeace
**Additional Information:**	
**Question**	**Response**
Are you submitting this manuscript to a special series or article collection?	No
**Experimental design and statistics**<br><br>Full details of the experimental design and statistical methods used should be given in the Methods section, as detailed in our Minimum Standards Reporting Checklist. Information essential to interpreting the data presented should be made available in the figure legends.<br><br>Have you included all the information requested in your manuscript?	Yes
**Resources**<br><br>A description of all resources used, including antibodies, cell lines, animals and software tools, with enough information to allow them to be uniquely identified, should be included in the Methods section. Authors are strongly encouraged to cite Research Resource Identifiers (RRIDs) for antibodies, model organisms and tools, where possible.<br><br>Have you included the information requested as detailed in our Minimum Standards Reporting Checklist?	Yes
**Availability of data and materials**	Yes

All datasets and code on which the conclusions of the paper rely must be either included in your submission or deposited in publicly available repositories (where available and ethically appropriate), referencing such data using a unique identifier in the references and in the “Availability of Data and Materials” section of your manuscript.<br><br>Have you have met the above requirement as detailed in our Minimum Standards Reporting Checklist?	

Powered by Editorial Manager® and ProduXion Manager® from Aries Systems Corporation

Manuscript

Click here to view linked References

Click here to access/download;Manuscript;Dong et al 2018_final-submission_flat.docx

Genomes of trombidid mites reveal novel predicted allergens and laterally-transferred genes associated with secondary metabolism

Xiaofeng Dong[1,2,3,4], Kittipong Chaisiri[4,5], Dong Xia[4,6], Stuart D. Armstrong[4], Yongxiang Fang[1], Martin J. Donnelly[7], Tatsuhiko Kadowaki[2], John W. McGarry[8], Alistair C. Darby[1†], Benjamin L. Makepeace[4†*]

[1]Institute of Integrative Biology, University of Liverpool, Liverpool L69 7ZB, United Kingdom, [2]Department of Biological Sciences, Xi'an Jiaotong-Liverpool University, Suzhou 215123, China, [3]School of Life Sciences, Jiangsu Normal University, Xuzhou 221116, China, [4]Institute of Infection & Global Health, University of Liverpool, L3 5RF, United Kingdom, [5]Faculty of Tropical Medicine, Mahidol University, Ratchathewi Bangkok 10400, Thailand, [6]The Royal Veterinary College, London NW1 0TU, United Kingdom, [7]Department of Vector Biology, Liverpool School of Tropical Medicine, Liverpool L3 5QA, United Kingdom, [8]Institute of Veterinary Science, University of Liverpool, Liverpool L3 5RP, United Kingdom.

†Contributed equally.

*Correspondence address: Ben Makepeace, Department of Infection Biology, Institute of infection & Global Health, Liverpool Science Park IC2, 146 Brownlow Hill, Liverpool L3 5RF, United Kingdom. Tel: +44 151-7941586; Fax: +44 151 7950236; E-mail: blm1@liv.ac.uk

Abstract

Background

Trombidid mites have a unique lifecycle in which only the larval stage is ectoparasitic. In the superfamily Trombiculoidea ("chiggers"), the larvae feed preferentially on vertebrates, including humans. Species in the genus *Leptotrombidium* are vectors of a potentially fatal bacterial infection, scrub typhus, which affects 1 million people annually. Moreover, chiggers can cause pruritic dermatitis (trombiculiasis) in humans and domesticated animals. In the Trombidioidea (velvet mites), the larvae feed on other arthropods and are potential biological control agents for agricultural pests. Here, we present the first trombidid mites genomes, obtained both for a chigger, *Leptotrombidium deliense*, and for a velvet mite, *Dinothrombium tinctorium*.

Results

Sequencing was performed using Illumina technology. A 180 Mb draft assembly for *D. tinctorium* was generated from two paired-end and one mate-pair library using a single adult specimen. For *L. deliense,* a lower-coverage draft assembly (117 Mb) was obtained using pooled, engorged larvae with a single paired-end library. Remarkably, both genomes exhibited evidence of ancient lateral gene transfer from soil-derived bacteria or fungi. The transferred genes confer functions that are rare in animals, including terpene and carotenoid synthesis. Thirty-seven allergenic protein families were predicted in the *L. deliense* genome, of which nine were unique. Preliminary proteomic analyses identified several of these putative allergens in larvae.

Conclusions

Trombidid mite genomes appear to be more dynamic than those of other acariform mites. A priority for future research is to determine the biological function of terpene synthesis in this taxon and its potential for exploitation in disease control.

Keywords

Chigger, trombiculid, scrub typhus, terpenes, isoprenoids, horizontal transfer, *Leptotrombidium*, *Dinothrombium*, *Tetranychus,* Trombidiformes.

Background

The Acari (mites and ticks) are the most speciose group within the subphylum Chelicerata, with approximately 55,000 described species in both terrestrial and aquatic habitats, and an estimated total diversity of up to 1 million species [1]. This assemblage is paraphyletic and is composed of two major divisions, the Parasitiformes and the Acariformes, which both contain species of medical, veterinary and agricultural importance. For example, the Parasitiformes harbour predatory mites used in the control of agricultural pests (*e.g.*, *Metaseiulus occidentalis*); ectoparasites of honey bees (*Varroa destructor* and *Tropilaelaps mercedesae*) that transmit pathogenic viruses; and most famously, the ticks (Ixodida). The Acariformes include major ectoparasites and sources of allergens for humans and other animals, such as the scabies mite (*Sarcoptes scabiei*) and the dust mites (*Dermatophagoides* spp. and *Euroglyphus maynei*).

The role of lateral gene transfer (LGT) in the evolution of animals has remained controversial since the first metazoan genomes were sequenced. Despite the explosion of new genomic resources across a wider variety of metazoan phyla in recent years, some claims of large-scale LGT in animal genomes have been shown to be the result of flawed data-analysis methods that failed to exclude sequences from bacterial contaminants [2-5]. Attempts to conduct meta-analyses across diverse metazoan genomes using consistent criteria have also been criticized for reliance on unsound assumptions [6, 7]. However, it is irrefutable that several metazoan taxa are reliant on functions obtained via LGT for essential physiological processes, including digestion of complex and/or toxic materials (especially in the case of herbivores and plant parasites) and the avoidance of host defences [8-11]. One group of acariform mites, the spider mites (superfamily Tetranychoidea: order Trombidiformes; Fig. 1), are major pests of various crops and rely on well-characterised lateral gene transfers of β-cyanoalanine synthase (from bacteria) and carotenoid biosynthesis enzymes (from fungi) to detoxify hydrogen cyanide in their diet [12] and to control diapause [13], respectively. Moreover, a recent in-depth analysis of LGT in the two-spotted spider mite (*Tetranychus urticae*) revealed *in silico* evidence that

this species can synthesise pantothenic acid using genes of bacterial origin incorporated into its genome [14]. Currently, it is unknown whether LGT is a key feature of the evolution of the spider mites only, or is more widespread among the Trombidiformes.

In addition to the Tetranychoidea, the Trombidiformes contain two other superfamilies of economic or clinical importance, the Trombidioidea (the velvet mites) and Trombiculoidea (the "chiggers" and related groups) [15]. These two taxa, known collectively as trombidid mites (the Trombidiae; Fig. 1), have a unique natural history among arthropods in that only the larval stage is ectoparasitic, whereas the nymphs and adults are predators of other arthropods (Fig. 2). However, the Trombidioidea and Trombiculoidea differ in their host preferences. Larvae of the Trombidioidea are exclusively parasites of other arthropods and some species feed on insects of medical, veterinary and agricultural importance, including mosquitoes [16], the New World screwworm fly [17], and aphids [18] (Fig. 2). On dipteran hosts, heavy infestations can reduce flight ability, while certain aphid species can be killed in a few days by as few as two feeding larvae [19, 20].

The more heavily-studied larval stages of the Trombiculoidea (commonly referred to as chiggers or berry bugs) primarily feed on terrestrial vertebrates, including humans [21], although some little-known taxa in this superfamily are ectoparasites of invertebrate hosts in common with the Trombidioidea [22-24] (Fig. 2). Importantly, the only major mite-transmitted disease of humans, scrub typhus or tsutsugamushi disease, is vectored by chiggers in the genus *Leptotrombidium* [25], while other chigger genera have only been implicated epidemiologically as locally-important vectors [26]. Scrub typhus is a severe febrile illness caused by infection with an obligate intracellular bacterium (*Orientia* spp.) in the order *Rickettsiales* and features an epidemiological cycle that includes wild small mammals, which are the primary hosts for many chigger species [27]. This disease has a fatality rate of 6% if not treated promptly with antibiotics [28] and has increased in incidence globally in recent years, with the annual minimum incidence reaching >17/100,000 in South Korea and Thailand in 2012 – 2013 [29]. In the so-called "tsutsugamushi triangle" within the Asia-Pacific region, scrub typhus has

a median seroprevalence of 22.2% [29]; but endemic scrub typhus has emerged in several other parts of the world within the past decade, including South America [30], the Middle East [31], and possibly sub-Saharan Africa [32]. Chiggers have also been implicated in the transmission of hantaviruses [33], *Bartonella* spp. [34] and *Rickettsia* spp. [35]. Furthermore, chiggers have direct impacts worldwide by causing trombiculiasis, which is a highly pruritic dermatitis that can afflict humans, companion animals and domestic ruminants, potentially leading to severe hypersensitivity [36-39].

A remarkable second unique feature of trombidid mites is that the larvae induce the formation of a feeding tube or “stylostome” at the attachment site that is extraneous to the larval mouthparts [40]. These larvae are not blood feeders, but ingest tissue exudates (in the case of vertebrate hosts) or arthropod haemolymph [41]. The life history of trombidid nymphs and adults has been poorly studied. However, in the Trombiculoidea, the eggs of Collembola (springtails) and other arthropods are an important part of the diet [42] (Fig. 2). Arthropod eggs may also serve as food items for adults and nymphs in the Trombidioidea [43], although some species have potential roles in biological control, as they feed on pest arthropods such as spider mites, scale insects, aphids and termites [20, 44-46].

To date, research on trombidid mites has suffered from a dearth of molecular data that could facilitate studies on speciation; population structure; host-vector and vector-pathogen interactions; and life-history evolution in this group. To address this deficit, here we present a comparative analysis of the genomes of the chigger *Leptotrombidium deliense* (the primary scrub typhus vector in South-East Asia [47]) and the giant red velvet mite, *Dinothrombium tinctorium* (the world’s largest acarine species [48]). We show that these trombidid mites form a distinct branch of the Trombidiformes that exhibit two classes of LGT for secondary metabolism: the previously identified carotenoid biosynthesis enzymes of fungal origin, and much larger terpene synthase gene families, which probably derive from soil-associated bacteria. We also identify unique clusters of predicted allergens in *L. deliense* that may contribute to the symptoms of trombiculiasis in humans and domestic animals.

Data description

Since the specimens were highly disparate in physical size (adult *D. tinctorium* can reach ~16 mm in length, whereas larval *L. deliense* do not normally exceed 250 µm, even when engorged), a tailored approach to sequencing was necessary in each case. For the velvet mite, DNA from a single adult was used to generate two Illumina TruSeq libraries (insert sizes, 350 bp and 550 bp) and one Nextera mate-pair library (insert size, 3 Kb). The TruSeq libraries were barcoded, indexed and paired-end (PE)-sequenced (2 × 100 bp) on one lane, and the Nextera library was PE-sequenced (2 × 250 bp) on an additional lane, both on the Illumina MiSeq platform. For *L. deliense*, DNA from a pool of engorged larvae (obtained from Berdmore's ground squirrels in Thailand) was used to produce one NEB Next Ultra DNA library (insert size, 550 bp) and PE-sequenced (2 × 150 bp) on the Illumina MiSeq.

The total number of trimmed reads generated was ~362 million for *D. tinctorium* and ~38 million for *L. deliense.* For the former, PE reads were assembled using Abyss (v. 1.5.2) [49, 50]. For the *L. deliense* data, a preliminary assembly to contig level was performed using Velvet (v. 1.2.07) [51]. Reads derived from mammalian host genomic DNA were removed from the preliminary genome assembly using blobtools (v0.9.19) , which generates a GC-coverage plot (proportion of GC bases and node coverage) [52] (Fig. 3). The *L. deliense* genome was then reassembled using SPAdes assembler (v. 3.7.1) [53] with default settings. For gene calling, the MAKER pipeline [54] was used to integrate *ab initio* gene predictions from Augustus (v. 3.2.2) [55], SNAP (v. 2013-11-29) [56] and GeneMark (v. 2.3e) [57] with evidence-based gene models. The "Methods" section provides more details on how downstream genome analyses were performed.

Similarly to the genome sequencing strategy, preliminary proteomic analyses of the trombidid mites was customised to the sample types available. A single adult *D. tinctorium* was subjected to protein extraction in SDS buffer and tryptic digestion using the filter-aided sample preparation method [58]. The digested sample was split into eight fractions using a High pH Reversed-Phase Peptide Fractionation Kit (Pierce) prior to nanoLC MS ESI MS/MS analysis on a Q-Exactive mass spectrometer

(Thermo Fisher Scientific). A total of 137,638 spectra were generated across the eight fractions. For *L. deliense*, a soluble protein extract was obtained from a small pool of ethanol-fixed engorged larvae (*n* = 10) collected from several species of wild rodents in Thailand [27]. Following tryptic digestion, downstream analyses proceeded as for *D. tinctorium*, producing 18,059 spectra. The "Methods" section provides details on how MS spectra searches and Pfam enrichment analyses were performed.

Analyses

Genome statistics and phylogenomics

Assembled genome sizes were 180.41 Mb for *D. tinctorium* and 117.33 Mb for *L. deliense* (supplemental table S1), whereas *k*-mer analysis placed the genome size estimates much closer together but was of a similar scale to the assemblies (143.52 – 147.09 and 158.31 – 160.95 Mb, respectively; supplemental fig. S1). The estimate for *L. deliense* was slightly smaller than those determined for *Leptotrombidium pallidum* and *Leptotrombidium scutellare* using DNA from laboratory-reared adult specimens, which were 191 ± 7 Mb and 262 ± 13 Mb (by qPCR), or 175 Mb and 286 Mb (by *k*-mer analysis), respectively [59]. The repeat content of the new genomes presented a significant challenge, with unclassified repeats alone accounting for 19 - 23% of the total size; approximately double the proportion of the *S. scabiei* [60] and *T. urticae* [61] genomes (supplemental table S2). As expected from sequencing a pool of *L. deliense* larvae compared with a single adult of *D. tinctorium*, and the corresponding DNA library strategies employed, the chigger genome was considerably less contiguous than that of the velvet mite. Nevertheless, the estimated completeness of the predicted gene set for *L. deliense* (67% complete, 7.1% duplicated, 12% fragmented, 19% missing) based on the Benchmarking Universal Single-Copy Orthologues (BUSCO, RRID:SCR_015008) criteria [62] compared favourably with that of other arachnid genomes (supplemental table S1).

Notably, the *D. tinctorium* genome contained the greatest number of protein-coding genes from the Acariformes sequenced to date and a very rate of duplicated conserved genes (34%) according to BUSCO (BUSCO, RRID:SCR_015008) analysis (supplemental table S1). To address possible assembly artefacts caused by high levels of heterozygosity leading to uncollapsed haplotypes, we ran the Redundans pipeline [63] on the genomic scaffolds for *D. tinctorium*, which reduced the total scaffold number by 45% and the gene content by 36% (supplemental table S3). Accordingly, the BUSCO duplicated gene proportion was decreased from 34% to 12% without a detrimental impact on the number of complete conserved genes (supplemental table S3). However, this result seemed to

contradict the *k*-mer analysis, which had not identified high levels of heterozygosity in this genome (supplemental figure S1b). Moreover, reciprocal BLAST analysis of the genomic scaffolds revealed that while the duplicated genes were identical at the nucleotide level, they were always located on scaffolds with distinct sequence context outside the gene boundaries, even if this sometimes amounted to only individual SNPs or indels (supplemental table S4). As we had achieved high genome coverage (175 ×) and a genuine large-scale chromosomal duplication event was deemed plausible (see Discussion), the Abyss assembly was used for downstream analysis without removal of "redundant" scaffolds.

Maximum likelihood and Bayesian phylogenomic analyses based on 476 one-to-one orthologous genes exhibited complete concordance, placing *L. deliense* and *D. tinctorium* together as sister taxa, and *T. urticae* as their closest relative amongst sequenced species (Fig. 1, Fig. 4). Our divergence time estimates accord closely with those previously published for arachnids [64], with the Parasitiformes and Acariformes separating approximately 430 million years ago (MYA) (Fig. 4). We estimate that the trombidid mites *sensu stricto* (velvet mites and chiggers) diverged from the phytophagous Tetranychoidea 265 MYA, and finally the Trombidioidea and Trombiculoidea last shared a common ancestor approximately 133 MYA (Fig. 4).

Gene family expansions

When gene families were compared among the Acariformes and reference invertebrate genomes, the *D. tinctorium* genome was shown to contain a substantially greater number of unique paralogous groups than the other sequenced acariform mites, while only 56 gene clusters were shared among all Acariformes (supplemental fig S2). Moreover, the *D. tinctorium* genome displayed a greater number of multi-copy ("N:N:N") and patchy orthologues when analysed alongside the published arachnid genomes (Fig. 4). The gene family expansion in *D. tinctorium* also dwarfed that seen among other members of the Arachnida, including *L. deliense* (supplemental fig. S3).

Relative to other acariform mites, *D. tinctorium* exhibited a significant expansion of 56 gene families, including a large family (ORTHOMCL104) of uncharacterised proteins containing 47 members in this species but no representatives in the other acariform genomes (supplemental table S5b). Examination of conserved domains in this gene family revealed a major facilitator superfamily domain with some weak but significant similarity by BLASTP (~25% amino-acid identity, >95% coverage) to feline leukemia virus subgroup C receptor-related protein (FLVCR)-1 from various Metazoa. Two other orthologous clusters displayed an identity of up to 30% with FLVCR2 and showed a statistically significant expansion in this species, containing 27 (ORTHOMCL316) and 21 (ORTHOMCL484) members, compared with only one and three members in *L. deliense*, respectively (supplemental table S5b).

Overrepresented protein families detected by LC-MS/MS

From the extremely small sample of engorged *L. deliense*, 522 mite proteins were identified by shotgun LC-MS/MS with at least one unique peptide against a background of 292 proteins of putative host origin (supplemental table S6b). Of the *L. deliense* proteins, 290 were considered as high-confidence identifications (≥2 unique peptides; supplemental table S6a) and were subjected to Pfam domain enrichment analysis. The most overrepresented protein domains were derived from ATP synthase (PF00006 and PF02874) and both muscle and non-muscle isoforms of myosin or paramyosin (PF01576), although key enzymes of the citric acid cycle (ATP citrate synthase and succinate-CoA ligase, PF00549) were also well represented (Fig. 5). Several other proteins with probable origins from muscle tissue contained calponin homology (CH) domains (PF00307) and/or spectrin repeat domains (PF00435), including muscle protein 20 [65], myophilin, actinin, and spectrin subunits (Fig. 5). Myophilin is an invertebrate-specific protein that has previously been characterised as an immunogenic muscle component from parasitic platyhelminths [66], suggesting that it may contribute to the inflammatory response during trombiculiasis.

In a preliminary proteomic analysis of *D. tinctorium*, processing of a single adult led to the robust identification (≥2 unique peptides) of 1,636 proteins, as the large size of the specimen was conducive

to peptide fractionation prior to LC-MS/MS (supplemental table S7). Laminin B (PF00052) was the most enriched domain (Fig. 5), which was present in several isoforms of perlecan (the basement membrane-specific heparan sulfate proteoglycan core protein). Perlecan is highly expressed during embryonic development in *Drosophila* [67], suggesting that this mite specimen may have contained fertilised eggs. Indeed, this specimen was undoubtedly a female, as it contained vitellogenins (yolk proteins) (supplemental table S7). Lyase-1 domains (PF00206) were also highly enriched (Fig. 5) and were found in mitochondrial fumarate hydratase (an enzyme of the citric acid cycle) and in adenylosuccinate lyase of the purine nucleotide cycle. The peptidase M20 (PF01546) and M20 dimer domains (PF07687) were both present in cytosol non-specific dipeptidases, which have a key role in protein digestion in the midgut [68], and in N-fatty-acyl-amino acid synthase-hydrolases, which regulate thermogenesis via uncoupled respiration [69]. Another group of proteins with a putative role in thermoregulation were the α-crystallin-like small heat shock proteins (PF00525); in ticks, these are highly immunogenic proteins expressed in tick salivary glands and exhibit thermoprotective activity [70]. Finally, the inhibitor I29 domain (PF08246) represented digestive cysteine proteinases [71] and cathepsin L-like proteases (Fig. 5), which are highly expressed in feeding stages of *T. urticae* [72] and are a major protein component of spider mite faeces [73].

Lateral gene transfers and mobile elements

To determine the origin of the bright colouration of the trombidid mites, we searched both genomes for the fused carotenoid synthases-cyclases that were reported to have been laterally transferred into the *T. urticae* genome from zycomycete fungi, perhaps via aphids [61]. In common with *T. urtic*ae, two of these carotenoid synthases-cyclases were observed in the *L. deliense* genome, while *D. tinctorium* harboured 12 copies (supplemental fig. S4). However, the single largest gene family expansion observed in the *L. deliense* genome was within an orthologous cluster annotated as “pentalenene synthase”, which contained 39 members (supplemental table S5a). This cluster (ORTHOMCL65) also contained 21 genes in the *D. tinctorium* genome, but lacked orthologues in the genomes of other

arachnids. A second orthologous cluster (ORTHOMCL1284) of terpene synthases contained 17 members and was unique to *L. deliense* (supplemental table S5a).

The capacity to generate terpenoids (also known as isoprenoids) *de novo* in metazoans is extremely unusual. While some millipedes (for instance, the Japanese species *Niponia nodulosa*) are known to produce terpenes such as geosmin and 2-methylisoborneol in defensive secretions, these secondary metabolites are assumed to be derived from microbial symbionts [74]. The absence of terpene synthases in the fully sequenced diplopod genome from the rusty millipede, *Trigoniulus corallinus*, certainly supports this interpretation [75]. Dust mites also produce a monoterpene, neryl formate, which has been demonstrated to act as an aggregation pheromone [76]. However, BLAST analysis of the *Dermatophagoides farinae* genome assembly [77] using the trombidid terpene synthases failed to identify significant homologues, suggesting that dust mites rely on microbial symbionts for terpene production, or that terpene synthases in mite genomes are evolving too rapidly to be identified by homology searches. To the best of our knowledge, the only animals known to harbour terpene synthase genes in their nuclear genomes are a very restricted number of beetle species [predominantly flea beetles of the subfamily Galerucinae, which produce (6*R*,7*S*)-himachala-9,11-diene as a male aggregation pheromone [78]] and the collembolan *Folsomia candida*, in which the metabolites produced and their function are unknown [79]. Whilst terpene synthases are widespread in plants, fungi and bacteria, the terpene synthases from flea beetles do not resemble those from non-metazoan taxa and appear to have evolved from arthropod *trans*-isoprenyl diphosphate synthases [78]. Moreover, the terpene synthases of *F. candida* are more similar to those from the flea beetles than they are to the non-metazoan enzymes [79].

We generated phylogenies for the trombidid terpene synthases, which clearly showed close affinities to bacterial and fungal homologues and not to those from other arthropods (Fig. 6, Fig. 7). For ORTHOMCL65, the *L. deliense* and *D. tinctorium* enzymes formed distinct groups, and the closest homologues from other taxa included a monoterpene synthase from *Micromonospora* spp. (phylum

Actinobacteria), a genus that is known to synthesise 2-methylenebornane [80], as well as related genes from agaricomycete fungi (Fig. 6). In the case of the *L. deliense*-specific ORTHOMCL1284, the nearest homologues were distributed among Actinobacteria and other bacterial phyla, including the Chloroflexi, Proteobacteria and Bacteroidetes (Fig. 7). These terpene synthases were clearly separated from the flea beetle proteins and consisted predominantly of germacrene or geosmin synthases (Fig. 7). However, for both clusters of trombidid terpene synthases, the amino acid identity with their nearest bacterial or fungal homologues was low (≤30%). Nevertheless, the majority fulfilled the criteria of Crisp *et al*. [6] as high-confidence ("class A") lateral gene transfers due to the absence of sufficiently closely-related homologues in other Metazoa.

To exclude the possibility that the trombidid terpene synthases were contaminating sequences of bacterial or fungal origin from the environment, or derived from microbial symbionts, we examined the genomic context of each terpene synthase to determine if they were sometimes found adjacent to an incontrovertible metazoan gene. Due to the low contiguity of the *L. deliense* assembly, it was not possible to find other genes on the same contig as a terpene synthase. However, one of the members of ORTHOMCL65 in *D. tinctorium* was located 5.2 kb downstream of a gene with a top BLAST hit to a translational elongation factor-2 mRNA from *Dinothrombium pandorae* [81] (supplemental fig. S5). Furthermore, blob-plot analysis revealed that the GC content and read coverage of the contigs containing terpene synthases lay close to the overall mean for both trombidid genomes (Fig. 8) and the coverage for each gene and its parent scaffold were very similar (supplemental table S8). Importantly, sequences of unambiguous bacterial origin were very rare in both genomes (supplemental table S9), with no evidence for a high-titre symbiont or environmental contaminant that may have impacted significantly on the genome assemblies. Of these candidate laterally-transferred genes in the trombidid mites, expression at the protein level could not be detected in the small *L. deliense* sample. However, in *D. tinctorium*, a high-confidence identification of a single terpene synthase from ORTHOMCL65 was achieved based on two unique peptides, while a third peptide was shared with an additional terpene synthase (supplemental fig. S6).

In addition to these lateral gene transfers, the trombidid mite genomes exhibited further evidence for dynamism in the form of endogenous retroviruses (ERVs). Both genomes showed significant expansions of reverse ribonuclease integrases and Pol polyprotein-like genes, while in *L. deliense* only, a 21-member family of Gag polyprotein-like genes was apparent (supplemental table S5a). Interestingly, the closest homologues of the Gag-like polyproteins in *L. deliense* were found in rodents, bats, lagomorphs, small carnivores, and colugos (a taxon restricted to South-East Asia [82]) (Fig. 9); all of which are known or likely hosts for chigger mites. Unfortunately, the low contiguity of the *L. deliense* genome and the metazoan context of ERVs (with similar GC content to the host) militated against bioinformatic attempts to exclude the possibility of an origin from host contamination; *i.e.*, from squirrel-derived cells on mite mouthparts or in the gut. In contrast, there was no evidence of a vertebrate origin of the Pol polyprotein or integrase genes in *L. deliense*, as they were clearly related to sequences from arachnids and other arthropods (supplemental fig. S7b, S8b).

In the *D. tinctorium* genome, most members of the ERV protein families each clustered in a monophyletic clade, suggesting expansion within the mite genome from a single origin (supplemental fig. S7a, S8a). The closest homologues of the Pol-like polyproteins were found mainly in other arthropods (especially cladocerans and ticks) and more distantly in fungi (supplemental fig. S8a); whereas the reverse ribonuclease integrases were most similar to those from ants, moths, nematodes and other mites (supplemental fig. S7a). A single transposase family was also expanded in the *D. tinctorium* (ORTHOMCL4926; supplemental table S5b), with top BLAST hits in a microsporidian (*Anncaliia algerae* [83]), collembolan (*Orchesella cincta*) and other arthropods. Thus, endogenous retroviruses in *D. tinctorium* might originate from eukaryotic pathogens, larval hosts, prey species, or soil microorganisms, although the fact that some velvet mites feed on other Acari [19] renders phylogenetic analyses of potential lateral gene transfers especially problematic. Uniquely among chelicerate genomes sequenced to date, the *D. tinctorium* genome contained hepatitis D ribozyme-like genes (Rfam RF01787; supplemental table S10), which in *Anopheles* mosquitoes, have been suggested to be involved in processing of non-LTR retrotransposons [84].

Immune system

Many Acari act as vectors of plant or animal pathogens and their life histories expose them to a multitude of microorganisms in their diets and in the environment. Thus, how they interact with pathogens and commensals via their immune system is likely to be a critical aspect determining their success as a group. The canonical humoral immune response gene networks in *Drosophila* are the Toll signalling pathway (responding to β-1,3-glucans from fungi and lysine-type peptidoglycan from Gram-positive bacteria) and the immune deficiency (IMD) pathway (responding to diaminopimelic acid-type peptidoglycan from Gram-negative bacteria). These pathways are activated when upstream transmembrane receptors [peptidoglycan recognition proteins (PGRPs) and β-glucan recognition proteins] bind to the pathogen-derived molecules [85]. Recently, the expanding number of arthropod genomes from outside the class Insecta has highlighted key disparities in the immune pathway genes between the Pancrustacea (Hexapoda and Crustacea) *versus* the Chelicerata and Myriapoda. The most striking difference pertains to the IMD signalling pathway, which was thought to be absent in chelicerates [86]. However, genomic analyses and experimental data from *Ixodes scapularis* have revealed an alternative IMD pathway, in which interactions between IMD and Fas-associated protein with a death domain (both absent in the tick) are complemented by a E3 ubiquitin ligase (X-linked inhibitor of apoptosis protein, XIAP) and its ligand, the E2 conjugating enzyme Bendless [87]. This pathway recognises bacterial-derived lipids and restricts the growth of *Anaplasma phagocytophilum* and *Borrelia burgdorferi* in ticks. Although these data indicate that ticks (and perhaps other Parasitiformes) have a parallel IMD pathway distinct from that characterised in insects, we were unable to identify an XIAP homologue in acariform mites, including the trombidid genomes. This suggests that the evolution of the IMD pathway has diverged along different lineages of the Chelicerata, including the paraphyletic Acari assemblage.

There are two non-exclusive scenarios that can be postulated to explain how the immune system of acariform mites operates in the apparent absence of an IMD pathway. The first is that these taxa might

use the Toll pathway to respond to Gram-negative bacteria as well as Gram-positive bacteria and fungi. Indeed, crosstalk and synergistic immune responses to individual pathogens in *Drosophila* indicate that the two pathways are functionally interconnected even in insects [85], and the IMD pathway may have become redundant during the evolution of acariform mites. Second, expansions in other gene families associated with the immune response may provide alternative pathogen recognition and signalling pathways to tackle Gram-negative bacterial infections. This second scenario is supported in the trombidid mite genomes by large repertoires of Dscam genes (supplemental fig. S9), which have previously been described to have undergone expansions in the Chelicerata and Myriapoda compared to the Pancrustacea [86]. In insects, Dscam is involved in phagocytosis of bacteria by haemocytes, and the *D. melanogaster* Dscam-hv gene exhibits a remarkable capacity to generate >150,000 alternatively-spliced isoforms, perhaps conferring some level of specificity to the insect immune response (although this remains highly controversial [88]). Even relative to other acarine genomes, those of the trombidid mites display a substantially greater complement of Dscam genes (~40 in *D. tinctorium*; supplemental fig. S9), rivalling the 60 gene family members observed in the *Strigamia maritima* (coastal centipede) genome [86, 89].

Several other expanded gene families in one or both of the trombidid mite genomes may have roles in the immune response. In common with *I. scapularis*, these genomes lack the transmembrane PGRPs that are activated in the presence of peptidoglycan in insects, but contain several other PGRP genes with putative extracellular or intracellular roles. However, these soluble PGRP genes are present in larger numbers in the trombidid mite genomes than in those of *I. scapularis* and *T. urticae* (supplemental fig. S10). Since soluble PGRP fragments can have a co-receptor function as shown in insects [90], they might work in concert with as-yet-unidentified components of the acarine immune system to recognise pathogens. This is particularly important in the case of *L. deliense*, as evidence for a peptidoglycan-like structure has recently been reported for *Orientia tsutsugamushi* [91]. Moreover, a much larger expansion in a second class of proteins with putative roles in the immune system, the C-type lectin domain (CTLD) proteins, was apparent in *L. deliense* (supplemental table S5a; Table 1).

The CTLD protein family is a large and diverse group, most members of which do not bind carbohydrates and are thus not lectins [92]. If a CTLD protein does have lectin activity, the carbohydrate-recognition domain usually contains the amino acid motif “WND”, together with “EPN” if the specificity is for mannose, and QPD if the specificity is for galactose. However, several exceptions to this pattern do exist [92]. The expanded *L. deliense* CTLD proteins belong to four orthologous groups containing a total of 88 genes, of which one cluster (ORTHOMCL1094) contains very few genes with signatures of carbohydrate-binding activity (Table 1). The other three groups mainly contain proteins with EPN motifs, suggesting specificity for mannose, although a small proportion of QPD-motif CTLD proteins were apparent in two of the clusters, which might bind galactose (Table 1). The majority of the *L. deliense* CTLD proteins that were predicted to bind carbohydrates exhibited classical or internal secretion signatures, while only a small proportion (10 – 20%) contained transmembrane domains (Table 1). In common with many members of the CTLD protein family, including those in other arthropods, *N*-glycosylation sites were predicted in a significant minority of the *L. deliense* CTLD proteins [93] (Table 1).

Photoreceptor and chemosensory systems

Unlike insects, chelicerates lack compound eyes. Mites and ticks may be eyeless, or can possess one or more pairs of simple dorsal ocelli. The Parasitiformes sequenced to date are all eyeless species (*I. scapularis*, *M. occidentalis* and *T. mercedesae*), whereas the trombidid mites and *T. urticae* have two pairs of ocelli on the prodorsum in the adult stage ([48]. However, the genomes of both eyeless and eyed Acari exhibit a variable complement of opsins, which in combination with the chromophore retinal, form light-sensitive proteins termed rhodopsins. The genomes of eyeless ticks and mites, as well as that of *T. urticae*, contain one or more genes of the “all-*trans*-retinal” peropsin class, which in spiders have been shown to encode non-visual photosensitive pigments with combined G-protein coupled receptor and retinal photoisomerase activity [94]. Since even the eyeless species show evidence for reproductive and diapause behaviours that respond to day-length, it has been suggested

that peropsins are important for the maintenance of circadian rhythms [95, 96]. Notably, we found no evidence of peropsin genes in the trombidid mite genomes, but did find orthologues of *T. urticae* rhodopsin-1 and -7 in both *L. deliense* and *D. tinctorium* (Fig. 10). In the latter, an additional four rhodopsin-7-like paralogues were apparent, three of which were identical at the amino-acid level (Fig. 10).

In contrast with insects but in common with crustaceans and myriapods, the Acari appear to have a scant repertoire of chemosensory protein classes, lacking both odorant-binding proteins (OBPs) and odorant receptors. Moreover, the small chemosensory proteins that have expanded considerably in some insect orders (especially Lepidoptera [97]) are completely absent in the mite genomes, although a gene encoding one such protein was identified in the *I. scapularis* genome (Table 2). Thus, mites rely primarily on gustatory and ionotropic receptors for chemosensation. The repertoire of gustatory receptors (GRs) in *L. deliense* (42 members) and *D. tinctorium* (105 members) was in a similar range to most mites and ticks (albeit from the Parasitiformes) and for the Mandibulata (Table 2); hence, there was no evidence for the massive expansion in this gene family recently reported for the *T. urticae* genome, with almost 700 members [98].

Ionotropic glutamate receptors (iGluRs) are glutamate-gated ion channels that are divided into two subtypes based on sensitivity to N-methyl-D-aspartic acid (NMDA). The canonical iGLuRs do not have direct roles in chemosensation. Rather, at least in *D. melanogaster*, the NMDA-sensitive channels are expressed in the brain and are involved in associative learning and memory [99]. The non-NMDA channels have fundamental roles in synaptic transmission in the neuromuscular junction within muscle tissue or in the nervous system [100], and certain receptor subunits have been shown to be involved in the regulation of sleep (GluR1 [101]) or vision (Clumsy, CG5621, CG3822, CG11155, CG9935 [102, 103]). Strikingly, the *D. tinctorium* genome harboured seven NMDA-type iGluRs and 61 non-NMDA iGluRs, representing substantially greater repertoires than those observed for the *L. deliense, T. urticae* and *D. melanogaster* genomes (especially for the non-NMDA iGluRs) (Fig. 11). The

chemosensory ionotropic receptors (IRs), which exhibit sequence similarity to iGluRs but do not bind glutamate [104], also showed interesting differences in gene family size compared with *T. urticae* and *D. melanogaster.* Notably, while *D. melanogaster* has one gene encoding an IR25a protein, *T. urticae* has three such genes and the trombidid mites have five copies each (Fig. 11). The *D. melanogaster* IR25a is a widely-expressed co-receptor that couples with stimulus-specific IRs to facilitate sensitivity to a diverse range of acids and amines. Recently, IR25a in combination with IR21a and IR93a were demonstrated to function as a thermosensory complex expressed by the dorsal organ cool cells of *D. melanogaster* larvae, which mediates avoidance behaviour to cool temperatures (<20°C) [105, 106]. Sequences that cluster with *D. melanogaster* IR21a and IR93a in the “antennal and first leg” class of IRs were identified in the trombidid mite genomes, with one copy in *D. tinctorium* (as for *T. urticae*) and three copies in *L. deliense* (Fig. 11). Although chelicerates lack antennae, the orthologues of IR93a and/or IR25a have been shown to be highly expressed exclusively in the first pair of legs in *T. mercedesae* [96] and *Varroa destructor* [107], suggesting functional parallels between insects and mites.

Predicted allergens

Although the propensity of chiggers to cause pruritic dermatitis is well recognised in humans and other animals [36-39], the identity of the allergens involved has not been established [108]. The *L. deliense* and *D. tinctorium* genomes were predicted to encode 37 and 33 groups of protein allergens, respectively; substantially more than other sequenced mites in the Acariformes with the exception of the dust mites, *D. farinae* [77] and *E. maynei* [109]. Since velvet mites rarely come into contact with humans, only the *L. deliense* allergens were subjected to further analysis. The *L. deliense* predicted allergen clusters included nine groups that were unique to this species and six that were shared with *D. tinctorium* only (Fig. 12), while a further 28 putative allergen genes in the *L. deliense* genome did not cluster in orthologous groups (supplemental table S11b). The *L. deliense*-unique groups included five distinct clusters of trypsin-like serine proteases and one cluster each of subtilases, papain-like

cysteine proteases, enolases, and cyclophilins, all of which could be classified into recognised allergen families listed in the AllFam database [110] (Fig. 13). The non-clustered allergens belonged to a variety of structural and enzymatic protein groups, but cathepsins, serine proteases and peptidylprolyl isomerases were the most common annotations (supplemental table S11b).

The major allergens in *D. farinae* are the 25-kDa Der f 1, a papain-like cysteine protease; and Der f 2, a 14-kDa uncharacterised protein with a ML (lipid-binding) domain [77]. However, many other minor allergens have been detected by immunoproteomic studies [111, 112] or predicted by homology searches in the *D. farinae* genome [77]. In *L. deliense*, five distinct clusters of papain-like cysteine proteases were identified (AllFam AF030), of which three were shared with *D. farinae,* one was shared only with *D. tinctorium*, and one was unique (Fig. 13). No orthologue of Der f 2 (AF111) was apparent.

Recently, an alpha-enolase has been reported as a novel minor allergen in *D. farinae* [112]. However, the two enolases (AF031) with predicted allergenic properties in the *L. deliense* genome formed an orthologous cluster that was absent from other mites sequenced to date, with homologues in parasitic nematodes and distant chelicerate relatives (*e.g.,* horseshoe crabs; supplemental table S11a). A similar pattern was observed for the cyclophilins (AF038), which have previously been considered a class of allergens restricted to fungal and plant sources [113], although these peptidyl-prolyl *cis-trans* isomerases are universally present across all domains of life. In an immunoproteomic study, a cyclophilin was newly identified as a dust mite allergen, Der f 29 [111], but this was not closely related to the *L. deliense* cyclophilins, which exhibited a greater affinity (~75% identity) to homologues in fungi and fish (supplemental table S11a). The *L. deliense* subtilases (serine proteases with a peptidase S8/S53 domain, AF021) were also absent from other mite genomes but showed 40 – 50% identity to subtilases from fungi and bacteria (supplemental table S11a). This class of proteases have been identified as major allergens produced by ascomycete fungi such as *Curvularia lunata* [114] and *Trichophyton* spp. [115]. Finally, the five clusters of trypsin-like serine proteases (AF024) exhibited closest homologues (40 – 50% identity) in a diverse range of organisms, including *T. urticae* (but too

distant to cluster in ORTHOMCL31), Diptera and scorpions (ORTHOMCL32), fish and lizards (ORTHOMCL88), bugs and ants (ORTHOMCL89), and acorn worms and Diptera (ORTHOMCL90) (supplemental table S11a). Thus, these predicted allergens were distinct from the *D. farinae* molecules classified in AF024 [Der f 3, 6 and 9 [116]], although within ORTHOMCL29, ORTHOMCL30, and ORTHOMCL43, *L. deliense* does possess additional trypsin-like proteases that are orthologous to these *D. farinae* allergens (Figure 13).

A label-free quantitative analysis of protein content in the *L. deliense* indicated that muscle-derived allergens related to the *D. farinae* paramyosin Der f 11 (AF100 [117]) and to *T. urticae* tropomyosin isoforms (AF054) were most abundant (Fig. 13). Although single unique peptides were detected for several *L. deliense*-specific allergen clusters and unclustered allergenic proteins, only one *L. deliense*-specific allergen was present in quantifiable amounts (an enolase in AF031), and this was considerably less abundant than the shared allergens (supplemental table S6a, Fig. 13). However, as allergenicity is not dictated entirely by allergen quantity and can vary markedly between individuals, validating the identity of the most important allergens in chiggers will require screening of sera from trombiculiasis patients.

Putative salivary proteins

Due to the diminutive size of chiggers and the absence of any artificial feeding mechanism for laboratory colonies that might allow collection of saliva, the chigger sialome has not been characterised to date. However, numerous high-throughput studies of tick saliva have been conducted on several genera and multiple lifecycle stages [118-123], and recently an elegant proteomic analysis of *T. urticae* saliva was published, in which mite salivary secretions were collected in an artificial diet substrate [124]. Using proteomic datasets from this *T. urticae* study and a recent *I. scapularis* sialome analysis conducted over several time-points [118], we identified one-to-one orthologues of the salivary proteins from both sources in the tick, spider mite, and trombidid mite genomes. We reasoned that as *T. urticae* is phylogenetically close to the trombidid mites while *I. scapularis* is very distant, protein families shared by the tick and the trombidid mites but not present in the *T. urticae* genome

are likely to represent proteins required for ectoparasitism on animal hosts (as opposed to phytophagy). Indeed, 24 orthologous clusters were shared among the animal ectoparasites but not with *T. urticae*, whereas only five clusters were shared between all mites at the exclusion of the tick (Fig. 14). These 24 animal-ectoparasite clusters are candidates as key salivary components of trombidid mites. An additional two clusters were shared exclusively by *I. scapularis* and *L. deliense,* suggesting that they might be important for feeding on vertebrate hosts (Fig. 14).

To feed successfully, ticks must suppress local immune responses and prevent the clotting of blood. Although trombidid mites feed on tissue exudates or haemolymph rather than blood, and do not feed for as long as some hard tick species, they face similar challenges as ectoparasites that provoke an inflammatory response in their hosts. Interestingly, in accordance with low levels of haem in the trombidid mite diet, we did not find orthologues of tick salivary proteins involved in haem detoxification (ferritins and hemelipoproteins [120]) in the trombidid mite genomes. Several lipocalins with histamine-binding activity have been identified in tick saliva from multiple different species [118, 120, 121], but orthologues of these small proteins were also not present. However, genes encoding two enzymes involved in catabolism of the histamine precursor histidine, urocanate hydratase and formiminotransferase-cyclodeaminase, were detected in both trombidid mite genomes (Table 3). The degradation of histidine feeds into the one carbon pool by folate, and this process is mediated in part by formyltetrahydrofolate dehydrogenase, an enzyme that is also present in multiple copies in the *D. tinctorium* genome (Table 3). While the presence of folate biosynthesis enzymes in tick saliva has been reported previously (and not only for *I. scapularis* [120]), the functional significance of their secretion is unclear. One possibility is that ectoparasitic Acari not only utilise bacterial symbionts as folate "factories" [125, 126], but can scavenge it at source from precursors in their B-vitamin-deficient diets.

Several other protein clusters with potential roles in immune evasion or the regulation of salivation and the ingestion of host fluids were identified in the trombidid mite genomes. The presence of an expanded acetylcholinesterase gene family in tick genomes has been noted previously and

acetylcholinesterases have been detected in the saliva of *Rhipicephalus microplus* [127] and *Amblyomma americanum* [120], as well as *I. scapularis* [118]. It has been proposed that salivary acetylcholinesterases could interfere with cholinergic signalling between host immune cells and might facilitate pathogen establishment [127]. However, the trombidid mites have only a single gene copy each that clusters with the *I. scapularis* acetylcholinesterases (Table 3). Similarly, ATPase inhibitors (Table 3) in saliva could impact on local immune responses [120], since extracellular purine metabolites act as "alarmins" [128]. The massive expansion of sulfotransferases in the *I. scapularis* genome and the secretion of some members of this family in saliva is particularly enigmatic, but recently it has been proposed that sulfotransferases could control salivation and feeding cycles in ticks by sulphating the neurotransmitters dopamine and octopamine [129]. Alternatively or in addition, they might be involved in increasing the activity of small cysteine-free thrombin inhibitors in tick saliva by sulfation of tyrosine residues [130]. Notably, only two of these sulfotransferases were present in each trombidid mite genome compared with >70 members of this family in *I. scapularis* (Table 3).

Of the two salivary protein families restricted to *L. deliense* and *I. scapularis* (Table 3), the secreted trypsin inhibitor-like cysteine-rich domain proteins are among are wide diversity of serine protease inhibitors produced by ticks [131]. This specific class of trypsin inhibitor-like proteins includes ixodidin, an antimicrobial peptide expressed in the haemocytes of *R. microplus* [132] and BmSI-7 and BmSI-6, two peptides from the same species of tick that inhibit cuticle-penetrating proteases secreted by entomopathogenic fungi [133]. The BmSI-7 peptide is expressed in multiple tissues, including the salivary glands [133], but its role in saliva is unknown. However, one possibility is that it helps prevent the tick bite site from becoming infected. In contrast with *I. scapularis*, which harbours 27 trypsin inhibitor-like proteins in its genome, only one orthologue was identified in *L. deliense* (Table 3). The second cluster restricted to *I. scapularis* and *L. deliense*, a signal sequence receptor subunit (Table 3), was unexpected as it has a canonical function in trafficking secretory proteins through the ER [134]. This appears to have a moonlighting role in tick saliva, since it generates strong immune responses in rabbits parasitized by *A. americanum* [120].

Feeding ticks secure their mouthparts in the skin of the host for days or weeks using a cement-like substance that forms a cone in the bite wound. Superficially, the stylostome generated at the feeding site of trombidid mites resembles the tick cement cone, although the structure is tubular (in the Trombiculoidea) or highly branched (in the Trombidioidea) [41]. In both of the trombidid mite genomes, we found an orthologue of a glycine-rich protein present in the sialome of *Rhipicephalus pulchellus* [122] (Table 3). The tick glycine-rich proteins are related to spider silk proteins and form the main structural component of tick cement as determined by proteomic studies [135]. To determine if the trombidid mite genomes may contain other cement-associated proteins not detected in the *I. scapularis* salivary proteomics study [118], we searched for orthologues of all tick cement proteins in the National Center for Biotechnology Information (NCBI) database. We found orthologues of an *I. scapularis* glycine-rich cement protein in both *L. deliense* (one copy) and *D. tinctorium* (three copies) that was distinct from the *R. pulchellus* orthologue; moreover, the velvet mite also possessed an orthologue of a second *I. scapularis* cement protein (supplemental table S12). In addition, both trombidid mites harboured a gene related to a cement protein transcript identified in the sialotranscriptome of *Amblyomma triste* [123] (supplemental table S12). Finally, orthologues of *A. americanum* acidic chitinases involved in conferring stability to the tick cement cone were present in both *D. tinctorium* (four copies) and *L. deliense* (one copy) [136] (supplemental table S12).

Discussion

Genome features and trombidid mite evolution

In this study, we exploited the close phylogenetic relationship between the Trombidioidea and the Trombiculoidea in order to obtain a genome from a single adult velvet mite that could be used to corroborate data derived from a suboptimal trombiculid mite sample (*i.e.*, a pool of engorged larvae). This strategy proved successful because in almost all cases, the unusual features of the trombidid mite genomes were shared between the two sequenced taxa. In contrast with other acariform mites, the trombidid mite genomes were substantially larger, contained a greater proportion of repeats, and

exhibited expansions of mobile elements. These features, coupled with heterozygosity and host contamination in the case of *L. deliense*, proved challenging for accurate genome size estimation but did not prevent the annotation of protein-coding genes, which was sufficient (even for *L. deliense*) for an initial protein expression study.

Obtaining high-quality genomes for the Chelicerata has proved very challenging across multiple orders. The smaller acariform mite species (e.g., *T. urticae*) have compact genomes, but the low DNA yields from individual specimens requires the use of pooling strategies. Conversely, the larger arachnids such as the ticks, spiders and scorpions tend to have very large (several Gb), highly repetitive genomes, sometimes displaying evidence of whole-genome duplication [137]. Recently, several acarine genomes utilising long-read technology have been published. One of these had the advantage of using DNA from a continuous tick (*I. scapularis*) cell line and achieved a BUSCO score of 95% [138]. In contrast, the current draft assembly for the cattle tick (*Rhipicephalus microplus*) used relatively little long-read data in a hybrid assembly and attained a BUSCO gene score of only ~40% (including fragmented genes), although it should be emphasised that the estimated genome size for this species is enormous (7.1 Gb) and it contains 70% repetitive DNA [139]. Finally, a PacBio assembly for the tiny genome (63.2 Mb) of another acariform mite (*Psoroptes ovis*) has been published with a BUSCO score of 91%, but used a pool of >3,000 individuals to obtain sufficient DNA [140]. This would never be a practicable option for chiggers, but in future, a long-read assembly might be possible from a single *D. tinctorium* adult individual due to their massive size.

Importantly, as analyses using the BUSCO Arthropoda gene set consider highly-conserved housekeeping genes that are single-copy in insects (the vast majority of arthropod genomes are from this single class), it will underestimate lineage-specific expansions (rates of gene duplication) between taxa involving novel genes, while flagging duplications of conserved genes in chelicerates that may have a biological origin rather than being assembly artefacts. Elevated duplication rates often signify assembly errors caused by high levels of heterozygosity in the starting material, particularly when

samples are pooled [63]. While DNA extraction from single individuals can mitigate these problems, species with large population sizes may display high heterozygosity at the individual level [141], and/or additional haplotypes may originate from developing embryos or stored sperm. Unfortunately, as *D. tinctorium* is extremely difficult to sex by morphological criteria, it is possible that the individual that we sequenced was a fertilised female, perhaps explaining the high duplication rate flagged by the BUSCO analysis for this genome.

To explore duplication in the *D. tinctorium* genome further, we used the Redundans pipeline [63] and reciprocal BLAST of genomic scaffolds. This demonstrated conclusively that duplication in this genome is not caused by elevated heterozygosity but by identical gene copies located on distinct scaffolds, even if these scaffolds sometimes differed by only a few bases. While assembly errors cannot be excluded in the absence of structural context provided by long-read data, possible biological explanations for this surprising result include recent ectopic recombination of a chromosome segment or chromosome abnormalities (aneuploidy or B chromosomes [142]). This would be compatible with the higher repeat content in this genome compared with that for other acariform mites, which might facilitate large-scale duplications. Notably, whole genome duplication has been reported in other chelicerates, although this is thought to be ancient in the spider-scorpion lineage [137].

As previously reported on publication of the first spider genomes [64], the Acari are polyphyletic, with the superorder Parasitiformes (*i.e.*, the ticks together with mesostigmatid and holothyrid mites) apparently more closely aligned to the spiders (order Araneae) than it is to the Acariformes (Fig. 1). Recent phylogenomic studies have demonstrated that certain arachnid orders, including the Acariformes and Parasitiformes, are evolving at accelerated rates, leading to long-branch attraction artefacts [143]. This can lead to paraphyletic models of arachnid evolution, including the nested placement of the horseshoe crabs (order Xiphosura) within the Arachnida as we observed here, and a node age for divergence from the Araneae (263.2 – 394.7 MYA, Fig. 4) that appears much too recent compared with the oldest xiphosuran fossil, which has been dated to the late Ordovician [144].

However, other key nodes, such as the divergence between the Acariformes and the (Araneae + Parasitiformes), as well as the Araneae-Parasitiformes split, were dated within the range of previous estimates based on the fossil record [145], or more recently, nuclear genomes [64]. While the controversial phylogeny of the Chelicerata at higher taxonomic levels is beyond the scope of our study, at finer scales, the phylogenomic analyses supported the conventional morphology-based taxonomy within the Acariformes, confirming that the trombidid mites are closely related to the phytophagous Tetranychoidea [15]. Moreover, based on an earlier analysis using nuclear and mitochondrial markers [146], our dating of the split between the Tetranychoidea and the Trombidiae is consistent with a secondary radiation within the Trombidiformes that is hypothesised to have occurred during the Carboniferous.

Interestingly, within the Trombidiae, the divergence between the velvet mites and the chiggers apparently occurred much later (~133 MYA) than the emergence of the earliest terrestrial vertebrates (395 MYA [147]), coinciding perhaps with the appearance of crown-group mammals. If this scenario is correct, the ectoparasitism of non-mammalian vertebrates by chiggers that occurs today may be a product of secondary adaptation, as suggested by other authors [21]. Although the fossil record is devoid of trombidid mite specimens predating the Eocene [148], it has been speculated from palaeogeographical and comparative morphological evidence that trombiculid mites fed initially on other arthropods, with larval ectoparasitism on vertebrates evolving during the Palaeocene, leading to an increase in chigger diversity [21]. Our data challenges this hypothesis, because it would imply that something other than host choice in the larval stage drove the split between the Trombidioidea and the Trombiculoidea 60 million years before the latter began feeding on vertebrates. Only the discovery of more ancient trombidid fossils will help to resolve these uncertainties.

Potential roles for terpenes in trombidid mite biology

The most striking finding in the trombidid mite genomes was the presence of large families of laterally-transferred terpene synthases. As the level of amino acid identity between the trombidid terpene

synthases and their closest homologues in microbes is quite low, their end products cannot be inferred with any confidence. However, the question of whether compounds such as 2-methylisoborneol, geosmin or germacrene might confer adaptive advantages to trombidid mites helps to frame hypotheses for experimental testing. Interestingly, all of these compounds are associated with odours and tastes that humans, and some arthropods, may sense as unpleasant or aversive. For instance, 2-methylisoborneol has a musty odour that humans associate with ripe cheeses [149]; whereas geosmin confers the smell of moist soil and is the cause of the muddy, "off" taste that the human olfactory system detects in spoiled water, wine and the flesh of certain freshwater fish [150-152]. More importantly, geosmin released by *Penicillium* spp. or *Streptomyces* spp. on rotting fruit is strongly aversive to *Drosophila* because these organisms produce secondary metabolites that are directly toxic to the fly or to its primary food source, yeast [153]. Germacrene has also been implicated as an arthropod repellent amongst complex sesquiterpene mixtures found in the essential oils of various plants, which have been shown to be effective against several acarines, including the ticks *Rhipicephalus microplus* and *Ixodes ricinus* [154, 155], and the poultry red mite *Dermanyssus gallinae* [155]. Although it has not been assayed in isolation, germacrene is additionally a significant component of essential oils or crude leaf extracts that exhibit toxic effects against phytophagous mites (*Brevipalpus phoenicis* in the Trombidiformes [156]) and ants (*Solenopsis invicta* [157]).

These potential repelling and/or toxic effects of terpenoids would align closely with the apparent aposomatic nature of trombidid mites, most species of which are brightly coloured due to their carotenoid content. These mites have few natural enemies and have been reported to be rapidly regurgitated if offered to predators in the laboratory [158]. However, cannibalism between adults and even ectoparasitism of the free-living stages by trombidid larvae can occur, underlining the relevance of chemical communication within species and between closely related species [19]. To the best of our knowledge, only one report of a parasitoid affecting trombidid mites has been published (the acrocerid fly *Pterodontia flavipes* attacking *Podothrombium* spp. [159]), but the dearth of research on free-living trombidid stages means that no doubt other parasitoids exploiting these hosts do exist. It

is important to note that not all mites are repelled by terpenoid compounds. Many plants use terpenoids as defence compounds to signal to the natural enemies of pest arthropods that the plant is under attack. For example, *Lotus japonicus* infested with *T. urticae* releases several terpenoids, including germacrene, which attract the predatory mite *Phytoseiulus persimilis* [160]. Communication by sex pheromones is also known to occur in *T. urticae*, although molecules other than terpenoids are suspected to mediate this [161]. Moreover, as noted above, neryl formate is an aggregation pheromone in dust mites [76]. In conclusion, while the conferment of a foul taste (and perhaps odour) to the aposomatic trombidids appears to be the most likely evolutionary driver of *de novo* terpenoid synthesis capability, it is also possible that these compounds are used to communicate with potential mates during courtship, or to repel members of the same (or closely-related) species to reduce competition or deter cannibalistic behaviour.

Diapause and seasonality

Many of the putative functions of laterally transferred and/or expanding gene families in the trombidid mites appear to be relevant to the temporal regulation of the lifecycle and the switching of metabolic demands between dormant and active stages. The life history of trombidid mites features alternation between immobile calyptostases (the deutovum, protonymph and tritonymph) and the active instars (larva, deutonymph and adult) [162]. The calyptostases typically persist for 25 – 30 days, while the active stages in temperate species can undergo hibernation over the winter months, including larvae that have not fed until late in the autumn. Diapause of eggs is common in temperate species and can exceed one year in the chigger, *Hirsutiella zachvatkini*, without loss of viability [21]; while larvae of this species can also overwinter on their rodent hosts [163]. The lifecycle of trombidid mites in tropical and subtropical regions has been little studied, but several generations per year are possible [21]. In the case of *Dinothrombium* spp., although the adults are positively phototactic and diurnal if humidity is high (becoming crepuscular during drier conditions), circadian cycles of activity were maintained if mites were transferred to constant darkness in the laboratory [158]. Remarkably,

adult *D. pandorae* of the Californian deserts may only emerge from their burrows during rainstorms to feed and mate for a few hours each year, migrating to the deepest extent of their subterranean refuges during the height of summer [44]. However, overcoming torpor by rapidly adjusting metabolic rate after the cold desert night to the early morning warmth, when termite prey become active, is critical to the lifecycle of *D. pandorae* [44]. It may also be important for chiggers to avoid cool microclimates (and thus maintain peak metabolism) when questing for small mammals, since their hosts are highly motile and a suitable location for attachment must be targeted rapidly before grooming behaviour leads to ingestion of the mite. Hence, the small expansions in the IR repertoire (IR25a, IR21a and IR93a) that we observed in the trombidid mite genomes might reflect more acute sensitivity to cool temperatures than for the phytophagous *T. urticae*.

With relevance to both regulation of metabolism and circadian cycles, the homologues of FLVCR proteins have been little studied in arthropods, but in vertebrates they export haem from the cytoplasm to the extracellular milieu (for FLVCR1) and from mitochondria into the cytoplasm (for FLVCR2) [164]. A FLVCR gene homologue in *Drosophila melanogaster*, *CG1358*, is involved in maintenance of circadian rhythms in the absence of light together with other genes with roles in iron metabolism [165]. Thus, it is intriguing that FLVCR homologues were significantly expanded in the *D. tinctorium* genome but not that of *L. deliense,* as adult chiggers in tropical environments exhibit more regular activity above ground than do *Dinothrombium* spp. [21]. Furthermore, the proteomic analysis of *D. tinctorium* revealed overrepresentation of putative digestive enzymes with peptidase M20 and inhibitor I29 domains, which is consistent with an imperative for adult velvet mites to obtain food reserves rapidly while foraging briefly above ground [44]. An elevated metabolic rate for *L. deliense* larvae was also suggested by the preponderance of mitochondrial enzymes responsible for aerobic energy production in protein extracts. Further studies on trombidid mite metabolism are clearly warranted, as most metabolic studies in the Trombidiformes have focused on winter diapause in *T. urticae* [166], creating a knowledge gap around the physiology of tropical species.

In spider mites, carotenoids are essential for the control of diapause and sexual reproduction. However, reproductive behaviour between the Tetranychoidea and the trombidid mites is radically different, since male *T. urticae* become developmentally arrested in close proximity to dormant female deutonymphs on leaf surfaces. This "guarding" behaviour is stimulated by the intensity of the yellow colouration of the dormant females (derived from their carotenoid pigments), and allows the male to mate with the adult female immediately after ecdysis [167]. In contrast, in order to be inseminated, adult females of trombidid mites must collect a spermatophore deposited on the ground by the male, the location of which is signposted by signalling threads. While colouration is not known to be factor during courtship, the males of some species deposit spermatophores in specially constructed "gardens" and perform encircling dances with the female [19]. However, it has recently been discovered using genetic manipulation of a laterally-transferred phytoene desaturase that carotenoids have a second, distinct function in the regulation of diapause in *T. urticae* [13]. A lack of phytoene desaturase activity not only results in albinism, but prevents overwintering strains from entering diapause, probably due to disrupted light (and thus photoperiod) perception caused by vitamin A deficiency.

To the best of our knowledge, anatomical and experimental studies on trombidid mite vision have not been performed, but spider mites once again provide a closely related template. The eyes of *T. urticae* have been partially characterised, and show biconvex lenses in the anterior pair and simplex convex lenses in the posterior pair [168]. It has been proposed that the anterior eyes respond to UV and green light, whereas the posterior pair are sensitive to UV only. However, laser ablation experiments have demonstrated that either pair can receive sufficient information to control the photoperiodic termination of diapause, while removal of both pairs prevents diapausal exit [169]. In *T. urticae*, the expression pattern of rhodopins in the eyes has not been determined, but in the jumping spider *Hasarius adansoni,* green-sensitive rhodopsin-1 expressed in the lateral ocelli is important for the monochromatic detection of movement [170]. Until recently, the function of rhodopsin-7 was enigmatic, but experiments in *D. melanogaster* have shown that it operates in the circadian

pacemaker neurons of the central brain and is responsible for their highly sensitive response to violet light [171]. Taken together, these experimental findings from other arthropods suggest that trombidid mites might depend on rhodopsin-7 homologues rather than peropsins for control of circadian rhythms (although we failed to identify orthologues of the *Drosophila* clock gene in Acari). The relative roles of FLVCR gene homologues, phytoene desaturases and the rhodopsin pigments in the control of diapause and other life history traits in trombidid mites is evidently a key priority for future experimental studies.

Immune response and vector biology

As trombidid mites are edaphic organisms with a parasitic larval stage, they are exposed to soil microorganisms, the exterior flora of their hosts, and pathogens contained in ingested body fluids. In the case of certain trombiculid mites (especially *Leptotrombidium* spp.), their role as biological vectors of *O. tsutsugamushi* highlights a specific infectious challenge that has resulted from feeding on small mammals. In other arthropods, CTLD proteins with lectin activity act as transmembrane or secreted pattern recognition receptors that bind to carbohydrates on the surface of pathogens. They can function as opsonins in the haemolymph that agglutinate unicellular pathogens and facilitate their phagocytosis by haemocytes [172], or may be expressed on the surface of tissues that form a barrier to infectious assaults, such as in the gut or on the gills of crustaceans [93, 173]. These findings are compatible with roles as secreted opsonins for most of the CTLD proteins identified in the current study, with a smaller number perhaps operating as immune surveillance receptors on the surface of cells or extracellular matrices.

Significant exposure to viral nucleic acids was suggested by the striking diversity of ERV-related sequences in the *L. deliense* genome. Among these, more than 20 Gag-like polyprotein genes of potential mammalian origin were identified. The fact that all other classes of retroviral proteins in the *L. deliense* assembly were closely related to arthropod sequences, and the phylogenetic proximity of the Gag-like polyproteins to ERV elements in non-sciurid mammals, rendered host DNA contamination

in the mite gut or on mouthparts an unlikely source for these sequences; although this possibility cannot be excluded entirely. Despite this caveat, lateral transfers in the distant past originating from mammalian body fluids during the brief ectoparasitic stage is a working hypothesis that can be tested when a more contiguous chigger genome becomes available. In contrast, the horizontal transfer of the long-interspersed element BovB is postulated to have involved an opposite transmission route; that is, between vertebrates by ticks [174].

To the best of our knowledge, no experimental studies on the immune response of trombidid mites have been performed to date. However, a recent study in *T. urticae* involving experimental challenge with bacteria demonstrated that the spider mites were highly susceptible to systemic infection [175]. This contrasted with a more robust response to bacterial challenge in another acariform mite, *Sancassania berlesei*, which unlike *T. urticae* has a saprophytic lifestyle. The authors of this study concluded that the ecology of spider mites, in which all lifecycle stages feed on plant phloem (a relatively aseptic food source), has led to a high degree of susceptibility to pathogen exposure. This failure to overcome infectious insults was associated with an apparent absence of many antimicrobial protein effectors in the spider mite genome. In support of the hypothesis that spider mites have adapted to an environment characterised by very low levels of pathogen challenge, we found that compared with the *L. deliense* genome, the *T. urticae* genome displays a relative paucity of PGRP (supplemental fig. S10), CTLD (supplemental table S5a) and Dscam genes (supplemental fig. S9). Thus, the common ancestor of the Trombidiformes may have harboured a diverse immune gene repertoire that was selectively lost in the branch leading to the Tetranychoidea, and/or the Trombiculoidea have undergone more recent immune gene family expansions. The intermediate immune gene repertoire of the Trombidioidea between that of the spider mites and the chiggers (*D. tinctorium* has considerably fewer CTLD proteins than *L. deliense*) suggests that both immune-related gene losses and gains have occurred during the evolution of the Trombidiformes in response to their radically different natural histories. Indeed, in terms of the degree of exposure to pathogen diversity and abundance, the euedaphic velvet mites are likely to encounter greater infectious challenges than

spider mites, and the feeding behaviour of chiggers on vertebrates is likely to exacerbate this exposure further compared to their relatives that are ectoparasitic on other invertebrates only.

Conclusions

This first analysis of trombidid mite genomes has revealed their dynamic nature relative to those of other acariform mites, including expansions in laterally-transferred gene families and mobile elements. These genomes provide a foundation for fundamental experimental studies on mite immune responses, host-seeking behaviour and feeding, and environmental impacts on lifecycle progression. The function of the laterally-transferred terpene synthases will become a major research theme for chigger biology, as only experimental exposure of chiggers and their potential natural enemies to mite terpene extracts will be able to determine if these unique aspects of secondary metabolism have evolved to attract conspecifics; or conversely, to repel predators, parasitoids and/or competitors. From an applied perspective, the identification of predicted allergens in the *L. deliense* genome sets the scene for immunoproteomic studies of trombiculiasis in both humans and domestic animals, with the potential for immunotherapeutic approaches to be developed as for dust mite allergy [176]. Finally, the successful development of recombinant vaccines against ticks [177] and the promising progress of recombinant vaccine development for both sheep scab [178] and poultry red mite [179] indicate that a similar approach could be explored for chiggers, which has the potential to interrupt, or at least reduce, the transmission of *O. tsutsugamushi* to humans in scrub typhus-endemic areas. Considering the high strain variability of the scrub typhus agent [180], a chigger vaccine utilising mite salivary or gut antigens could provide a much-needed breakthrough against this intractable disease. Thus, a key priority for future research on this enigmatic mites is to obtain organ-specific proteomic data, as whole-body proteomes such as those analysed here may overlook important proteins required for host immunomodulation or pathogen transmission that only reach high concentrations in certain anatomical compartments.

Materials and methods

Sample collection and DNA extraction

Adult specimens of giant red velvet mites were collected within the grounds of the UK Medical Research Council Field Station at Wali Kunda, The Gambia (13°34′N, 14°55′W), in June 2010. Mites were sampled from flowerbeds following heavy rains in June 2010 and stored in 95% ethanol at -80°C. They were identified as *Dinothrombium tinctorium* by Joanna Mąkol (Wrocław University of Environmental and Life Sciences, Poland). Approximately 5 µg of DNA was extracted from a single individual using a Genomic-tip Kit (Qiagen) according to the manufacturer's instructions. Integrity of the DNA was confirmed by agarose gel electrophoresis, which showed a single band of ~20 kb.

For *L. deliense*, engorged larvae were collected from two Berdmore's ground squirrels (*Menetes berdmorei*) captured in Udonthani Province, Thailand, in September 2015. Trapping and euthanasia of small mammals followed the CERoPath (Community Ecology of Rodents and their Pathogens in a changing environment) project protocols [181]. Chiggers were located inside the ears and the inguinal area of the squirrels and stored in absolute ethanol at -20°C. A subsample of the mites was selected and mounted in clearing medium, Berlese fluid (TCS Bioscience, UK), prior to species identification under a compound microscope. Fifty unmounted larvae were pooled and ~30 ng of genomic DNA was extracted using a DNeasy Blood & Tissue Kit (Qiagen) according to the manufacturer's instructions. The DNA was partially degraded, but a dominant band of ~5 kb was apparent by agarose gel electrophoresis.

Library preparation and sequencing

These steps were performed at the Centre for Genomic Research at the University of Liverpool. The *D. tinctorium* DNA was used to generate two Illumina TruSeq libraries and one Nextera mate-pair library. For the former, bead-based size selection using 100 ng and 200 ng of DNA as input into the TruSeq DNA LT Sample Prep Kit with 350 and 550 bp inserts, respectively, was applied. Following eight cycles of amplification, libraries were purified using Agencourt AMPure XP beads (Beckman Coulter). Each library was quantified using a Qubit fluorimeter (Life Technologies) and the size distribution was

assessed using a 2100 Bioanalyzer (Agilent). The final libraries were pooled in equimolar amounts using the Qubit and Bioanalyzer data. The quantity and quality of each pool was assessed on the Bioanalyzer and subsequently by qPCR using the Illumina Library Quantification Kit (KAPA Biosystems) on a LightCycler 480 instrument II (Roche Molecular Diagnostics) according to the manufacturer's instructions. The pool of libraries was sequenced on one lane of the HiSeq 2000 with 2 × 100 bp PE sequencing and v3 chemistry.

The *D. tinctorium* mate-pair library was constructed using the Nextera Mate Pair Kit (Illumina) with 3 kb inserts. The DNA (3 µg) was tagmented as described in the manufacturer's protocol and cleaned using a Genomic DNA Clean & Concentrator column (Zymo Research). The sample was then subjected to strand displacement and cleaned with AMPure XP beads. A 0.6% Certified Megabase Agarose gel (Bio-Rad) was used to separate the fragments, and those in the range of 2 – 5 kb were extracted and recovered using a Zymoclean Large Fragment DNA Recovery Kit (Zymo Research). The recovered DNA was quantified and transferred into a circularisation reaction at 16°C overnight. After purification with AMPure XP beads, DNA was sonicated into ~500 bp fragments using a focused ultrasonicator (Covaris) and recovered with AMPure XP beads as before. Samples were bound to Dynabeads M-280 Streptavidin (Thermo Fisher Scientific) and all subsequent reactions (end repair, A-tailing, and adapter ligation) were bead-based. Samples were amplified with 10 cycles of PCR, recovered by AMPure XP beads at a 1:1 ratio, and quantified using the Qubit dsDNA HS Assay Kit. The library was then subjected to quality control on the Bioanalyzer and LightCycler as for the TruSeq libraries above. The library was sequenced on one run of the MiSeq with 2 × 250 bp PE sequencing.

For *L. deliense*, the DNA sample was sheared to 550 bp using a Bioruptor Pico sonication device (Diagenode) and purified using an AxyPrep FragmentSelect-I Kit (Axygen). The sample was then quantified using a Qubit dsDNA HS Assay Kit on the Qubit fluorimeter, and the size distribution was ascertained on the Bioanalyzer using a High Sensitivity DNA chip (Agilent). The entire sample was used as input material for the NEB Next Ultra DNA Library Preparation Kit. Following nine PCR cycles, the

library was purified using the AxyPrep kit and quantified as before by Qubit. Library size was determined on the Bioanalyzer (Agilent). The quality and quantity of the pool was assessed as described above for *D. tinctorium*. The sequencing was conducted on one lane of an Illumina MiSeq with 2 × 150 bp PE sequencing and v2 chemistry.

Assembly and annotation

For both genomes, base-calling and de-multiplexing of indexed reads was performed by bcl2fastq v. 1.8.4 (Illumina; bcl2fastq, RRID:SCR_015058) to produce sequence data in fastq format. The raw fastq files were trimmed to remove Illumina adapter sequences using Cutadapt version 1.2.1 [182]. The option "-O 3" was set, so the 3' end of any reads which matched the adapter sequence over at least 3 bp was trimmed off. The reads were further trimmed to remove low quality bases, using Sickle version 1.200 [183] with a minimum window quality score of 20. After trimming, reads shorter than 10 bp were removed. If both reads from a pair passed this filter, each was included in the R1 (forward reads) or R2 (reverse reads) file. If only one of a read pair passed this filter, it was included in the R0 (unpaired reads) file.

The Kraken taxonomic sequence classification system (v. 1.0) [184] with the standard (Mini-Kraken) database was used to assign taxonomic annotations to the reads and specifically to estimate the proportion of reads of bacterial origin in the raw data. For genome size estimations, *k*-mers were counted by the Jellyfish program (v. 2.0) [185] and the resultant histograms were uploaded to GenomeScope (v. 1.0) [186] to visualise the *k*-mer distributions.

For *D. tinctorium*, the PE reads were assembled using Abyss (v. 1.5.2) [49, 50], Allpaths-LG (v. r51279) [187] (ALLPATHS-LG, RRID:SCR_010742), SOAPdenovo2 (v. 2.04-r240) [188] (SOAPdenovo2, RRID:SCR_014986) and Discovar (v. r51454) [189]. When running Abyss, *k*-mer sizes were from 35 bp to 80 bp with an interval of 5, and the output "*k*-mer size = 80 bp" was selected as this produced the optimal assembly. Allpaths-LG and Discovar specify *k*-mer size automatically, whereas "*k*-mer size = 63" was selected for SOAPdenovo2, as suggested by the developer. Discovar requires read lengths of

250 bp, so was applied only to the data generated from the mate-pair library. Assessment of the completeness of the genome assemblies was based on the percentage alignment obtained against the reads from the TruSeq 350-bp insert library using bowtie2 (v. 2.0.10) [190] and the predicted core gene content determined by CEGMA (Core Eukaryotic Genes Mapping Approach, RRID:SCR_015055) (v. 2.5) [191]. The final, optimum assembly was created by Abyss (97.4% of scaffolds >500 bp were mapped; 99.2% of Key Orthologs for eukaryotic Genomes were present) and included all scaffolds of ≥1,000 bp, which constituted ~80% of the total length of the raw assembly. The impact of removing small scaffolds was assessed using blobtools (v0.9.19), which generates a GC-coverage plot (proportion of GC bases and node coverage; supplemental fig. S11b) [52]. Read coverage was calculated by mapping the reads onto the final *D. tinctorium* genome assembly. The excluded small scaffolds tended to be high coverage, high GC, and of eukaryotic origin; perhaps representing gut contents (supplemental fig. S11a). We also applied Redundans (v. 0.13c) [63] to identify putative redundant scaffolds in the final *D. tinctorium* assembly with the ‘-noscaffolding’ and ‘-nogapclosing’ options.

For *L. deliense*, a preliminary genome assembly at contig level was performed using Velvet (v. 1.2.07) [51], with parameters of ‘best *k*-mer 99’ and ‘-ins_length 500’. Reads derived from mammalian host genomic DNA were filtered from the preliminary genome assembly using blobtools (v0.9.19) (Fig. 3). Reads mapped on the contigs annotated as Chordata or with a node coverage of <3 were removed, while all others annotated as Arthropoda were retained. The filtered *L. deliense* reads were then reassembled using SPAdes assembler (v3.7.1) [53] (SPAdes, RRID:SCR_000131) with default settings and the length cut-off for scaffolds was set at 500 bp.

To find, classify and mask repeated sequences in the mite genome assemblies, a *de novo* repeat library was first built using RepeatModeler (v. 1.0.8) [192] (RepeatModeler, RRID:SCR_015027) with the ‘-database’ function, followed by application of RepeatMasker (v. 4.0.6) [193] (RepeatMasker, RRID:SCR_012954) using default settings for *de novo* repeated sequences prediction. Then, a

homology-based prediction of repeated sequences in the genome was achieved using RepeatMasker with default settings to search against the RepBase repeat library (issued on August 07, 2015). For non-interspersed repeated sequences, RepeatMasker was run with the '-noint' option, which is specific for simple repeats, microsatellites, and low-complexity repeats.

Thee *ab initio* gene prediction programs, including Augustus (v. 3.2.2) [55] (Augustus: Gene Prediction, RRID:SCR_008417), SNAP (v. 2013-11-29) [56] and GeneMark (v. 2.3e) [57] (GeneMark, RRID:SCR_011930) were used for *de novo* gene predictions in each genome assembly. Augustus and SNAP were trained based on the gene structures generated by CEGMA (v. 2.5) [191], whereas GeneMark [57] was self-trained with the '--BP OFF' option. The three *ab initio* gene prediction programs were run with default settings. We also generated an integrated gene set for each genome assembly using the MAKER v. 2.31.8 [54] pipeline (MAKER, RRID:SCR_005309). The MAKER pipeline runs Augustus, SNAP and GeneMark to produce *de novo* gene predictions, and integrates them with evidence-based predictions. These were generated by aligning the invertebrate RefSeq protein sequences (downloaded on March 31, 2016 from NCBI) to the masked mite genomes by BLASTX. The MAKER pipeline was run with '-RM_off' option to turn all repeat masking options off, and all parameters in control files were left in their default settings. Genes identified by *de novo* prediction, which did not overlap with any genes in the integrated gene sets, were also added to the final gene set for each genome assembly if they could be annotated by InterProScan (v. 4.8) [194] (InterProScan, RRID:SCR_005829) with the InterPro superfamily database (v. 43.1) using '-appl superfamily -nocrc' options.

The Blast2GO pipeline (v. 2.5) [195] (Blast2GO, RRID:SCR_005828) was used to annotate proteins by Gene Ontology (GO) terms. In the first step, all protein sequences were searched against the nr database with BLASTP. The E-value cutoff was set at 1×10^{-6} and the best 20 hits were used for annotation. Based on the BLAST results, the Blast2GO pipeline then predicted the functions of the sequences and assigned GO terms to the BLAST-based annotations. Metabolic pathways were

constructed using KAAS (KEGG Automatic Annotation Server) [196] with the recommended eukaryote sets, all other available insects, and *I. scapularis*. The pathways in which each gene product might be involved were derived from the best KO hit with the BBH (bi-directional best hit) method.

Phylogenetics

Protein data sets of the following arthropod genomes were used as references: *D. melanogaster* (fruit fly; GOS release: 6.11) [197], *A. mellifera* (honey bee; GOS release: 3.2) [198], *T. mercedesae* (bee mite; GOS release: v. 1.0) [96], *T. urticae* (spider mite; GOS release: 20150904) [61], *Stegodyphus mimosarum* (velvet spider; GOS release: 1.0) [64], *I. scapularis* (blacklegged tick; GOS release: 1.4) [199], *M. occidentalis* (predatory mite; GOS release: 1.0) [95], and *Limulus polyphemus* (Atlantic horseshoe crab; GOS release: 2.1.2) [200]. *Caenorhabditis elegans* (nematode; GOS release: WS239) [201] was used as the outgroup. For gene-family phylogenetics, we first aligned orthologous protein sequences with Mafft (v. 7.309) [202] or Kalign (v. 2.0) [203]. We manually trimmed the aligned sequences for large gene sets. The best substitution models of amino-acid substitution were determined for the alignments by ProtTest (v. 3.4) with parameters set to "-all-matrices, -all-distributions, -AIC" [204]. Then, phylogenetic trees were constructed using maximum likelihood methods (PhyML, v. 3.1) [205] (PhyML, RRID:SCR_014629). In addition, a neighbour-joining method was used for building the distance-based trees using MEGA (v. 7.021) [206] (MEGA Software, RRID:SCR_000667).

For species-level phylogenetics, the rapid evolution of acariform mites may challenge phylogenetic analyses due to long-branch attraction [146]. Thus, we used a very strict E-value (1×10^{-50}) when performing a reciprocal BLASTP to exclude the most variant orthologous genes across all genomes tested. The reciprocal BLAST search resulted in identification of a total of 360 highly conserved one-to-one orthologues in all 12 genomes. Each of these orthologous groups was aligned using Mafft with the "-auto" option. These alignments were trimmed by Gblocks (v. 0.91b) [207] and concatenated into unique protein super-alignments. ProtTest determined the best-fit substitution model of LG with

invariant sites (0.133) and gamma distributed rates (0.878) using parameters as above prior to conducting the phylogenetic analysis with PhyML. Based on the topology defined by this phylogenetic analysis, we estimated the divergence time of each species using the Bayesian MCMC method in the PAML package (v. 4.9a) [208] (PAML, RRID:SCR_014932) with the correction of several fossil records (time expressed in MYA): Chelicerata-Mandibulata: 514-636 [209], *T. urticae*-tick-spider: 395–503 [64] (oldest Acari), *A. mellifera-D. melanogaster*: 238-307 [210] and nematode-arthropods: 521–581 [210].

Analysis of gene family expansions

Orthologous gene clusters of *D. tinctorium*, *L. deliense* and the other reference genomes described above were defined based on OrthoMCL (v. 1.4) [211] (OrthoMCL DB: Ortholog Groups of Protein Sequences, RRID:SCR_007839). We used CAFE (v. 3.1) [212] to infer the gene family expansion and contraction in *D. tinctorium* and *L. deliense* against other Acariformes (*T. urticae* and *S. scabiei*). The ultrametric species tree used in the CAFE analyses was created as described for gene-family phylogenetics above.

Analysis of candidate lateral gene transfers

We used a modification of the Crisp method [6] for examination of LGTs in the two mite genomes. Each mite protein dataset was aligned with BLASTP against two databases derived from the NCBI nr database, one consisting of metazoan proteins (excluding proteins from species in the same phylum as the studied species - Arthropoda) and the other of non-metazoan proteins. The LGT index, *h*, was calculated by subtracting the bit-score of the best metazoan match from that of the best non-metazoan match. The genes can be classified into class C if they gained an *h* index $\geq$ 30 and a best non-metazoan bit-score of ≥100. For each class C gene, its average *h* value (h_{orth}) and that of its paralogous genes in each OrthoMCL cluster defined above was determined. If *h* was ≥30, the best non-metazoan bit-score was ≥100, and the h_{orth} value was $\geq$30, the gene was considered to be a class B gene. Class A genes were defined as a subset of class B genes with h ≥30, a best non-metazoan bit-score of ≥100, an h_{orth} value of ≥30, and a best metazoan bitscore <100. The class A criterion that no member of the

orthologous group had a metazoan match with a bitscore ≥100 was not applied, as it was considered too strict for large gene clusters.

Analysis of immune-related gene families

A search for mite immune-related genes was initially preformed with a BLASTP search (E-value, <1 × 10^{-5}) against each mite protein set using immune-related genes defined by Palmer & Jiggins [86]. The identified potentially immune-related genes were then manually checked using BLASTP online at NCBI. For analysis of CTLD proteins, FASTA sequences of proteins in ORTHOMCL223, ORTHOMCL584, ORTHOMCL1095, and ORTHOMCL1094 were analysed by SecretomeP 2.0 [213] (with the "mammalian" option), TMHMM 2.0 [214] and NetNGlyc 1.0 [215] using default settings to identify respective sequence features. Protein sequences were also submitted to InterPro [216] for domain structure analysis. The CTLDs extracted from individual protein sequences were then manually searched for the amino-acid motifs "EPN", "WND" and "QPD".

Analysis of chemosensory and photoreceptor gene families

A search for *D. tinctorium* and *L. deliense* OBPs was initially preformed using TBLASTN (E-value, <1 × 10^{-3}) against their genome assemblies using *D. melanogaster, Drosophila mojavensis, Anopheles gambiae, Bombyx mori, Tribolium castaneum, A. mellifera, Pediculus humanus humanus* and *Acyrthosiphon pisum* OBPs (identified by Vieira and Rozas [217]) as queries. No OBPs were found in the *D. tinctorium* and *L. deliense* genome assemblies. Because OBPs are very divergent in terms of the amino-acid sequences within the family, and the sequence identities between the family members from the different species can be as low as 8% [217], a TBLASTN search has limited power to identify these genes. A search for OBPs was therefore performed again with BLASTP (E-value, <1 × 10^{-3}) to search the automated protein predictions from the mite genome assemblies. A search for small chemosensory proteins in *D. tinctorium* and *L. deliense* was preformed using the same methods as for the OBPs. The query sequences were also based on the study of Vieira and Rozas [217], using chemosensory protein sequences from *D. melanogaster, D. mojavensis, A. gambiae, B. mori, T.*

castaneum, A. mellifera, P. humanus humanus, A. pisum, I. scapularis, and *Daphnia pulex*. For odorant receptors, both TBLASTN and BLASTP searches were performed using *D. melanogaster* and *A. mellifera* sequences (identified by Nozawa and Nei [218] and Robertson and Wanner [219], respectively) as queries.

The *D. tinctorium* and *L. deliense* GR gene families were manually annotated according to TBLASTN and BLASTP searches (both with an E-value cutoff of <1 × 10^{-3}) against their genome assemblies and predicted protein coding genes, respectively, using all *D. melanogaster* [220], *A. mellifera* [219], *I. scapularis* [199], *T. urticae* [98], *T. mercedesae* [96] and *M. occidentalis* [95] GRs as queries. An iterative search was also conducted with termite GRs as queries until no new genes were identified in each major subfamily or lineage. For phylogenetic analysis, *D. tinctorium* and *L. deliense* GRs were aligned with *D. melanogaster* GRs by Kalign [203]with default settings. Poorly-aligned and variable N-terminal and C-terminal regions, as well as several internal regions of highly variable sequences, were excluded from the phylogenetic analysis. Other regions of potentially uncertain alignment between these highly divergent proteins were retained, as removing these regions could potentially compromise subfamily relationships. Based on the trimmed alignment, a PhyML tree was constructed using the substitution model of LG determined by ProtTest [204]. Here, the SH-like local support method was used to assess the significance of phylogenetic clustering.

The *D. tinctorium and L. deliense* iGluRs and IRs were manually annotated according to a TBLASTN (E-value cutoff, <1 × 10^{-3}) search against the *D. tinctorium and L. deliense* genome assemblies using all iGluRs and IRs identified by Croset *et al*. [221] across vertebrates and invertebrates, as well as those identified in the recent *T. mercedesae* genome project [96]. Iterative searches were also conducted with termite iGluRs and IRs as queries until no new genes were identified in each major subfamily or lineage. In the phylogenetic analysis, all manually annotated *D. tinctorium and L. deliense* IRs and iGluRs were aligned with the *D. melanogaster* IRs and iGluRs by Mafft [202] using default settings.

Phylogenetic analysis proceeded as for the GR genes described above, using the more conserved iGluRs to root the tree.

Reference opsin genes were collected based on the work of Nagata *et al*. [94]. Opsin-like sequences in *M. occidentalis, I. scapularis* and *T. urticae* were obtained from the NCBI database. These opsin genes were classified by phylogenetic analysis using the neighbour-joining method with 1,000 bootstraps. The multiple alignment of the amino acid sequences was carried out using Mafft [202] with the '--auto' option. The gaps deletion of the alignment was set to 75% in MEGA7 [206].

Prediction of allergenic gene families

Allergenic protein-coding genes in the genomes of acariform mites (*D. tinctorium*, *L. deliense, T. urticae, D. farinae, S. scabiei,* and *E. maynei*) were predicted using a standalone version of Allerdictor (v. 1.0) [222]. Because the predicted proteome of *D. farinae* is not publicly available, we used protein sequences identified from a new Trinity (v. 2.4) [223] (Trinity, RRID:SCR_013048) assembly for the prediction of allergenic genes. The allergenic gene clusters were constructed using OrthoMCL (v. 1.4) [211] and individual protein sequences were submitted to Pfam (EBI, v.31.0 [224]) to be assigned to protein families. Mapped Pfam domain identifications were then searched against the AllFam database [110] to retrieve corresponding AllFam identifiers for allergen families. Venn diagrams were constructed using InteractiVenn [225].

Salivary and cement protein analysis

A total of 159 non-redundant tick cement proteins were retrieved from the NCBI database and then clustered with all *D. tinctorium* and *L. deliense* amino-acid sequences using OrthoMCL in order to identify the tick cement orthologues in the new genomes. Salivary proteins of *I. scapularis* and *T. urticae* have been previously identified using proteomic methods by Kim *et al*. [118] and Jonckheere *et al*. [124], respectively. Proteins that might be present in the saliva of *D. tinctorium* and *L. deliense* were identified by clustering all predicted protein-coding sequences in the new genomes with these

T. urticae and *I. scapularis* salivary proteins using OrthoMCL [211]. Venn diagrams were constructed using InteractiVenn [225]. To identify putatively secreted proteins, both classical signal peptides and non-classical internal secretion signatures were detected by SecretomeP 2.0 [213] using the "mammalian" option.

Sample preparation for proteomics

Engorged larvae of *L. deliense* (10 specimens) were pooled from infested rodent specimens (*Bandicota indica, Bandicota savilei* and *Rattus tanezumi*) captured across several provinces in Thailand during the field studies of the CERoPath project [181]. The chigger samples were fixed in 70% ethanol and identified as *L. deliense* using autofluorescence microscopy [226]. For *D. tinctorium*, proteomic analysis was performed on a single ethanol-fixed individual from the same collection used for genome sequencing.

The chiggers were washed with chilled 50 mM ammonium bicarbonate. Soluble protein extracts were prepared by homogenisation in 0.1% w/v, Rapigest (Waters) in 50 mM ammonium bicarbonate using a polypropylene mini-pestle. This was followed by three cycles of sonication on ice (Vibra-cell 130PB sonicator at 20 Hz with microprobe; 10 sec of sonication alternating with 30 sec of incubation on ice). Samples were centrifuged at 13,000 × g for 10 min at 4°C. The supernatant was removed and retained. The *D. tinctorium* individual was homogenised using a mini-pestle in lysis buffer (4% SDS, Tris-hydrochloride, pH 7.6) and sonicated and centrifuged as above. Supernatants from both mite preparations were stored at -80°C.

Protein concentrations of the samples were determined using a Bradford protein assay (Thermo Fisher Scientific). The *L. deliense* protein extract was reduced with 3 mM dithiothreitol (Sigma) at 60°C for 10 min, cooled, then alkylated with 9 mM iodoacetamide (Sigma) at room temperature for 30 min in the dark; all steps were performed with intermittent vortex-mixing. Proteomic-grade trypsin (Sigma) was added at a protein:trypsin ratio of 50:1 and incubated at 37°C overnight. Rapigest was removed by adding trifluoroacetic acid (TFA) to a final concentration of 0.5% (v/v). Peptide samples were

centrifuged at 13,000 × g for 30 min to remove precipitated Rapigest. The *D. tinctorium* protein extract was reduced, alkylated and digested with trypsin using the filter-aided sample preparation approach [58]. Peptides from *D. tinctorium* were split into eight fractions using the Pierce High pH Reversed-Phase Peptide Fractionation Kit according to the manufacturer's instructions. Each digest and fraction was concentrated and desalted using C18 Stage tips (Thermo Fisher Scientific), then dried using a centrifugal vacuum concentrator (Eppendorf) and re-suspended in a 0.1% (v/v) TFA, 3% (v/v) acetonitrile solution.

Mass spectrometry

Peptides were analysed by on-line nanoflow LC using the Ultimate 3000 nano system (Dionex/Thermo Fisher Scientific). Samples were loaded onto a trap column (Acclaim PepMap 100, 2 cm × 75 µm inner diameter, C18, 3 µm, 100 Å) at 9µl /min with an aqueous solution containing 0.1 %(v/v) TFA and 2% (v/v) acetonitrile. After 3 min, the trap column was set in-line to an analytical column (Easy-Spray PepMap® RSLC 50 cm × 75 µm inner diameter, C18, 2 µm, 100 Å) fused to a silica nano-electrospray emitter (Dionex). The column was operated at a constant temperature of 35°C and the LC system was coupled to a Q-Exactive mass spectrometer (Thermo Fisher Scientific). Chromatography was performed with a buffer system consisting of 0.1 % formic acid (buffer A) and 80 % acetonitrile in 0.1 % formic acid (buffer B). The peptides were separated by a linear gradient of 3.8 – 50 % buffer B over 90 minutes (*D. tinctorium* and *L. deliense* whole digests) or 30 min (*D. tinctorium* fractions) at a flow rate of 300 nl/min. The Q-Exactive was operated in data-dependent mode with survey scans acquired at a resolution of 70,000 at m/z 200. Scan range was 300 to 2000m/z. Up to the top 10 most abundant isotope patterns with charge states +2 to +5 from the survey scan were selected with an isolation window of 2.0 Th and fragmented by higher energy collisional dissociation with normalized collision energies of 30. The maximum ion injection times for the survey scan and the MS/MS scans were 250 and 50 ms, respectively, and the ion target value was set to 1×10^6 for survey scans and 1×10^4 for

the MS/MS scans. The MS/MS events were acquired at a resolution of 17,500. Repetitive sequencing of peptides was minimized through dynamic exclusion of the sequenced peptides for 20 sec.

Protein identification, quantification and enrichment analysis

Thermo RAW files were imported into Progenesis LC–MS (version 4.1, Nonlinear Dynamics). Peaks were picked by the software using default settings and filtered to include only peaks with a charge state between +2 and +7. Spectral data were converted into .mgf files with Progenesis LC–MS and exported for peptide identification using the Mascot (version 2.3.02, Matrix Science) search engine as described above. Tandem MS data were searched against a database including translated ORFs from either the *D. tinctorium* genome (DinoT_V2_aug2016, 19,258 sequences; 8,386,445 residues) and a contaminant database (cRAP, GPMDB, 2012) (119 sequences; 40,423 residues), or the *L. deliense* genome (L_deliense_V2_Aug16, 15,096 sequences; 5,183,596 residues), the *Rattus norvegicus* genome (UniProt, Apr16 7,948 sequences; 4,022,300 residues) and a contaminant database (cRAP, GPMDB, 2012) (119 sequences; 40,423 residues). The search parameters were as follows: the precursor mass tolerance was set to 10 ppm and the fragment mass tolerance was set as 0.05 Da. Two missed tryptic cleavages were permitted. Carbamidomethylation (cysteine) was set as a fixed modification and oxidation (methionine) set as a variable modification. Mascot search results were further validated using the machine learning algorithm Percolator embedded within Mascot. The Mascot decoy database function was utilised and the false discovery rate was <1%, while individual percolator ion scores >13 indicated identity or extensive homology (*P* <0.05). Mascot search results were imported into Progenesis LC–MS as XML files. Fractions were combined using the Progenesis “combine analysed fractions” workflow. Relative protein abundance was calculated by the Hi-3 default method in Progenesis. Mass spectrometric data were deposited to the ProteomeXchange Consortium (http://proteomecentral.proteomexchange.org) via the PRIDE partner repository [227] with the dataset identifier PXD008346.

Enrichment of protein domains was assessed using Pfam (EBI, v.27.0 [224]) as previously described [228] using the gathering threshold as a cut-off. Briefly, a hypergeometric test for enrichment of Pfam domains in the observed proteome (for identifications supported by ≥2 unique peptides only) relative to the complete search database was performed using R (phyper). The Benjamini & Hochberg step-up false-discovery rate-controlling procedure was applied to the calculated *P* values [229], and enrichment was considered statistically significant where $P < 0.05$.

Availability of data and materials

The datasets supporting the results of this article are available in the GigaDB repository associated with this publication [230]. Raw genomic sequence data have also been submitted to NCBI under BioProjects PRJNA377801 and PRJNA377800 for *L. deliense* and *D. tinctorium*, respectively. Proteomic data for both species are available from the PRIDE repository with the dataset identifier PXD008346.

Abbreviations

CTLD: C-type lectin domain; ERV: endogenous retrovirus; FLVCR: feline leukemia virus subgroup C receptor-related protein; GR: gustatory receptor; IMD: immune deficiency; IR: ionotropic receptor; iGluR: ionotropic glutamate receptors; LGT: lateral gene transfer; MYA: million years ago; NCBI: National Center for Biotechnology Information; OBP: odorant-binding protein; PE: paired-end; PGRP: peptidoglycan recognition protein; TFA: trifluoroacetic acid; XIAP: X-linked inhibitor of apoptosis protein.

Animal ethics

Wild rodents trapped during the CERoPath project [181] and used as a source of chigger material were euthanized by inhaled anaesthetic overdose according to guidelines published by the American Veterinary Medical Association Council on Research [231] and the Canadian Council on Animal Care [232].

Competing interests

The authors declare that they have no competing interests.

Funding

This project was funded by Bayer PLC (Animal Health division) and the University of Liverpool. KC was the recipient of a Mahidol-Liverpool Chamlong Harinasuta Scholarship. The funding bodies had no role in the design of the study; the collection, analysis, and interpretation of data; or in the writing of the manuscript and the decision to publish.

Authors' contributions

Conceptualisation: A.C.D., B.L.M. Formal analysis: X.D., D.X., S.D.A., Y.F., A.C.D. Funding acquisition: J.W.M., A.C.D., B.L.M. Investigation: K.C., S.D.A., M.J.D. Project administration: A.C.D., B.L.M. Supervision: A.C.D., J.W.M., T.K., B.L.M. Validation: X.D., A.C.D., D.X., B.L.M. Visualization: X.D., D.X., S.D.A., A.C.D., B.L.M. Writing (original draft): B.L.M., X.D. Writing (review and editing): B.L.M., X.D., K.C., T.K., M.J.D., D.X., S.D.A., A.C.D. All authors have read and approved the final manuscript.

Acknowledgements

The authors are grateful to Serge Morand (Kasetsart University, Thailand) for managing the CERoPath project field collections that provided chigger material and to Joanna Mąkol (Wrocław University of Environmental and Life Sciences, Poland) for identification of the *D. tinctorium* specimens. We also thank the core research staff at the Centre for Genomic Research (University of Liverpool) for DNA library preparations, sequencing and initial data quality control.

Tables

Table 1: Characteristics of C-type lectin domain proteins in the *Leptotrombidium deliense* genome.

		No. of genes with characteristic			Motifs		
**Cluster**	**No. genes in cluster**	**Secretion (%)[a]**	**Transmembrane domains (%)[b]**	***N*-glycosylation (%)[c]**	**EPN (%)**	**WND (%)**	**QPD (%)**
ORTHOMCL223	32	15 (46.9)	5 (15.6)	6 (18.8)	6 (18.8)	1 (3.1)	1 (3.1)
ORTHOMCL584	22	14 (63.6)	0	1 (4.5)	16 (72.7)	13 (59.1)	1 (4.5)
ORTHOMCL1095	16	10 (62.5)	2 (12.5)	1 (6.3)	11 (68.8)	10 (62.5)	0
ORTHOMCL1094	18	8 (44.4)	3 (16.7)	4 (22.2)	2 (11.1)	2 (11.1)	0

[a]Determined by SecretomeP 2.0 (combined percentage for both classical signal peptides and non-classical internal secretion signatures) [213].

[b]Determined by TMHMM 2.0 [214].

[c]Determined by NetNGlyc 1.0 [215].

Table 2: Comparison of chemosensory receptor repertoires between trombidid mites and 11 other arthropods.

	Chemosensory receptor[a]				
**Species**	**GR**	**OR**	**IR**	**OBP**	**CSP**
D. tinctorium	105	0	7	0	0
L. deliense	42	0	8	0	0
T. urticae	689	0	4	0	0
T. mercedesae	5	0	8	0	0
M. occidentalis	64	0	65	0	0
I. scapularis	60	0	22	0	1
S. maritima	77	0	60	0	2
D. pulex	53	0	85	0	3
D. melanogaster	73	62	66	51	4
A. mellifera	10	163	10	21	6
B. mori	56	48	18	44	18
A. pisum	53	48	11	15	13
P. humanus humanus	8	10	12	5	7

[a]GR, gustatory receptor; OR, olfactory receptor; IR, ionotropic receptor; OBP, odorant-binding protein; CSP, chemosensory protein. Data for other genomes were obtained from [89, 95, 96, 98, 217].

Table 3: Orthologues of *Ixodes scapularis* salivary proteins in trombidid mite genomes.

	**Number of genes in cluster[a]**				
**Orthologous cluster**	***I. scapularis***	***L. deliense***	***D. tinctorium***	**Representative gene ID**	**Representative annotation[b]**
ORTHOMCL5799	1	2 (0)	1 (0)	EEC06447	CNDP dipeptidase
ORTHOMCL8488	1	1 (0)	1 (0)	EEC03480	Uncharacterized protein (peptidase M17, leucine aminopeptidase/peptidase B domain])
ORTHOMCL9	73	2 (0)	2 (0)	EEC08659	Sulfotransferase
ORTHOMCL2940	2	1 (0)	2 (2)	ISCW018873	Short-chain alcohol dehydrogenase
ORTHOMCL8622	1	1 (1)	1 (1)	JAA64643	Glycine-rich cell wall structural protein, partial [*Rhipicephalus pulchellus*]
ORTHOMCL3018	1	1 (0)	3 (0)	EEC14134	Calponin
ORTHOMCL5249	2	1 (0)	1 (0)	JAB84323	Stretch regulated skeletal muscle protein [*Ixodes ricinus*]
ORTHOMCL7670	1	1 (1)	1 (0)	EEC14106	Multifunctional chaperone
ORTHOMCL8293	1	1 (0)	1 (0)	EEC09272	Uncharacterised protein (aspartate dehydrogenase domain)
ORTHOMCL262	5	5 (4)	5 (4)	ISCW002881	UDP-sugar hydrolase
ORTHOMCL2418	1	1 (0)	4 (4)	EEC13206	Cytochrome C
ORTHOMCL5815	2	1 (0)	1 (0)	ISCW011750	Succinyl-CoA ligase beta subunit
ORTHOMCL3911	1	1 (0)	3 (0)	JAB73948	Formyltetrahydrofolate dehydrogenase, partial [*Ixodes ricinus*]
ORTHOMCL3015	2	1 (0)	2 (2)	ISCW001951	ATPase inhibitor
ORTHOMCL8	66	12 (6)	7 (0)	EEC10817	Acyl-CoA synthetase
ORTHOMCL260	13	1 (1)	1 (0)	ISCW001079	Acetylcholinesterase
ORTHOMCL6164	2	1 (0)	1 (0)	ISCW022662	NS1-binding protein
ORTHOMCL4904	2	1 (1)	1 (0)	ISCW006538	60S acidic ribosomal protein LP1
ORTHOMCL907	6	1 (0)	1 (0)	EEC05404	Translation initiation inhibitor UK114/IBM1
ORTHOMCL4900	2	1 (0)	1 (1)	JAB75945	Ribosomal protein LP2 [*Ixodes ricinus*]
ORTHOMCL3820	3	1 (1)	1 (1)	ISCW002028	IMP-GMP specific 5'-nucleotidase
ORTHOMCL4006	1	1 (0)	3 (0)	EEC13628	Urocanate hydratase
ORTHOMCL3946	1	1 (0)	3 (0)	EEC20451	Uncharacterised protein (pseudouridine-5'-phosphate glycosidase domain)

ORTHOMCL5111	2	1 (0)	1 (0)	ISCW010907	Formiminotransferase-cyclodeaminase
ORTHOMCL75	27	1 (0)	0	EEC02489	Cysteine-rich secreted protein (trypsin inhibitor-like cysteine rich domain)
ORTHOMCL8850	2	1 (1)	0	ISCW012704	Signal sequence receptor beta

[a]Numbers in parentheses refer to genes identified with either classical signal peptides or non-classical internal secretion signatures by SecretomeP 2.0 [213].

[b]Annotations are from *I. scapularis* unless otherwise stated. Protein domain information was obtained from the Conserved Domain Database [233]. Tick salivary protein data were obtained from Kim *et al.* [118].

Figure legends

Figure 1: Phylogenetic tree based on the amino acid sequences of 476 one-to-one orthologous genes in 12 species of Ecdysozoa using Bayesian methods. The taxonomy of the trombidid mites follows the scheme of Lindquist *et al*. [15].

Figure 2: Simplified lifecycle of trombidid mites. In the Trombidioidea (velvet mites), the larvae are parasitic on other arthropods; whereas in the Trombiculoidea ("chiggers"), the larvae feed on a variety of vertebrates or (more rarely) other invertebrates. The deutonymph and adult are free-living, edaphic stages that predate soft-bodied arthropods (*e.g.,* termites, springtails and other mites) or consume their eggs. Trombidid eggs are laid in the environment and produce questing larvae that congregate and seek a host. For clarity, only the deutovum and active instars are shown; the protonymph (between the larva and deutonymph stages) and the tritonymph (between the deutonymph and adult stages) are calyptostatic.

Figure 3: Blob-plot of contigs assembled from sequence data derived from engorged *Leptotrombidium deliense* larvae. Blue = Eukaryota; red = Bacteria; white = other; grey = no hit.

Figure 4: Estimated divergence times using a relaxed molecular clock with fossil calibration time and classification of protein-coding genes between 12 species of Ecdysozoa. *Caenorhabditis elegans* was used as the outgroup and the bootstrap value was set as 10,000,000. The 1:1:1 orthologs comprise the common orthologs with the same copy numbers in different species, and the N:N:N orthologs comprise the common orthologs with different copy numbers in these species. Patchy orthologs are shared between more than one, but not all species (excluding those belonging to the previous categories). Unclustered genes are those that cannot be clustered into gene families.

Figure 5: Overrepresented Pfam domains in proteomic datasets generated from a single adult *Dinothrombium tinctorium* and a pool of engorged *Leptotrombidium deliense* larvae. The colour scale represents fold-enrichment.

Figure 6: Phylogeny of terpene synthases from cluster ORTHOMCL65 of trombidid mites alongside related genes from bacteria and fungi. The tree was constructed using a maximum likelihood method; poorly-supported nodes are highlighted in red font.

Figure 7: Phylogeny of terpene synthases from cluster ORTHOMCL1284 of *Leptotrombidium deliense* alongside related genes from bacteria. Metazoan terpene synthases from galerucid beetles are shown as an outgroup. The tree was constructed using a maximum likelihood method; poorly-supported nodes are highlighted in red font.

Figure 8: Blob-plot of read coverage and GC content for terpene synthase genes in *Dinothrombium tinctorium* (Dt) and *Leptotrombidium deliense* (Ld). Terpene synthases (in red, "Bacteria") are shown in relation to all other mite genes (in blue).

Figure 9: Phylogeny of Gag-like polyproteins from *Leptotrombidium deliense* in relation to homologous sequences from small mammals. The tree was constructed using a maximum likelihood method; poorly-supported nodes are highlighted in red font.

Figure 10: Phylogeny and classification of metazoan opsins. The tree was constructed using a neighbour-joining method. Poorly-supported nodes are highlighted in red font.

Figure 11: Phylogeny of *Dinothrombium tinctorium*, *Leptotrombidium deliense*, *Tetranychus urticae* and *Drosophila melanogaster* ionotropic receptors and ionotropic glutamate receptors. The tree was constructed using a maximum-likelihood method.

Figure 12: Venn diagram of orthologous clusters of predicted allergens from six species of acariform mites.

Figure 13: Orthologous clusters of predicted allergens in *Leptotrombidium deliense* as classified by the AllFam database. "Peptides" refers to the number of unique peptides from each allergen detected by mass spectrometric analysis of a pool of *L. deliense* larvae. The heat-map indicates the number of orthologues for each predicted allergen in the genomes of six acariform mites. Ld, *Leptotrombidium*

deliense; Dt, *Dinothrombium tinctorium*; Df, *Dermatophagoides farinae*; Tu, *Tetranychus urticae*; Ss, *Sarcoptes scabiei*; Em, *Euroglyphus maynei*.

Figure 14: Venn diagram of orthologous clusters of putative salivary proteins in four species of Acari. One-to-one orthologues of salivary proteins from *Ixodes scapularis* [118] and *Tetranychus urticae* [124] were identified in the genomes of *Leptotrombidium deliense* and *Dinothrombium tinctorium.* Thumbnail images indicate representative host species.

Additional files

Additional file 1 (.pdf format) contains the following supplemental figures and tables:

Supplemental figure S1: *K*-mer distributions for *Leptotrombidium deliense* (A) and *Dinothrombium tinctorium* (B) plotted by GenomeScope.

Supplemental table S2: Identification of repetitive sequences in the *Dinothrombium tinctorium* and *Leptotrombidium deliense* assemblies compared with other acariform mites.

Supplemental table S3: Impact of Redundans analysis on genome statistics for *Dinothrombium tinctorium.*

Supplemental figure S2: The number of gene families shared among acariform mites (*Dinothrombium tinctorium, Leptotrombidium deliense, Tetranychus urticae* and *Sarcoptes scabiei*); alongside other references including *Drosophila melanogaster, Apis mellifera, Tropilaelaps mercedesae, Metaseiulus occidentalis, Ixodes scapularis, Stegodyphus mimosarum* and *Caenorhabditis elegans* by the OrthoMCL classification algorithm.

Supplemental figure S3: Gene family contraction and expansion in 12 species of Ecdysozoa.

Supplemental figure S4: Phylogeny of carotenoid synthases-cyclases from trombidid mites, spider mites, aphids and fungi.

Supplemental figure S5: Genomic scaffold of *Dinothrombium tinctorium* containing a putative lateral gene transfer adjacent to an incontrovertible mite gene.

Supplemental table S9: Microbial reads identified in the trombidid genomic data by the Kraken taxonomic sequence classification system.

Supplemental figure S6: Peptides detected by mass spectrometry from two terpene synthases in an adult specimen of *Dinothrombium tinctorium*.

Supplemental figure S7: Phylogeny of reverse ribonuclease integrases in *Dinothrombium tinctorium* and their closest homologues in other taxa.

Supplemental figure S8: Phylogeny of Pol-like polyproteins in trombidid mites and their closest homologues in other taxa.

Supplemental figure S9: Phylogeny of Dscam protein-coding sequences in *Dinothrombium tinctorium*, *Leptotrombidium deliense*, *Tetranychus urticae* and *Ixodes scapularis.*

Supplemental figure S10: Phylogeny of peptidoglycan recognition protein sequences in *Dinothrombium tinctorium*, *Leptotrombidium deliense*, *Tetranychus urticae* and *Ixodes scapularis* alongside homologous sequences from insects.

Supplemental table S12: Orthologous clusters of tick cement proteins in the genomes of *Dinothrombium tinctorium* and *Leptotrombidium deliense*.

Supplemental figure S11: Blob-plot of *D. tinctorium* genomic scaffolds before (A) and after (B) removal of small scaffolds (<1,000 bp).

Additional file 2 (.xlsx format) contains the following supplemental tables:

Supplemental table S1: Genome assembly and gene set statistics compared with 14 other arachnids.

Supplemental table S4: Comparative sequence identity and coverage between *D. tinctorium* genomic scaffolds.

Supplemental table S5a: Changes of gene family size in *L. deliense* in comparison with three other acariform mites.

Supplemental table S5b: Changes of gene family size in *D. tinctorium* in comparison with three other acariform mites.

Supplemental table S6a: High-confidence protein identifications and abundance scores for *L. deliense* engorged larvae.

Supplemental table S6b: All protein identifications and abundance scores for *L. deliense* engorged larvae (includes host contamination).

Supplemental table S7: High-confidence protein identifications and abundance scores for a single adult *D. tinctorium* individual.

Supplemental table S8a: Gene- and scaffold-level sequence coverage of scaffolds containing terpene synthase genes for *D. tinctorium*.

Supplemental table S8b: Gene- and scaffold-level sequence coverage of scaffolds containing terpene synthase genes for *L. deliense*.

Supplemental table S10: RNA families identified in the Rfam database in 10 arthropod genomes.

Supplemental table S11a: Predicted allergenic protein clusters in the *L. deliense* genome with top BLAST hit, Pfam domains and AllFam classifications.

Supplemental table S11b: Predicted unclustered allergenic proteins in the *L. deliense* genome with top BLAST hit, Pfam domains and AllFam classifications.

References

1. Krantz GW. Introduction. In: Krantz GW, Walter, D.E., editor. A Manual of Acarology. Lubbock, Texas: Texas Tech University Press; 2009. p. 1-2.

2. Danchin EGJ. Lateral gene transfer in eukaryotes: tip of the iceberg or of the ice cube? BMC Biol. 2016;14.

3. Ku C and Martin WF. A natural barrier to lateral gene transfer from prokaryotes to eukaryotes revealed from genomes: the 70 % rule. BMC Biol. 2016;14:89. doi:10.1186/s12915-016-0315-9.

4. Koutsovoulos G, Kumar S, Laetsch DR, Stevens L, Daub J, Conlon C, et al. No evidence for extensive horizontal gene transfer in the genome of the tardigrade *Hypsibius dujardini*. Proc Natl Acad Sci U S A. 2016;113:5053-8. doi:10.1073/pnas.1600338113.

5. Boothby TC, Tenlen JR, Smith FW, Wang JR, Patanella KA, Nishimura EO, et al. Evidence for extensive horizontal gene transfer from the draft genome of a tardigrade. Proc Natl Acad Sci U S A. 2015;112:15976-81. doi:10.1073/pnas.1510461112.

6. Crisp A, Boschetti C, Perry M, Tunnacliffe A and Micklem G. Expression of multiple horizontally acquired genes is a hallmark of both vertebrate and invertebrate genomes. Genome Biol. 2015;16:50. doi:10.1186/s13059-015-0607-3.

7. Salzberg SL. Horizontal gene transfer is not a hallmark of the human genome. Genome Biol. 2017;18:85. doi:10.1186/s13059-017-1214-2.

8. Acuna R, Padilla BE, Florez-Ramos CP, Rubio JD, Herrera JC, Benavides P, et al. Adaptive horizontal transfer of a bacterial gene to an invasive insect pest of coffee. Proc Natl Acad Sci U S A. 2012;109:4197-202. doi:10.1073/pnas.1121190109.

9. Haegeman A, Jones JT and Danchin EG. Horizontal gene transfer in nematodes: a catalyst for plant parasitism? Mol Plant Microbe Interact. 2011;24:879-87. doi:10.1094/MPMI-03-11-0055.

10. Husnik F, Nikoh N, Koga R, Ross L, Duncan RP, Fujie M, et al. Horizontal gene transfer from diverse bacteria to an insect genome enables a tripartite nested mealybug symbiosis. Cell. 2013;153:1567-78. doi:10.1016/j.cell.2013.05.040.

11. Wybouw N, Pauchet Y, Heckel DG and Van Leeuwen T. Horizontal gene transfer contributes to the evolution of arthropod herbivory. Genome Biol Evol. 2016;8:1785-801. doi:10.1093/gbe/evw119.

12. Wybouw N, Dermauw W, Tirry L, Stevens C, Grbic M, Feyereisen R, et al. A gene horizontally transferred from bacteria protects arthropods from host plant cyanide poisoning. Elife. 2014;3:e02365. doi:10.7554/eLife.02365.

13. Bryon A, Kurlovs AH, Dermauw W, Greenhalgh R, Riga M, Grbic M, et al. Disruption of a horizontally transferred phytoene desaturase abolishes carotenoid accumulation and diapause in *Tetranychus urticae*. Proc Natl Acad Sci U S A. 2017;114:E5871-E80. doi:10.1073/pnas.1706865114.

14. Wybouw N, Van Leeuwen T and Dermauw W. A massive incorporation of microbial genes into the genome of *Tetranychus urticae*, a polyphagous arthropod herbivore. Insect Mol Biol. 2018;27:333-51. doi:10.1111/imb.12374.

15. Lindquist EE, Krantz, G.W., Walter, D.E. Classification. In: Krantz GW, Walter, D.E., editor. A Manual of Acarology. Lubbock, Texas: Texas Tech University Press; 2009. p. 97-103.

16. Snell AE and Heath ACG. Parasitism of mosquitoes (Diptera : Culicidae) by larvae of Arrenuridae and Microtrombidiidae (Acari : Parasitengona) in the Wellington region, New Zealand. New Zeal J Zool. 2006;33:9-15.

17. Mcgarry JW, Gusbi AM, Baker A, Hall MJR and Elmegademi K. Phoretic and parasitic mites infesting the New-World screwworm fly, *Cochliomyia hominivorax*, following sterile insect releases in Libya. Med Vet Entomol. 1992;6:255-60.

18. Goldarazena A and Zhang ZQ. Seasonal abundance of *Allothrombium monochaetum* and *Allothrombium pulvinum* in Navarra-Nafarroa (northern Spain), with notes on larval host preference and rate of parasitism. Exp Appl Acarol. 1999;23:987-93.

19. Zhang ZQ. Biology and ecology of trombidiid mites (Acari : Trombidioidea). Experimental & Applied Acarology. 1998;22:139-55.

20. Zhang ZQ and Xin JL. Biology of *Allothrombium pulvinum* (Acariformes, Trombidiidae), a potential biological-control agent of aphids in China. Experimental & Applied Acarology. 1989;6:101-8.

21. Shatrov AB, Kudryashova, N.I. Taxonomy, life cycles and the origin of parasitism in trombiculid mites. In: Morand S, Krasnov, B.R, Poulin, R, editor. Micromammals and Macroparasites: From Evolutionary Ecology to Management. Tokyo: Springer Japan; 2006. p. 119-40.

22. Nadchatram M. Correlation of habitat, environment and color of chiggers, and their potential significance in the epidemiology of scrub typhus in Malaya (Prostigmata: Trombiculidae). J Med Entomol. 1970;7:131-44.

23. Lakshana P. A new species of trombiculid mite infesting scorpions in Thailand (Acarina, Trombiculidae). J Med Entomol. 1966;3:258-60.

24. Audy JR. Trombiculid mites infesting birds, reptiles, and arthropods in Malaya, with a taxonomic revision, and descriptions of a new genus, two new subgenera, and six new species. Bulletin of the Raffles Museum. 1956;28:27-80.

25. Xu G, Walker DH, Jupiter D, Melby PC and Arcari CM. A review of the global epidemiology of scrub typhus. PLoS Neglect Trop D. 2017;11.

26. Tilak R, Kunwar R, Wankhade UB and Tilak VW. Emergence of *Schoengastiella ligula* as the vector of scrub typhus outbreak in Darjeeling: has *Leptotrombidium deliense* been replaced? Indian J Public Health. 2011;55:92-9. doi:10.4103/0019-557X.85239.

27. Chaisiri K, Stekolnikov AA, Makepeace BL and Morand S. A revised checklist of chigger mites (Acari: Trombiculidae) from Thailand, with the description of three new species. J Med Entomol. 2016;53:321-42. doi:10.1093/jme/tjv244.

28. Taylor AJ, Paris DH and Newton PN. A systematic review of mortality from untreated scrub typhus (*Orientia tsutsugamushi*). PLoS Negl Trop Dis. 2015;9:e0003971. doi:10.1371/journal.pntd.0003971.

29. Bonell A, Lubell Y, Newton PN, Crump JA and Paris DH. Estimating the burden of scrub typhus: A systematic review. PLoS Negl Trop Dis. 2017;11:e0005838. doi:10.1371/journal.pntd.0005838.

30. Weitzel T, Dittrich S, Lopez J, Phuklia W, Martinez-Valdebenito C, Velasquez K, et al. Endemic scrub typhus in South America. N Engl J Med. 2016;375:954-61. doi:10.1056/NEJMoa1603657.

31. Izzard L, Fuller A, Blacksell SD, Paris DH, Richards AL, Aukkanit N, et al. Isolation of a novel *Orientia* species (*O. chuto* sp. nov.) from a patient infected in Dubai. J Clin Microbiol. 2010;48:4404-9. doi:10.1128/JCM.01526-10.

32. Maina AN, Farris CM, Odhiambo A, Jiang J, Laktabai J, Armstrong J, et al. Q fever, scrub typhus, and rickettsial diseases in children, Kenya, 2011-2012. Emerg Infect Dis. 2016;22:883-6. doi:10.3201/eid2205.150953.

33. Yu XJ and Tesh RB. The role of mites in the transmission and maintenance of Hantaan virus (Hantavirus: Bunyaviridae). J Infect Dis. 2014;210:1693-9. doi:10.1093/infdis/jiu336.

34. Kabeya H, Colborn JM, Bai Y, Lerdthusnee K, Richardson JH, Maruyama S, et al. Detection of *Bartonella tamiae* DNA in ectoparasites from rodents in Thailand and their sequence similarity with bacterial cultures from Thai patients. Vector-Borne Zoonot. 2010;10:429-34.

35. Huang Y, Zhao L, Zhang Z, Liu M, Xue Z, Ma D, et al. Detection of a novel *Rickettsia* from *Leptotrombidium scutellare* mites (Acari: Trombiculidae) from Shandong of China. J Med Entomol. 2017;54:544-9. doi:10.1093/jme/tjw234.

36. Smith GA, Sharma V, Knapp JF and Shields BJ. The summer penile syndrome: seasonal acute hypersensitivity reaction caused by chigger bites on the penis. Pediatr Emerg Care. 1998;14:116-8.

37. Little SE, Carmichael KP and Rakich PM. Trombidiosis-induced dermatitis in white-tailed deer (*Odocoileus virginianus*). Vet Pathol. 1997;34:350-2. doi:10.1177/030098589703400412.

38. Leone F, Di Bella A, Vercelli A and Cornegliani L. Feline trombiculosis: a retrospective study in 72 cats. Vet Dermatol. 2013;24:535-e126. doi:10.1111/vde.12053.

39. Faccini JL, Santos AC, Santos SB, Jacinavicius FC, Bassini-Silva R and Barros-Battesti DM. Trombiculiasis in domestic goats and humans in the state of Maranhao, Brazil. Rev Bras Parasitol Vet. 2017;26:104-9. doi:10.1590/S1984-29612016088.

40. Shatrov AB. Stylostome formation in trombiculid mites (Acariformes: Trombiculidae). Exp Appl Acarol. 2009;49:261-80.

41. Shatrov AB and Felska M. Comparative stylostome ultrastructure of *Hirsutiella zachvatkini* (Trombiculidae) and *Trombidium holosericeum* (Trombidiidae) larvae. Exp Appl Acarol. 2017;72:339-65.

42. Minter DM. Some field and laboratory observations on the British harvest mite, *Trombicula autumnalis* Shaw. Parasitology. 1957;47:185-93.

43. Singer G. *Neotrombidium leonardi* (Acarina: Trombidioidea), Pt. 2 : Biology of *N. beeri* and other species of the genus. Acarologia. 1971;13:143-51.

44. Tevis L and Newell IM. Studies on biology and seasonal cycle of giant red velvet mite, *Dinothrombium pandorae* (Acari, Trombidiidae). Ecology. 1962;43:497-505.

45. Zhang ZQ. Notes on the occurrence and distribution of the biocontrol agent, *Allothrombium pulvinum* Ewing (Acari, Trombidiidae), in a peach orchard in China. J Appl Entomol. 1992;113:13-7.

46. Wiggins GJ, Grant JF and Welbourn WC. *Allothrombium mitchelli* (Acari : Trombidiidae) in the Great Smoky Mountains National Park: Incidence, seasonality, and predation on beech scale (Homoptera : Eriococcidae). Ann Entomol Soc Am. 2001;94:896-901.

47. Traub R and Wisseman CL, Jr. Ecological considerations in scrub typhus. 2. Vector species. Bull World Health Organ. 1968;39:219-30.

48. Walter DE, Lindquist, E.E., Smith, I.M., Cook, D.R., Krantz, G.W. Order Trombidiformes. In: Krantz GW, Walter, D.E., editor. A Manual of Acarology. Lubbock, Texas: Texas Tech University Press; 2009. p. 233-419.

49. Simpson JT, Wong K, Jackman SD, Schein JE, Jones SJM and Birol I. ABySS: A parallel assembler for short read sequence data. Genome Res. 2009;19:1117-23.

50. Robertson G, Schein J, Chiu R, Corbett R, Field M, Jackman SD, et al. De novo assembly and analysis of RNA-seq data. Nat Methods. 2010;7:909-U62.

51. Zerbino DR and Birney E. Velvet: Algorithms for de novo short read assembly using de Bruijn graphs. Genome Res. 2008;18:821-9.

52. Kumar S, Jones M, Koutsovoulos G, Clarke M and Blaxter M. Blobology: exploring raw genome data for contaminants, symbionts and parasites using taxon-annotated GC-coverage plots. Front Genet. 2013;4:237. doi:10.3389/fgene.2013.00237.

53. Bankevich A, Nurk S, Antipov D, Gurevich AA, Dvorkin M, Kulikov AS, et al. SPAdes: a new genome assembly algorithm and its applications to single-cell sequencing. J Comput Biol. 2012;19:455-77. doi:10.1089/cmb.2012.0021.

54. Cantarel B, Korf I, Robb S, Parra G, Ross E, Moore B, et al. MAKER: An easy-to-use annotation pipeline designed for emerging model organism genomes. Genome Research. 2008;18:188-96. doi:10.1101/gr.6743907.

55. Stanke M and Morgenstern B. AUGUSTUS: a web server for gene prediction in eukaryotes that allows user-defined constraints. Nucleic Acids Res. 2005;33:W465-7. doi:10.1093/nar/gki458.

56. Korf I. Gene finding in novel genomes. BMC Bioinformatics. 2004;5:59. doi:10.1186/1471-2105-5-59.

57. Lukashin AV and Borodovsky M. GeneMark.hmm: new solutions for gene finding. Nucleic Acids Res. 1998;26:1107-15.

58. Wisniewski JR, Zougman A, Nagaraj N and Mann M. Universal sample preparation method for proteome analysis. Nat Methods. 2009;6:359-62. doi:10.1038/nmeth.1322.

59. Kim JH, Roh JY, Kwon DH, Kim YH, Yoon KA, Yoo S, et al. Estimation of the genome sizes of the chigger mites *Leptotrombidium pallidum* and *Leptotrombidium scutellare* based on quantitative PCR and k-mer analysis. Parasit Vectors. 2014;7:279. doi:10.1186/1756-3305-7-279.

60. Rider SD, Jr., Morgan MS and Arlian LG. Draft genome of the scabies mite. Parasit Vectors. 2015;8:585. doi:10.1186/s13071-015-1198-2.

61. Grbic M, Van Leeuwen T, Clark RM, Rombauts S, Rouze P, Grbic V, et al. The genome of *Tetranychus urticae* reveals herbivorous pest adaptations. Nature. 2011;479:487-92. doi:10.1038/nature10640.

62. Simao FA, Waterhouse RM, Ioannidis P, Kriventseva EV and Zdobnov EM. BUSCO: assessing genome assembly and annotation completeness with single-copy orthologs. Bioinformatics. 2015;31:3210-2. doi:10.1093/bioinformatics/btv351.

63. Pryszcz LP and Gabaldon T. Redundans: an assembly pipeline for highly heterozygous genomes. Nucleic Acids Res. 2016;44:e113. doi:10.1093/nar/gkw294.

64. Sanggaard KW, Bechsgaard JS, Fang X, Duan J, Dyrlund TF, Gupta V, et al. Spider genomes provide insight into composition and evolution of venom and silk. Nat Commun. 2014;5:3765. doi:10.1038/ncomms4765.

65. Ayme-Southgate A, Lasko P, French C and Pardue ML. Characterization of the gene for mp20: a *Drosophila* muscle protein that is not found in asynchronous oscillatory flight muscle. J Cell Biol. 1989;108:521-31.

66. Huang Y, Li W, Huang L, Hu Y, Chen W, Wang X, et al. Identification and characterization of myophilin-like protein: a life stage and tissue-specific antigen of *Clonorchis sinensis*. Parasitol Res. 2012;111:1143-50. doi:10.1007/s00436-012-2946-2.

67. Friedrich MV, Schneider M, Timpl R and Baumgartner S. Perlecan domain V of *Drosophila melanogaster*. Sequence, recombinant analysis and tissue expression. Eur J Biochem. 2000;267:3149-59.

68. Klinkowstrom AM, Terra WR and Ferreira C. Midgut dipeptidases from *Rhynchosciara americana* (Diptera) larvae - properties of soluble and membrane-bound forms. Insect Biochem Molec. 1995;25:303-10.

69. Long JZ, Svensson KJ, Bateman LA, Lin H, Kamenecka T, Lokurkar IA, et al. The secreted enzyme PM20D1 regulates lipidated amino acid uncouplers of mitochondria. Cell. 2016;166:424-35. doi:10.1016/j.cell.2016.05.071.

70. Shahein YE, El-Rahim MT, Hussein NA, Hamed RR, El-Hakim AE and Barakat MM. Molecular cloning of a small heat shock protein (sHSPII) from the cattle tick *Rhipicephalus (Boophilus) annulatus* salivary gland. Int J Biol Macromol. 2010;47:614-22. doi:10.1016/j.ijbiomac.2010.08.006.

71. Matsumoto I, Watanabe H, Abe K, Arai S and Emori Y. A putative digestive cysteine proteinase from *Drosophila melanogaster* is predominantly expressed in the embryonic and larval midgut. Eur J Biochem. 1995;227:582-7.

72. Santamaria ME, Hernandez-Crespo P, Ortego F, Grbic V, Grbic M, Diaz I, et al. Cysteine peptidases and their inhibitors in *Tetranychus urticae*: a comparative genomic approach. BMC Genomics. 2012;13:307. doi:10.1186/1471-2164-13-307.

73. Santamaria ME, Gonzalez-Cabrera J, Martinez M, Grbic V, Castanera P, Diaz L, et al. Digestive proteases in bodies and faeces of the two-spotted spider mite, *Tetranychus urticae*. J Insect Physiol. 2015;78:69-77. doi:10.1016/j.jinsphys.2015.05.002.

74. Kuwahara Y, Ichiki Y, Morita M, Tanabe T and Asano Y. Chemical polymorphism in defense secretions during ontogenetic development of the millipede *Niponia nodulosa*. J Chem Ecol. 2015;41:15-21.

75. Kenny NJ, Shen X, Chan TT, Wong NW, Chan TF, Chu KH, et al. Genome of the rusty millipede, *Trigoniulus corallinus*, illuminates diplopod, myriapod, and arthropod evolution. Genome Biol Evol. 2015;7:1280-95. doi:10.1093/gbe/evv070.

76. Skelton AC, Cameron MM, Pickett JA and Birkett MA. Identification of neryl formate as the airborne aggregation pheromone for the American house dust mite and the European house dust mite (Acari: Epidermoptidae). J Med Entomol. 2010;47:798-804.

77. Chan TF, Ji KM, Yim AK, Liu XY, Zhou JW, Li RQ, et al. The draft genome, transcriptome, and microbiome of *Dermatophagoides farinae* reveal a broad spectrum of dust mite allergens. J Allergy Clin Immunol. 2015;135:539-48. doi:10.1016/j.jaci.2014.09.031.

78. Beran F, Rahfeld P, Luck K, Nagel R, Vogel H, Wielsch N, et al. Novel family of terpene synthases evolved from trans-isoprenyl diphosphate synthases in a flea beetle. Proc Natl Acad Sci U S A. 2016;113:2922-7. doi:10.1073/pnas.1523468113.

79. Faddeeva-Vakhrusheva A, Kraaijeveld K, Derks MFL, Anvar SY, Agamennone V, Suring W, et al. Coping with living in the soil: the genome of the parthenogenetic springtail *Folsomia candida*. BMC Genomics. 2017;18:493. doi:10.1186/s12864-017-3852-x.

80. Komatsu M, Tsuda M, Omura S, Oikawa H and Ikeda H. Identification and functional analysis of genes controlling biosynthesis of 2-methylisoborneol. Proc Natl Acad Sci U S A. 2008;105:7422-7. doi:10.1073/pnas.0802312105.

81. Regier JC, Shultz JW, Zwick A, Hussey A, Ball B, Wetzer R, et al. Arthropod relationships revealed by phylogenomic analysis of nuclear protein-coding sequences. Nature. 2010;463:1079-83. doi:10.1038/nature08742.

82. Mason VC, Li G, Minx P, Schmitz J, Churakov G, Doronina L, et al. Genomic analysis reveals hidden biodiversity within colugos, the sister group to primates. Sci Adv. 2016;2:e1600633. doi:10.1126/sciadv.1600633.

83. Parisot N, Pelin A, Gasc C, Polonais V, Belkorchia A, Panek J, et al. Microsporidian genomes harbor a diverse array of transposable elements that demonstrate an ancestry of horizontal exchange with metazoans. Genome Biol Evol. 2014;6:2289-300. doi:10.1093/gbe/evu178.

84. Webb CH and Luptak A. HDV-like self-cleaving ribozymes. RNA Biol. 2011;8:719-27. doi:10.4161/rna.8.5.16226.

85. Valanne S, Wang JH and Ramet M. The *Drosophila* Toll signaling pathway. J Immunol. 2011;186:649-56. doi:10.4049/jimmunol.1002302.

86. Palmer WJ and Jiggins FM. Comparative genomics reveals the origins and diversity of arthropod immune systems. Mol Biol Evol. 2015;32:2111-29. doi:10.1093/molbev/msv093.

87. Shaw DK, Wang X, Brown LJ, Chavez AS, Reif KE, Smith AA, et al. Infection-derived lipids elicit an immune deficiency circuit in arthropods. Nat Commun. 2017;8:14401. doi:10.1038/ncomms14401.

88. Brites D and Du Pasquier L. Somatic and germline diversification of a putative immunoreceptor within one phylum: Dscam in arthropods. Results Probl Cell Differ. 2015;57:131-58. doi:10.1007/978-3-319-20819-0_6.

89. Chipman AD, Ferrier DE, Brena C, Qu J, Hughes DS, Schroder R, et al. The first myriapod genome sequence reveals conservative arthropod gene content and genome organisation in the centipede *Strigamia maritima*. PLoS Biol. 2014;12:e1002005. doi:10.1371/journal.pbio.1002005.

90. Kaneko T, Yano T, Aggarwal K, Lim JH, Ueda K, Oshima Y, et al. PGRP-LC and PGRP-LE have essential yet distinct functions in the *Drosophila* immune response to monomeric DAP-type peptidoglycan. Nat Immunol. 2006;7:715-23. doi:10.1038/ni1356.

91. Atwal S, Giengkam S, Chaemchuen S, Dorling J, Kosaisawe N, VanNieuwenhze M, et al. Evidence for a peptidoglycan-like structure in *Orientia tsutsugamushi*. Mol Microbiol. 2017;105:440-52. doi:10.1111/mmi.13709.

92. Zelensky AN and Gready JE. The C-type lectin-like domain superfamily. FEBS J. 2005;272:6179-217. doi:10.1111/j.1742-4658.2005.05031.x.

93. Alenton RR, Koiwai K, Miyaguchi K, Kondo H and Hirono I. Pathogen recognition of a novel C-type lectin from *Marsupenaeus japonicus* reveals the divergent sugar-binding specificity of QAP motif. Sci Rep. 2017;7:45818. doi:10.1038/srep45818.

94. Nagata T, Koyanagi M, Tsukamoto H and Terakita A. Identification and characterization of a protostome homologue of peropsin from a jumping spider. J Comp Physiol A. 2010;196:51-9.

95. Hoy MA, Waterhouse RM, Wu K, Estep AS, Ioannidis P, Palmer WJ, et al. Genome sequencing of the phytoseiid predatory mite *Metaseiulus occidentalis* reveals completely atomized *hox* genes and superdynamic intron evolution. Genome Biology and Evolution. 2016;8:1762-75.

96. Dong X, Armstrong SD, Xia D, Makepeace BL, Darby AC and Kadowaki T. Draft genome of the honey bee ectoparasitic mite, *Tropilaelaps mercedesae*, is shaped by the parasitic life history. Gigascience. 2017;6: 1–17.

97. Ozaki K, Utoguchi A, Yamada A and Yoshikawa H. Identification and genomic structure of chemosensory proteins (CSP) and odorant binding proteins (OBP) genes expressed in foreleg tarsi of the swallowtail butterfly *Papilio xuthus*. Insect Biochem Molec. 2008;38:969-76.

98. Ngoc PCT, Greenhalgh R, Dermauw W, Rombauts S, Bajda S, Zhurov V, et al. Complex evolutionary dynamics of massively expanded chemosensory receptor families in an extreme generalist chelicerate herbivore. Genome Biology and Evolution. 2016;8:3323-39.

99. Xia SZ, Miyashita T, Fu TF, Lin WY, Wu CL, Pyzocha L, et al. NMDA receptors mediate olfactory learning and memory in *Drosophila*. Curr Biol. 2005;15:603-15.

100. Frank CA. Homeostatic plasticity at the *Drosophila* neuromuscular junction. Neuropharmacology. 2014;78:63-74.

101. Robinson JE, Paluch J, Dickman DK and Joiner WJ. ADAR-mediated RNA editing suppresses sleep by acting as a brake on glutamatergic synaptic plasticity. Nature Communications. 2016;7.

102. Karuppudurai T, Lin TY, Ting CY, Pursley R, Melnattur KV, Diao FQ, et al. A hard-wired glutamatergic circuit pools and relays UV signals to mediate spectral preference in *Drosophila*. Neuron. 2014;81:603-15.

103. Hu W, Wang TT, Wang X and Han JH. I-h channels control feedback regulation from amacrine cells to photoreceptors. PLoS Biology. 2015;13.

104. Rytz R, Croset V and Benton R. Ionotropic Receptors (IRs): Chemosensory ionotropic glutamate receptors in *Drosophila* and beyond. Insect Biochem Molec. 2013;43:888-97.

105. Ni L, Klein M, Svec KV, Budelli G, Chang EC, Ferrer AJ, et al. The ionotropic receptors IR21a and IR25a mediate cool sensing in *Drosophila.* Elife. 2016;5.

106. Knecht ZA, Silbering AF, Ni LN, Klein M, Budelli G, Bell R, et al. Distinct combinations of variant ionotropic glutamate receptors mediate thermosensation and hygrosensation in *Drosophila*. Elife. 2016;5.

107. Eliash N, Singh NK, Thangarajan S, Sela N, Leshkowitz D, Kamer Y, et al. Chemosensing of honeybee parasite, *Varroa destructor*: Transcriptomic analysis. Sci Rep-Uk. 2017;7:13091.

108. Wright SM, Wikel SK and Wrenn WJ. Host immune responsiveness to the chigger, *Eutrombicula cinnabaris*. Ann Trop Med Parasit. 1988;82:283-93.

109. Rider SD, Jr., Morgan MS and Arlian LG. Allergen homologs in the *Euroglyphus maynei* draft genome. PLoS One. 2017;12:e0183535. doi:10.1371/journal.pone.0183535.

110. Radauer C, Bublin M, Wagner S, Mari A and Breiteneder H. Allergens are distributed into few protein families and possess a restricted number of biochemical functions. J Allergy Clin Immunol. 2008;121:847-52 e7. doi:10.1016/j.jaci.2008.01.025.

111. An S, Chen L, Long C, Liu X, Xu X, Lu X, et al. *Dermatophagoides farinae* allergens diversity identification by proteomics. Mol Cell Proteomics. 2013;12:1818-28. doi:10.1074/mcp.M112.027136.

112. Choopong J, Reamtong O, Sookrung N, Seesuay W, Indrawattana N, Sakolvaree Y, et al. Proteome, allergenome, and novel allergens of house dust mite, *Dermatophagoides farinae*. J Proteome Res. 2016;15:422-30. doi:10.1021/acs.jproteome.5b00663.

113. Fluckiger S, Fijten H, Whitley P, Blaser K and Crameri R. Cyclophilins, a new family of cross-reactive allergens. Eur J Immunol. 2002;32:10-7. doi:10.1002/1521-4141(200201)32:1<10::AID-IMMU10>3.0.CO;2-I.

114. Tripathi P, Nair S, Singh BP and Arora N. Molecular and immunological characterization of subtilisin like serine protease, a major allergen of *Curvularia lunata*. Immunobiology. 2011;216:402-8. doi:10.1016/j.imbio.2010.06.009.

115. Woodfolk JA, Wheatley LM, Piyasena RV, Benjamin DC and Platts-Mills TA. *Trichophyton* antigens associated with IgE antibodies and delayed type hypersensitivity. Sequence homology to two families of serine proteinases. J Biol Chem. 1998;273:29489-96.

116. Teng F, Yu L, Bian Y, Sun J, Wu J, Ling C, et al. In silico structural analysis of group 3, 6 and 9 allergens from *Dermatophagoides farinae*. Mol Med Rep. 2015;11:3559-64. doi:10.3892/mmr.2015.3166.

117. Tsai LC, Chao PL, Hung MW, Sun YC, Kuo IC, Chua KY, et al. Protein sequence analysis and mapping of IgE and IgG epitopes of an allergenic 98-kDa *Dermatophagoides farinae* paramyosin, Der f 11. Allergy. 2000;55:141-7.

118. Kim TK, Tirloni L, Pinto AF, Moresco J, Yates JR, 3rd, da Silva Vaz I, Jr., et al. *Ixodes scapularis* Tick saliva proteins sequentially secreted every 24 h during blood feeding. PLoS Negl Trop Dis. 2016;10:e0004323. doi:10.1371/journal.pntd.0004323.

119. Tirloni L, Kim TK, Pinto AFM, Yates JR, 3rd, da Silva Vaz I, Jr. and Mulenga A. Tick-host range adaptation: changes in protein profiles in unfed adult *Ixodes scapularis* and *Amblyomma americanum* saliva stimulated to feed on different hosts. Front Cell Infect Microbiol. 2017;7:517. doi:10.3389/fcimb.2017.00517.

120. Radulovic ZM, Kim TK, Porter LM, Sze SH, Lewis L and Mulenga A. A 24-48 h fed *Amblyomma americanum* tick saliva immuno-proteome. BMC Genomics. 2014;15:518. doi:10.1186/1471-2164-15-518.

121. Esteves E, Maruyama SR, Kawahara R, Fujita A, Martins LA, Righi AA, et al. Analysis of the salivary gland transcriptome of unfed and partially fed *Amblyomma sculptum* ticks and descriptive proteome of the saliva. Front Cell Infect Microbiol. 2017;7:476. doi:10.3389/fcimb.2017.00476.

122. Tan AW, Francischetti IM, Slovak M, Kini RM and Ribeiro JM. Sexual differences in the sialomes of the zebra tick, *Rhipicephalus pulchellus*. J Proteomics. 2015;117:120-44. doi:10.1016/j.jprot.2014.12.014.

123. Garcia GR, Gardinassi LG, Ribeiro JM, Anatriello E, Ferreira BR, Moreira HN, et al. The sialotranscriptome of *Amblyomma triste*, *Amblyomma parvum* and *Amblyomma cajennense* ticks, uncovered by 454-based RNA-seq. Parasit Vectors. 2014;7:430. doi:10.1186/1756-3305-7-430.

124. Jonckheere W, Dermauw W, Zhurov V, Wybouw N, Van den Bulcke J, Villarroel CA, et al. The salivary protein repertoire of the polyphagous spider mite *Tetranychus urticae*: a quest for effectors. Mol Cell Proteomics. 2016;15:3594-613. doi:10.1074/mcp.M116.058081.

125. Hunter DJ, Torkelson JL, Bodnar J, Mortazavi B, Laurent T, Deason J, et al. The *Rickettsia* endosymbiont of *Ixodes pacificus* contains all the genes of de novo folate biosynthesis. PLoS One. 2015;10:e0144552. doi:10.1371/journal.pone.0144552.

126. Rio RV, Attardo GM and Weiss BL. Grandeur Alliances: Symbiont metabolic integration and obligate arthropod hematophagy. Trends Parasitol. 2016;32:739-49. doi:10.1016/j.pt.2016.05.002.

127. Temeyer KB and Tuckow AP. Tick salivary cholinesterase: a probable immunomodulator of host-parasite interactions. J Med Entomol. 2016;53:500-4. doi:10.1093/jme/tjv252.

128. Junger WG. Immune cell regulation by autocrine purinergic signalling. Nat Rev Immunol. 2011;11:201-12. doi:10.1038/nri2938.

129. Pichu S, Yalcin EB, Ribeiro JM, King RS and Mather TN. Molecular characterization of novel sulfotransferases from the tick, *Ixodes scapularis*. BMC Biochem. 2011;12:32. doi:10.1186/1471-2091-12-32.

130. Thompson RE, Liu X, Ripoll-Rozada J, Alonso-Garcia N, Parker BL, Pereira PJB, et al. Tyrosine sulfation modulates activity of tick-derived thrombin inhibitors. Nat Chem. 2017;9:909-17. doi:10.1038/nchem.2744.

131. Blisnick AA, Foulon T and Bonnet SI. Serine protease inhibitors in ticks: an overview of their role in tick biology and tick-borne pathogen transmission. Front Cell Infect Microbiol. 2017;7:199. doi:10.3389/fcimb.2017.00199.

132. Fogaca AC, Almeida IC, Eberlin MN, Tanaka AS, Bulet P and Daffre S. Ixodidin, a novel antimicrobial peptide from the hemocytes of the cattle tick *Boophilus microplus* with inhibitory activity against serine proteinases. Peptides. 2006;27:667-74. doi:10.1016/j.peptides.2005.07.013.

133. Sasaki SD, de Lima CA, Lovato DV, Juliano MA, Torquato RJ and Tanaka AS. BmSI-7, a novel subtilisin inhibitor from *Boophilus microplus*, with activity toward Pr1 proteases from the fungus *Metarhizium anisopliae*. Exp Parasitol. 2008;118:214-20. doi:10.1016/j.exppara.2007.08.003.

134. Rapoport TA. Protein transport across the endoplasmic reticulum membrane: facts, models, mysteries. FASEB J. 1991;5:2792-8.

135. Bullard R, Allen P, Chao CC, Douglas J, Das P, Morgan SE, et al. Structural characterization of tick cement cones collected from in vivo and artificial membrane blood-fed Lone Star ticks (*Amblyomma americanum*). Ticks Tick Borne Dis. 2016;7:880-92. doi:10.1016/j.ttbdis.2016.04.006.

136. Kim TK, Curran J and Mulenga A. Dual silencing of long and short *Amblyomma americanum* acidic chitinase forms weakens the tick cement cone stability. J Exp Biol. 2014;217:3493-503. doi:10.1242/jeb.107979.

137. Schwager EE, Sharma PP, Clarke T, Leite DJ, Wierschin T, Pechmann M, et al. The house spider genome reveals an ancient whole-genome duplication during arachnid evolution. BMC Biol. 2017;15:62. doi:10.1186/s12915-017-0399-x.

138. Miller JR, Koren S, Dilley KA, Harkins DM, Stockwell TB, Shabman RS, et al. A draft genome sequence for the *Ixodes scapularis* cell line, ISE6. F1000Res. 2018;7:297. doi:10.12688/f1000research.13635.1.

139. Barrero RA, Guerrero FD, Black M, McCooke J, Chapman B, Schilkey F, et al. Gene-enriched draft genome of the cattle tick *Rhipicephalus microplus*: assembly by the hybrid Pacific Biosciences/Illumina approach enabled analysis of the highly repetitive genome. Int J Parasitol. 2017;47:569-83. doi:10.1016/j.ijpara.2017.03.007.

140. Burgess STG, Bartley K, Marr EJ, Wright HW, Weaver RJ, Prickett JC, et al. Draft genome assembly of the sheep scab mite, *Psoroptes ovis*. Genome Announc. 2018;6 doi:10.1128/genomeA.00265-18.

141. Barriere A, Yang SP, Pekarek E, Thomas CG, Haag ES and Ruvinsky I. Detecting heterozygosity in shotgun genome assemblies: Lessons from obligately outcrossing nematodes. Genome Res. 2009;19:470-80. doi:10.1101/gr.081851.108.

142. Navarro-Dominguez B, Ruiz-Ruano FJ, Camacho JPM, Cabrero J and Lopez-Leon MD. Transcription of a B chromosome CAP-G pseudogene does not influence normal Condensin Complex genes in a grasshopper. Sci Rep. 2017;7:17650. doi:10.1038/s41598-017-15894-5.

143. Sharma PP, Kaluziak ST, Perez-Porro AR, Gonzalez VL, Hormiga G, Wheeler WC, et al. Phylogenomic interrogation of Arachnida reveals systemic conflicts in phylogenetic signal. Mol Biol Evol. 2014;31:2963-84. doi:10.1093/molbev/msu235.

144. Rudkin DM, Young GA and Nowlan GS. The oldest horseshoe crab: A new xiphosurid from Late Ordovician Konservat-Lagerstatten deposits, Manitoba, Canada. Palaeontology. 2008;51:1-9. doi:10.1111/j.1475-4983.2007.00746.x.

145. Dunlop JA. Geological history and phylogeny of Chelicerata. Arthropod Struct Dev. 2010;39:124-42. doi:10.1016/j.asd.2010.01.003.

146. Dabert M, Witalinski W, Kazmierski A, Olszanowski Z and Dabert J. Molecular phylogeny of acariform mites (Acari, Arachnida): strong conflict between phylogenetic signal and long-branch attraction artifacts. Mol Phylogenet Evol. 2010;56:222-41. doi:10.1016/j.ympev.2009.12.020.

147. Niedzwiedzki G, Szrek P, Narkiewicz K, Narkiewicz M and Ahlberg PE. Tetrapod trackways from the early Middle Devonian period of Poland. Nature. 2010;463:43-8. doi:10.1038/nature08623.

148. Konikiewicz M and Makol J. A fossil Paratrombiinae mite (Actinotrichida: Trombidioidea) from the Rovno amber, Ukraine. Zootaxa. 2014;3847:583-9. doi:10.11646/zootaxa.3847.4.8.

149. Karahadian C, Josephson DB and Lindsay RC. Volatile Compounds from *Penicillium* sp contributing musty earthy notes to Brie and Camembert cheese flavors. J Agr Food Chem. 1985;33:339-43.

150. Gerber NN. Volatile substances from actinomycetes: their role in the odor pollution of water. CRC Crit Rev Microbiol. 1979;7:191-214.

151. Gerber NN and Lechevalier HA. Geosmin, an earthly-smelling substance isolated from actinomycetes. Appl Microbiol. 1965;13:935-8.

152. Tabachek JAL and Yurkowski M. Isolation and identification of blue-green-algae producing muddy odor metabolites, geosmin, and 2-methylisoborneol, in saline lakes in Manitoba. J Fish Res Board Can. 1976;33:25-35.

153. Stensmyr MC, Dweck HK, Farhan A, Ibba I, Strutz A, Mukunda L, et al. A conserved dedicated olfactory circuit for detecting harmful microbes in *Drosophila*. Cell. 2012;151:1345-57. doi:10.1016/j.cell.2012.09.046.

154. Ashitani T, Garboui SS, Schubert F, Vongsombath C, Liblikas I, Palsson K, et al. Activity studies of sesquiterpene oxides and sulfides from the plant *Hyptis suaveolens* (Lamiaceae) and its repellency on *Ixodes ricinus* (Acari: Ixodidae). Exp Appl Acarol. 2015;67:595-606. doi:10.1007/s10493-015-9965-5.

155. Birkett MA, Abassi SA, Krober T, Chamberlain K, Hooper AM, Guerin PM, et al. Antiectoparasitic activity of the gum resin, gum haggar, from the East African plant, *Commiphora holtziana*. Phytochemistry. 2008;69:1710-5. doi:10.1016/j.phytochem.2008.02.017.

156. Baldin ELL, Souza ES, Silva JPGF, Pavarini DP, Lopes NP, Lopes JLC, et al. Towards new botanical pesticides: the toxic effect of *Eremanthus goyazensis* (Asteraceae) leaves essential oil against *Brevipalpus phoenicis* (Acari: Tenuipalpidae). Quim Nova. 2012;35:2254-7.

157. Huang RL, Li ZH, Wang SY, Fu JT, Cheng DM and Zhang ZX. Insecticidal effect of volatile compounds from plant materials of *Murraya exotica* against Red Imported Fire Ant Workers. Sociobiology. 2016;63:783-91.

158. Cloudsley-Thompson JL. Some aspects of the physiology and behaviour of *Dinothrombium* (Acari). Entomologia Experimentalis et Applicata. 1962;5:69-73.

159. Sferra NJ. 1st Record of *Pterodontia flavipes* (Diptera, Acroceridae) larvae in the mites *Podothrombium* (Acari, Trombidiidae) and *Abrolophus* (Acari, Erythraeidae). Entomol News. 1986;97:121-3.

160. Ozawa R, Shimoda T, Kawaguchi M, Arimura G, Horiuchi J, Nishioka T, et al. *Lotus japonicus* infested with herbivorous mites emits volatile compounds that attract predatory mites. J Plant Res. 2000;113:427-33.

161. Royalty RN, Phelan PL and Hall FR. Arrestment of male 2-spotted spider-mite caused by female sex-pheromone. J Chem Ecol. 1992;18:137-53.

162. Wohltmann A. The evolution of life histories in Parasitengona (Acari: Prostigmata). Acarologia. 2000;41:145-204.

163. Moniuszko H and Makol J. Host-parasite association in trombiculid mites (Actinotrichida: Trombiculidae) of temperate zone - the case of *Hirsutiella zachvatkini* (Schluger, 1948); are we dealing with prolonged contact with the host? Parasit Vectors. 2016;9:61. doi:10.1186/s13071-016-1339-2.

164. Khan AA and Quigley JG. Heme and FLVCR-related transporter families SLC48 and SLC49. Mol Aspects Med. 2013;34:669-82. doi:10.1016/j.mam.2012.07.013.

165. Mandilaras K and Missirlis F. Genes for iron metabolism influence circadian rhythms in *Drosophila melanogaster*. Metallomics. 2012;4:928-36. doi:10.1039/c2mt20065a.

166. Bryon A, Kurlovs AH, Van Leeuwen T and Clark RM. A molecular-genetic understanding of diapause in spider mites: current knowledge and future directions. Physiol Entomol. 2017;42:211-24.

167. Royalty RN, Phelan PL and Hall FR. Comparative effects of form, color, and pheromone of 2-spotted spider-mite quiescent deutonymphs on male guarding behavior. Physiol Entomol. 1993;18:303-16.

168. Goto SG. Physiological and molecular mechanisms underlying photoperiodism in the spider mite: comparisons with insects. J Comp Physiol B. 2016;186:969-84. doi:10.1007/s00360-016-1018-9.

169. Hori Y, Numata H, Shiga S and Goto SG. Both the anterior and posterior eyes function as photoreceptors for photoperiodic termination of diapause in the two-spotted spider mite. J Comp Physiol A Neuroethol Sens Neural Behav Physiol. 2014;200:161-7. doi:10.1007/s00359-013-0872-0.

170. Terakita A and Nagata T. Functional properties of opsins and their contribution to light-sensing physiology. Zoolog Sci. 2014;31:653-9. doi:10.2108/zs140094.

171. Ni JD, Baik LS, Holmes TC and Montell C. A rhodopsin in the brain functions in circadian photoentrainment in *Drosophila*. Nature. 2017;545:340-4. doi:10.1038/nature22325.

172. Tanji T, Ohashi-Kobayashi A and Natori S. Participation of a galactose-specific C-type lectin in *Drosophila* immunity. Biochem J. 2006;396:127-38. doi:10.1042/BJ20051921.

173. Costa FH, Valenca NS, Silva AR, Bezerra GA, Cavada BS and Radis-Baptista G. Cloning and molecular modeling of *Litopenaeus vannamei* (Penaeidae) C-type lectin homologs with mutated mannose binding domain-2. Genet Mol Res. 2011;10:650-64. doi:10.4238/vol10-2gmr999.

174. Walsh AM, Kortschak RD, Gardner MG, Bertozzi T and Adelson DL. Widespread horizontal transfer of retrotransposons. Proc Natl Acad Sci U S A. 2013;110:1012-6. doi:10.1073/pnas.1205856110.

175. Santos-Matos G, Wybouw N, Martins NE, Zele F, Riga M, Leitao AB, et al. *Tetranychus urticae* mites do not mount an induced immune response against bacteria. Proc Biol Sci. 2017;284 doi:10.1098/rspb.2017.0401.

176. Yang L and Zhu R. Immunotherapy of house dust mite allergy. Hum Vaccin Immunother. 2017;13:2390-6. doi:10.1080/21645515.2017.1364823.

177. de la Fuente J and Contreras M. Tick vaccines: current status and future directions. Expert Rev Vaccines. 2015;14:1367-76. doi:10.1586/14760584.2015.1076339.

178. Burgess ST, Nunn F, Nath M, Frew D, Wells B, Marr EJ, et al. A recombinant subunit vaccine for the control of ovine psoroptic mange (sheep scab). Vet Res. 2016;47:26. doi:10.1186/s13567-016-0315-3.

179. Bartley K, Wright HW, Huntley JF, Manson ED, Inglis NF, McLean K, et al. Identification and evaluation of vaccine candidate antigens from the poultry red mite (*Dermanyssus gallinae*). Int J Parasitol. 2015;45:819-30. doi:10.1016/j.ijpara.2015.07.004.

180. Wongprompitak P, Duong V, Anukool W, Sreyrath L, Mai TT, Gavotte L, et al. *Orientia tsutsugamushi*, agent of scrub typhus, displays a single metapopulation with maintenance of ancestral haplotypes throughout continental South East Asia. Infect Genet Evol. 2015;31:1-8. doi:10.1016/j.meegid.2015.01.005.

181. Auffray JC, Blasdell, K.R., Bordes, F., Chabé, M., Chaisiri, K., Charbonnel, N., Claude, J., Dei-Cas, E., Desquesnes, M., Dobigny, G., Douangboupha, B., Galan, M., Haukisalmi, V., Henttonen, H., Hugot, J.P., Jiyipong, T., Latinne, A., Michaux, J., Milocco, C., Morand, S., Pagès, M., Phoophitpong, D.,

Pumhom, P., Ribas Salvador, A., Soonchan, S., Suputtamongkol, Y., Waengsothorn, S., Waywa, D., Xuereb, A. Protocols for field and laboratory rodent studies. Bangkok: Kasetsart University Press; 2011.

182. Martin M. Cutadapt removes adapter sequences from high-throughput sequencing reads. EMBnetjournal. 2011;17:10-2.

183. Joshi NA, Fass, J.N.: Sickle: A sliding-window, adaptive, quality-based trimming tool for FastQ files. https://github.com/najoshi/sickle (2011). Accessed 24/01/18.

184. Wood DE and Salzberg SL. Kraken: ultrafast metagenomic sequence classification using exact alignments. Genome Biol. 2014;15:R46. doi:10.1186/gb-2014-15-3-r46.

185. Marcais G and Kingsford C. A fast, lock-free approach for efficient parallel counting of occurrences of k-mers. Bioinformatics. 2011;27:764-70. doi:10.1093/bioinformatics/btr011.

186. Vurture GW, Sedlazeck FJ, Nattestad M, Underwood CJ, Fang H, Gurtowski J, et al. GenomeScope: fast reference-free genome profiling from short reads. Bioinformatics. 2017;33:2202-4. doi:10.1093/bioinformatics/btx153.

187. Gnerre S, Maccallum I, Przybylski D, Ribeiro FJ, Burton JN, Walker BJ, et al. High-quality draft assemblies of mammalian genomes from massively parallel sequence data. Proc Natl Acad Sci U S A. 2011;108:1513-8. doi:10.1073/pnas.1017351108.

188. Luo R, Liu B, Xie Y, Li Z, Huang W, Yuan J, et al. SOAPdenovo2: an empirically improved memory-efficient short-read de novo assembler. Gigascience. 2012;1:18. doi:10.1186/2047-217X-1-18.

189. Weisenfeld NI, Yin S, Sharpe T, Lau B, Hegarty R, Holmes L, et al. Comprehensive variation discovery in single human genomes. Nat Genet. 2014;46:1350-5. doi:10.1038/ng.3121.

190. Langmead B and Salzberg SL. Fast gapped-read alignment with Bowtie 2. Nat Methods. 2012;9:357-9. doi:10.1038/nmeth.1923.

191. Parra G, Bradnam K and Korf I. CEGMA: a pipeline to accurately annotate core genes in eukaryotic genomes. Bioinformatics. 2007;23:1061.

192. Smit A, Hubley, R.: RepeatModeler: De-novo repeat discovery tool. https://github.com/rmhubley/RepeatModeler (2017). Accessed 24/01/18.

193. Tarailo-Graovac M and Chen N. Using RepeatMasker to identify repetitive elements in genomic sequences. Curr Protoc Bioinformatics. 2009;Chapter 4:Unit 4 10. doi:10.1002/0471250953.bi0410s25.

194. Zdobnov EM and Apweiler R. InterProScan--an integration platform for the signature-recognition methods in InterPro. Bioinformatics. 2001;17:847-8.

195. Conesa A, Gotz S, Garcia-Gomez JM, Terol J, Talon M and Robles M. Blast2GO: a universal tool for annotation, visualization and analysis in functional genomics research. Bioinformatics. 2005;21:3674-6. doi:10.1093/bioinformatics/bti610.

196. Kanehisa M and Goto S. KEGG: Kyoto Encyclopedia of Genes and Genomes. Nucleic Acids Research. 2000;27:29-34(6).

197. Adams M, Celniker S, Holt R, Evans C, Gocayne J, Amanatides P, et al. The genome sequence of *Drosophila melanogaster*. Science. 2000;287:2185-95. doi:10.1126/science.287.5461.2185.

198. Weinstock G, Robinson G, Gibbs R, Worley K, Evans J, Maleszka R, et al. Insights into social insects from the genome of the honeybee *Apis mellifera*. Nature. 2006;443:931-49. doi:10.1038/nature05260.

199. Gulia-Nuss M, Nuss AB, Meyer JM, Sonenshine DE, Roe RM, Waterhouse RM, et al. Genomic insights into the *Ixodes scapularis* tick vector of Lyme disease. Nat Commun. 2016;7:10507. doi:10.1038/ncomms10507.

200. Battelle BA, Ryan JF, Kempler KE, Saraf SR, Marten CE, Warren WC, et al. Opsin repertoire and expression patterns in horseshoe crabs: evidence from the genome of *Limulus polyphemus* (Arthropoda: Chelicerata). Genome Biol Evol. 2016;8:1571-89. doi:10.1093/gbe/evw100.

201. Stein L, Sternberg P, Durbin R, Thierry-Mieg J and Spieth J. WormBase: network access to the genome and biology of *Caenorhabditis elegans*. Nucleic Acids Research. 2001;29:82-6. doi:10.1093/nar/29.1.82.

202. Katoh K and Standley DM. MAFFT multiple sequence alignment software version 7: improvements in performance and usability. Mol Biol Evol. 2013;30:772-80. doi:10.1093/molbev/mst010.

203. Lassmann T, Frings O and Sonnhammer EL. Kalign2: high-performance multiple alignment of protein and nucleotide sequences allowing external features. Nucleic Acids Res. 2009;37:858-65. doi:10.1093/nar/gkn1006.

204. Darriba D, Taboada GL, Doallo R and Posada D. ProtTest 3: fast selection of best-fit models of protein evolution. Bioinformatics. 2011;27:1164-5. doi:10.1093/bioinformatics/btr088.

205. Guindon S, Dufayard JF, Lefort V, Anisimova M, Hordijk W and Gascuel O. New algorithms and methods to estimate maximum-likelihood phylogenies: assessing the performance of PhyML 3.0. Syst Biol. 2010;59:307-21. doi:10.1093/sysbio/syq010.

206. Kumar S, Stecher G and Tamura K. MEGA7: Molecular Evolutionary Genetics Analysis version 7.0 for bigger datasets. Molecular Biology & Evolution. 2016;33:1870.

207. Castresana J. Selection of conserved blocks from multiple alignments for their use in phylogenetic analysis. Mol Biol Evol. 2000;17:540-52.

208. Yang Z. PAML 4: phylogenetic analysis by maximum likelihood. Mol Biol Evol. 2007;24:1586-91. doi:10.1093/molbev/msm088.

209. Benton MJ, Donoghue PCJ, Asher RJ, Friedman M, Near TJ and Vinther J. Constraints on the timescale of animal evolutionary history. Palaeontol Electron. 2015;18.

210. Benton M, Donoghue, PCJ, Asher, RJ. Calibrating and constraining molecular clocks. In: Hedges SB, Kumar, S., editor. The timetree of life. Oxford: Oxford University Press; 2009. p. 35 - 86.

211. Li L, Stoeckert CJ and Roos DS. OrthoMCL: identification of ortholog groups for eukaryotic genomes. Genome Res. 2003;13:2178-89. doi:10.1101/gr.1224503.

212. De Bie T, Cristianini N, Demuth JP and Hahn MW. CAFE: a computational tool for the study of gene family evolution. Bioinformatics. 2006;22:1269-71. doi:10.1093/bioinformatics/btl097.

213. Bendtsen JD, Jensen LJ, Blom N, Von Heijne G and Brunak S. Feature-based prediction of non-classical and leaderless protein secretion. Protein Eng Des Sel. 2004;17:349-56. doi:10.1093/protein/gzh037.

214. Krogh A, Larsson B, von Heijne G and Sonnhammer EL. Predicting transmembrane protein topology with a hidden Markov model: application to complete genomes. J Mol Biol. 2001;305:567-80. doi:10.1006/jmbi.2000.4315.

215. Gupta R, Jung, E., Brunak, S.: NetNGlyc 1.0 server. http://www.cbs.dtu.dk/services/NetNGlyc/ (2004). Accessed 25/01/18.

216. Finn RD, Attwood TK, Babbitt PC, Bateman A, Bork P, Bridge AJ, et al. InterPro in 2017-beyond protein family and domain annotations. Nucleic Acids Res. 2017;45:D190-D9. doi:10.1093/nar/gkw1107.

217. Sánchez-Gracia A, Vieira FG, Almeida FC and Rozas J. Comparative Genomics of the Major Chemosensory Gene Families in Arthropods. John Wiley & Sons, Ltd; 2011.

218. Nozawa M and Nei M. Evolutionary dynamics of olfactory receptor genes in *Drosophila* species. Proc Natl Acad Sci U S A. 2007;104:7122.

219. Robertson HM and Wanner KW. The chemoreceptor superfamily in the honey bee, *Apis mellifera*: Expansion of the odorant, but not gustatory, receptor family. Genome Research. 2006;16:1395.

220. Robertson HM, Warr CG and Carlson JR. Molecular evolution of the insect chemoreceptor gene superfamily in *Drosophila melanogaster*. Proc Natl Acad Sci U S A. 2003;100 Suppl 2:14537.

221. Croset V, Rytz R, Cummins SF, Budd A, Brawand D, Kaessmann H, et al. Ancient protostome origin of chemosensory ionotropic glutamate receptors and the evolution of insect taste and olfaction. PLoS Genetics. 2010;6:e1001064.

222. Dang HX and Lawrence CB. Allerdictor: fast allergen prediction using text classification techniques. Bioinformatics. 2014;30:1120-8. doi:10.1093/bioinformatics/btu004.

223. Grabherr MG, Haas BJ, Yassour M, Levin JZ, Thompson DA, Amit I, et al. Full-length transcriptome assembly from RNA-Seq data without a reference genome. Nat Biotechnol. 2011;29:644-52. doi:10.1038/nbt.1883.

224. Finn RD, Bateman A, Clements J, Coggill P, Eberhardt RY, Eddy SR, et al. Pfam: the protein families database. Nucleic Acids Res. 2014;42:D222-30. doi:10.1093/nar/gkt1223.

225. Heberle H, Meirelles GV, da Silva FR, Telles GP and Minghim R. InteractiVenn: a web-based tool for the analysis of sets through Venn diagrams. BMC Bioinformatics. 2015;16:169. doi:10.1186/s12859-015-0611-3.

226. Park SW, Ha NY, Ryu B, Bang JH, Song H, Kim Y, et al. Urbanization of scrub typhus disease in South Korea. PLoS Negl Trop Dis. 2015;9:e0003814. doi:10.1371/journal.pntd.0003814.

227. Ternent T, Csordas A, Qi D, Gomez-Baena G, Beynon RJ, Jones AR, et al. How to submit MS proteomics data to ProteomeXchange via the PRIDE database. Proteomics. 2014;14:2233-41. doi:10.1002/pmic.201400120.

228. Armstrong SD, Babayan SA, Lhermitte-Vallarino N, Gray N, Xia D, Martin C, et al. Comparative analysis of the secretome from a model filarial nematode (*Litomosoides sigmodontis*) reveals maximal diversity in gravid female parasites. Mol Cell Proteomics. 2014;13:2527-44. doi:10.1074/mcp.M114.038539.

229. Benjamini Y and Hochberg Y. Controlling the false discovery rate - a practical and powerful approach to multiple testing. J Roy Stat Soc B Met. 1995;57:289-300.

230. Dong X, Chaisiri K, Xia D, Armstrong SD, Fang Y, Donnelly MJ, et al. Supporting data for "Genomes of trombidid mites reveal novel predicted allergens and laterally transferred genes associated with secondary metabolism". GigaScience Database. 2018. doi:http://dx.doi.org/10.5524/100505.

231. Golab GC, Patterson-Kane, E. AVMA Guidelines for the Euthanasia of Animals (2013 Edition). 2013.0.1 ed. Schaumburg, Illinois: American Veterinary Medical Association; 2013.

232. Charbonneau R, Niel, L., Olfert, E., von Keyserlingk, M., Griffin, G. CCAC guidelines on: euthanasia of animals used in science. Ottawa: Canadian Council on Animal Care; 2010.

233. Marchler-Bauer A, Bo Y, Han L, He J, Lanczycki CJ, Lu S, et al. CDD/SPARCLE: functional classification of proteins via subfamily domain architectures. Nucleic Acids Res. 2017;45:D200-D3. doi:10.1093/nar/gkw1129.

Figure 1

Click here to access/download;Figu

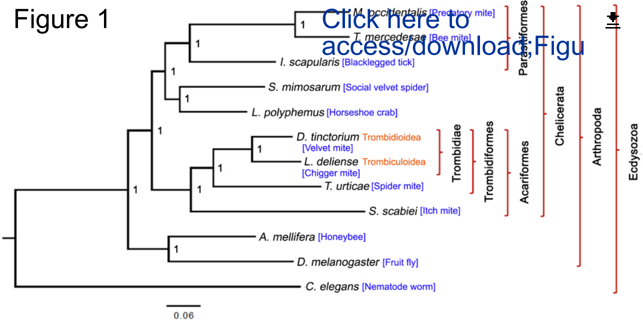

Figure 2

Click here to access/download;Figure;Figure_2.pdf

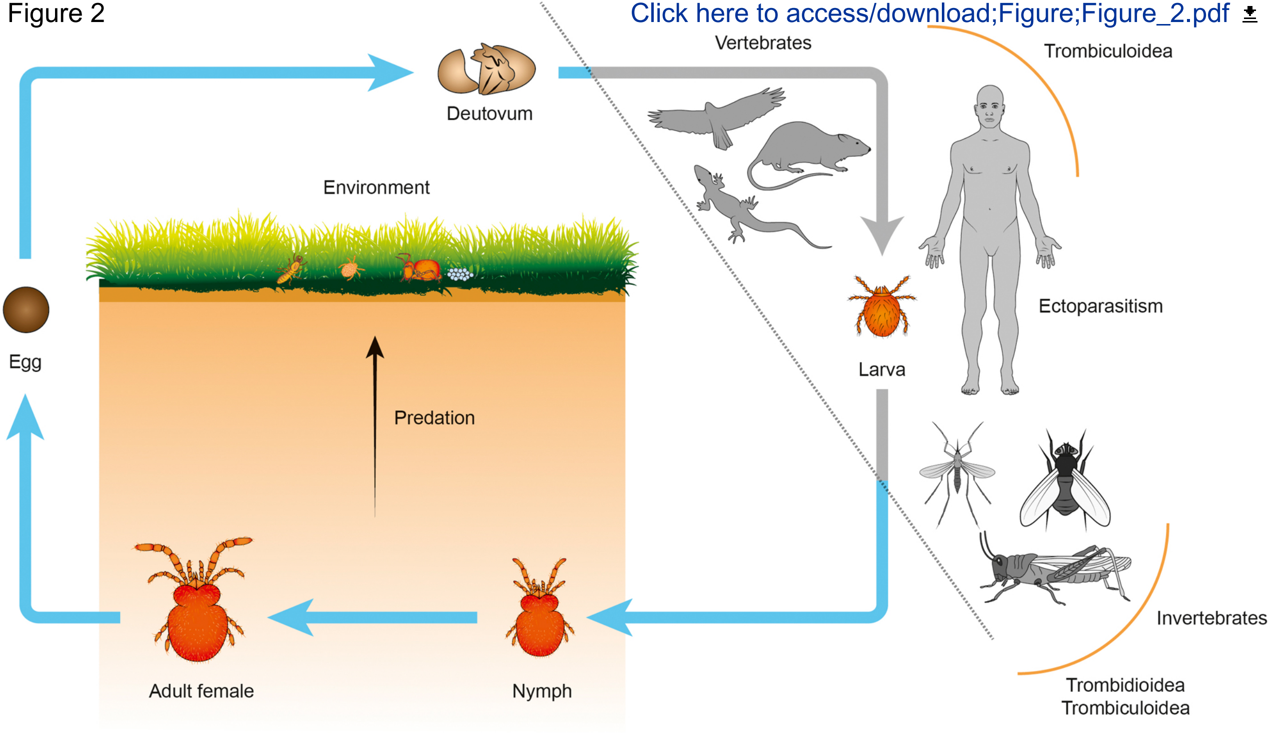

Figure 3

Click here to access/download;Figu

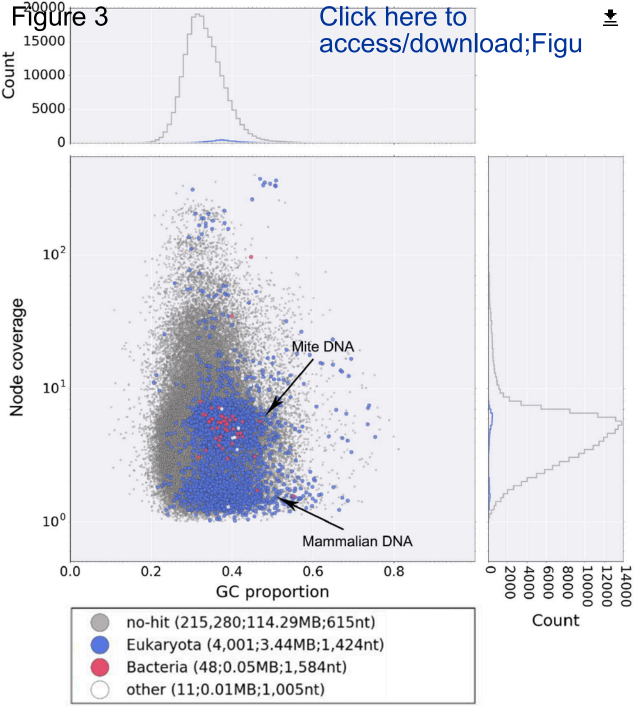

Figure 4

Click here to access/download;Figure;Figure_4.pdf

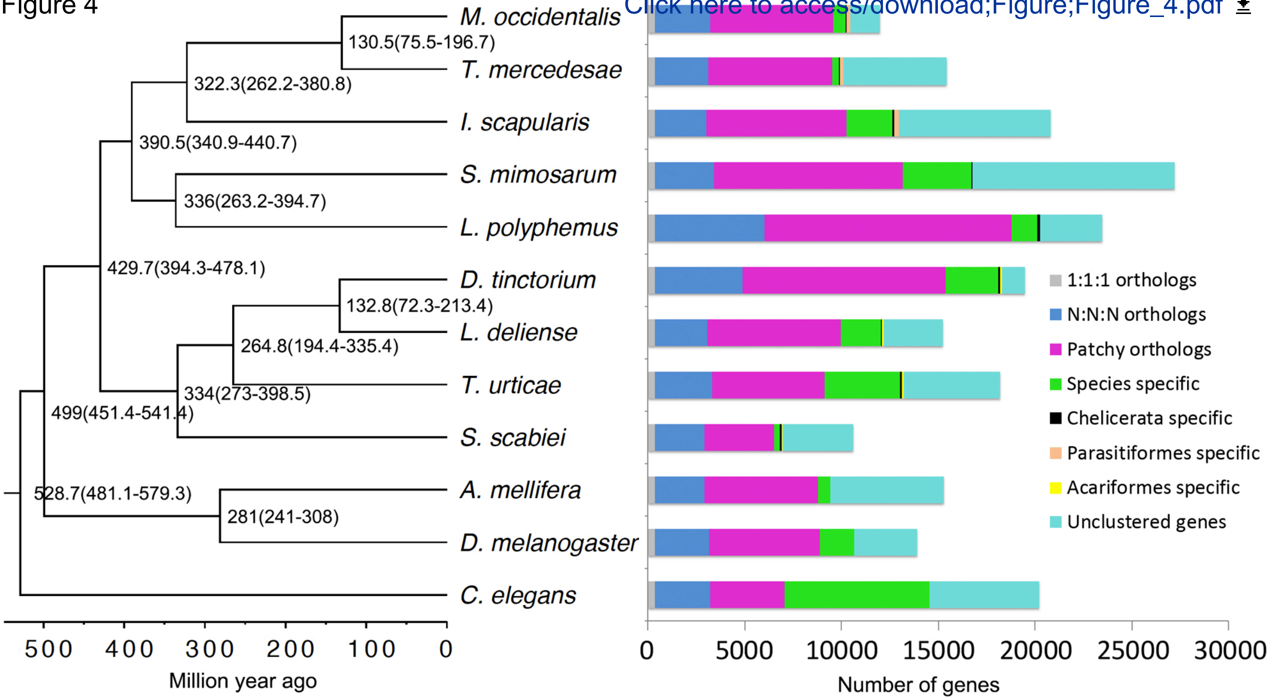

Figure 5

Click here to access/download;Figu

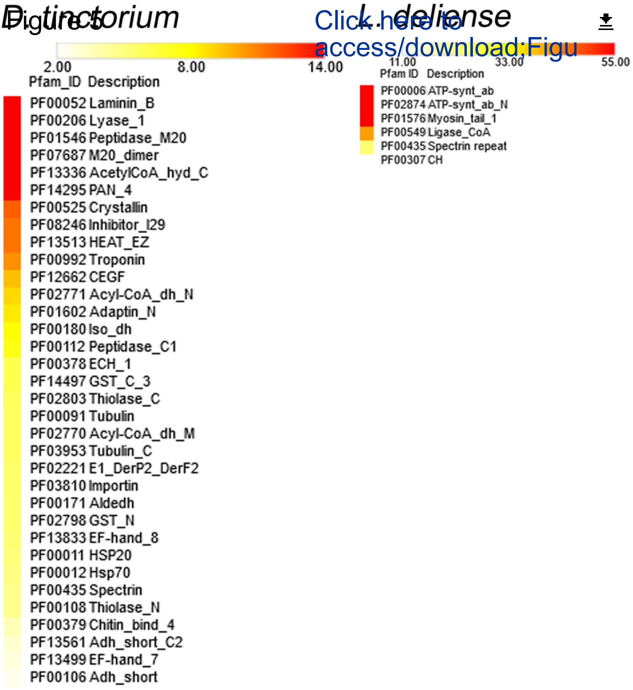

Figure 6

Click here to access/download;Figure;Figure_6.pdf

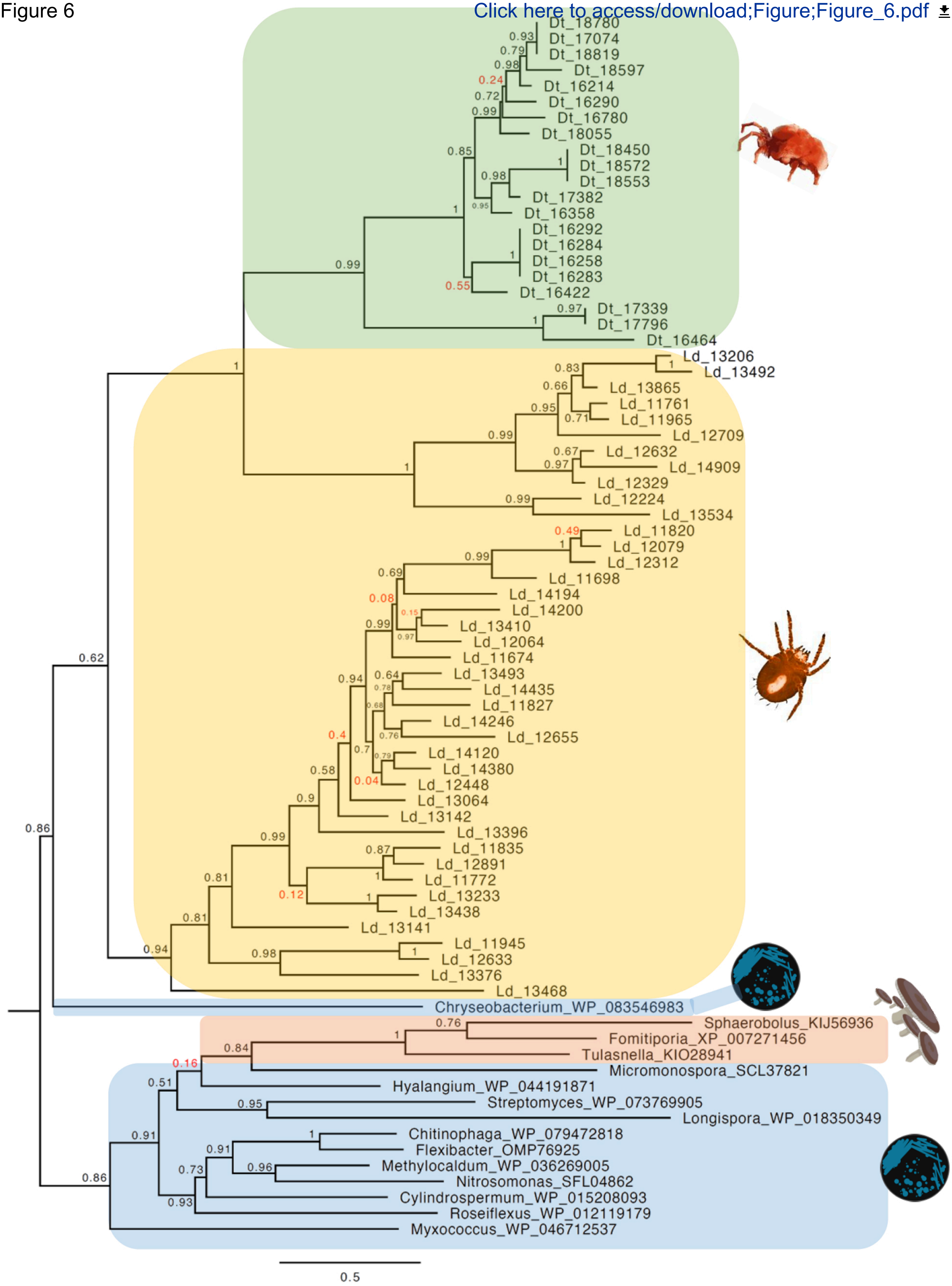

Figure 7

Click here to access/download;Figure;Figure_7.pdf

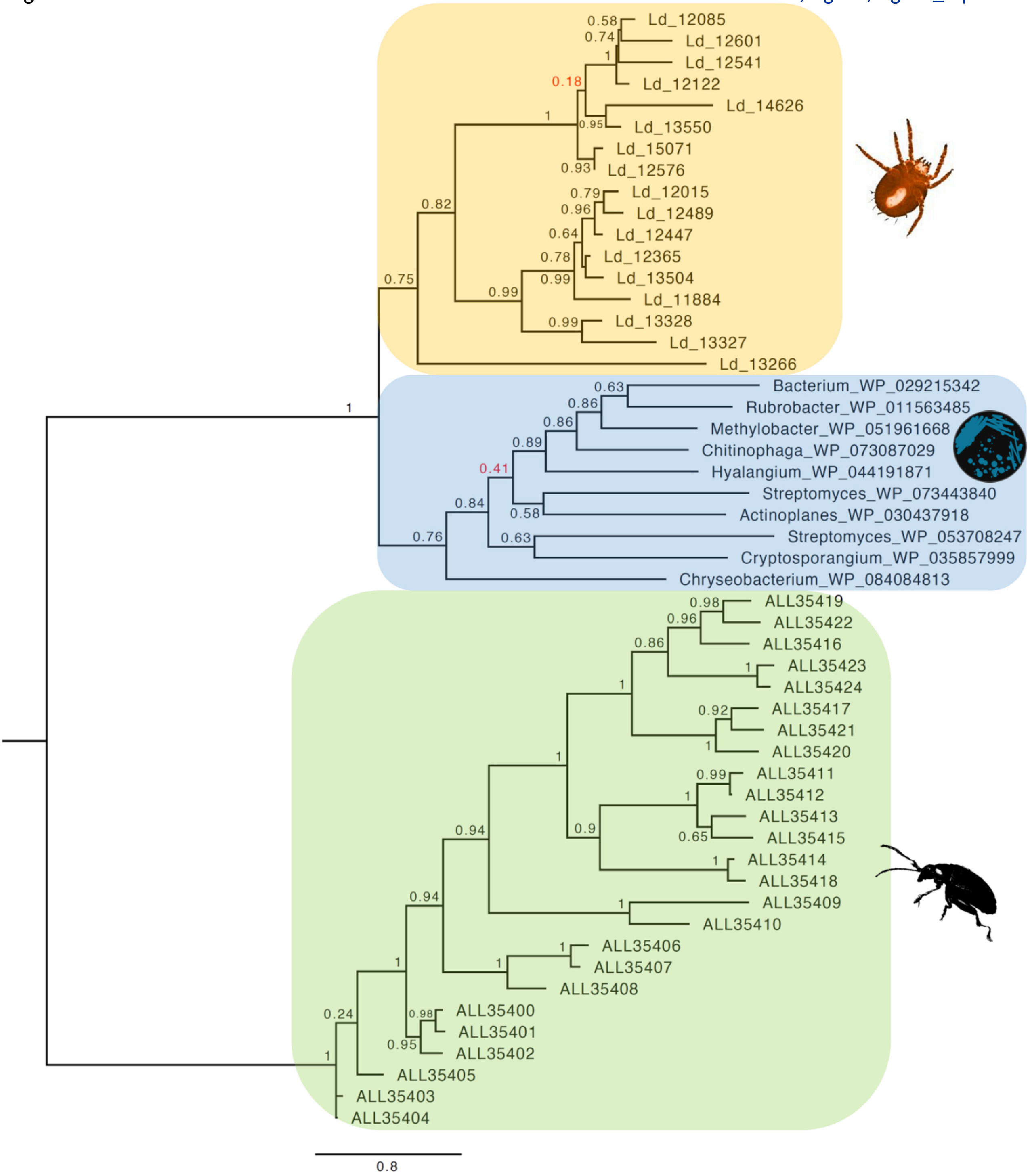

Figure 8

Click here to access/download;Figure;Figure_8.pdf

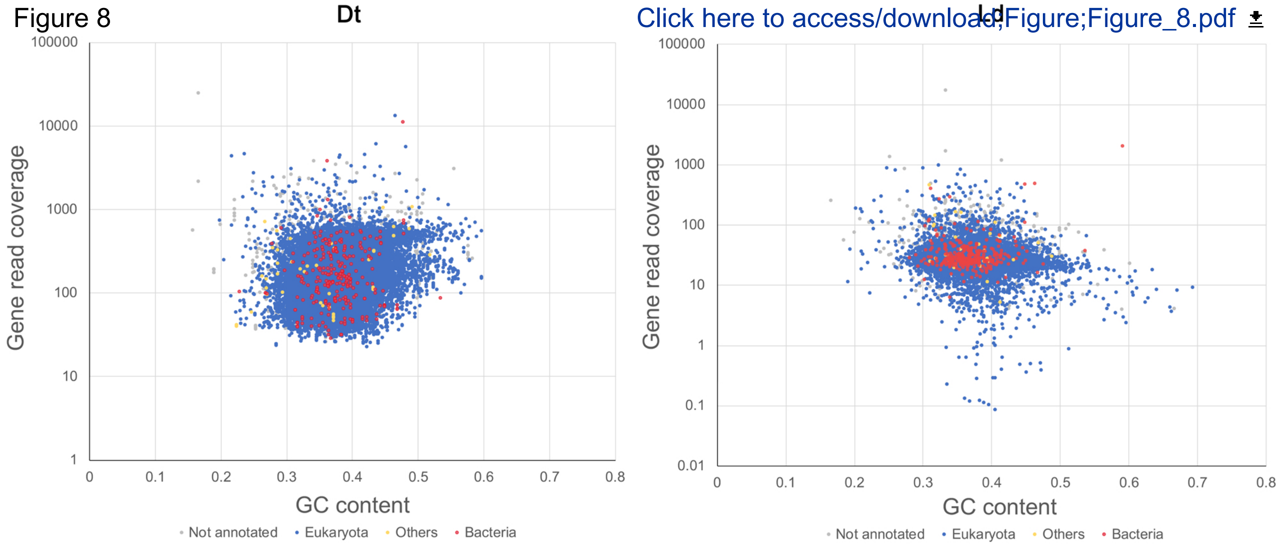

Figure 9

Click here to access/download;Figure;Figure_9.pdf

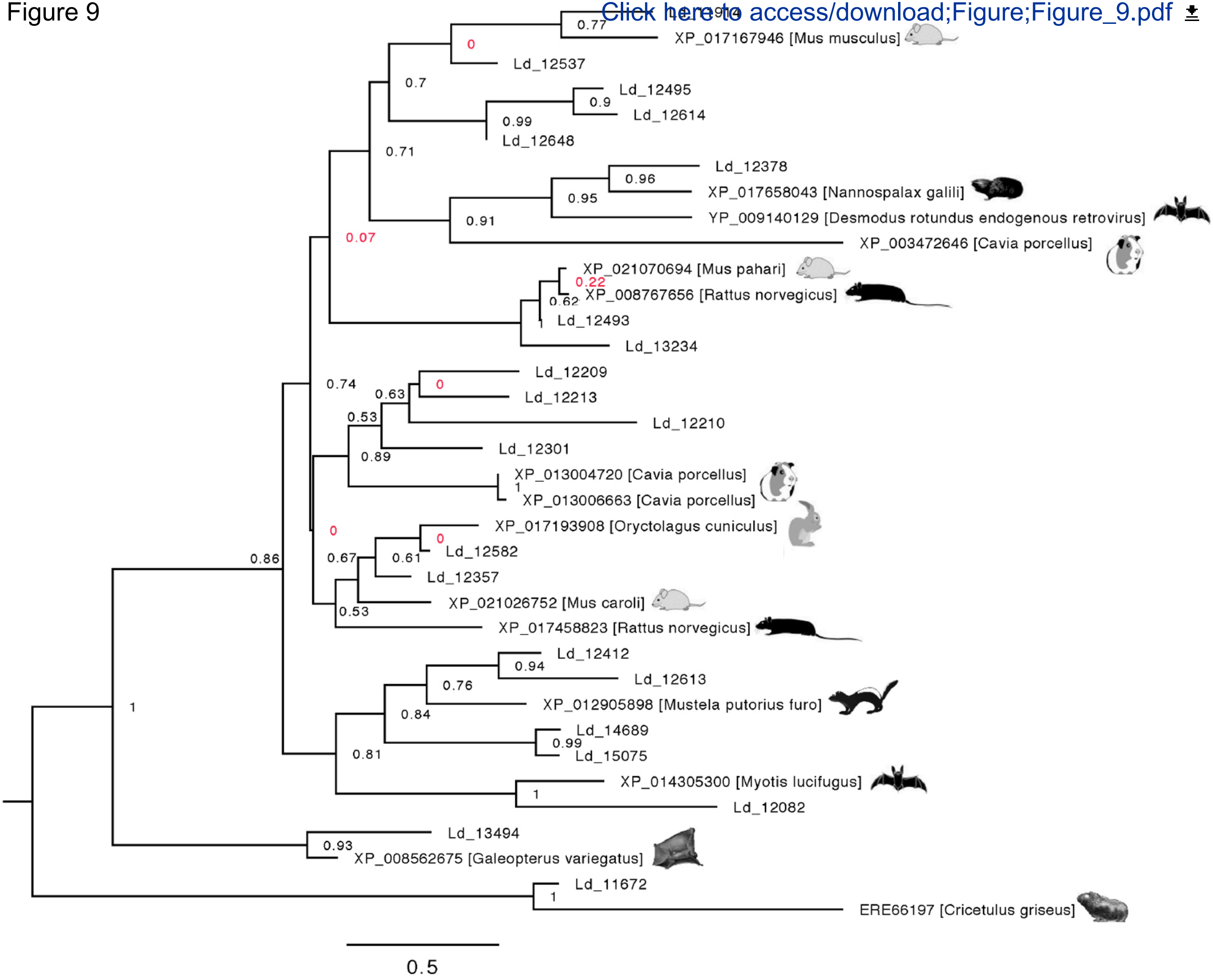

Figure 10

Click here to access/download;Figu

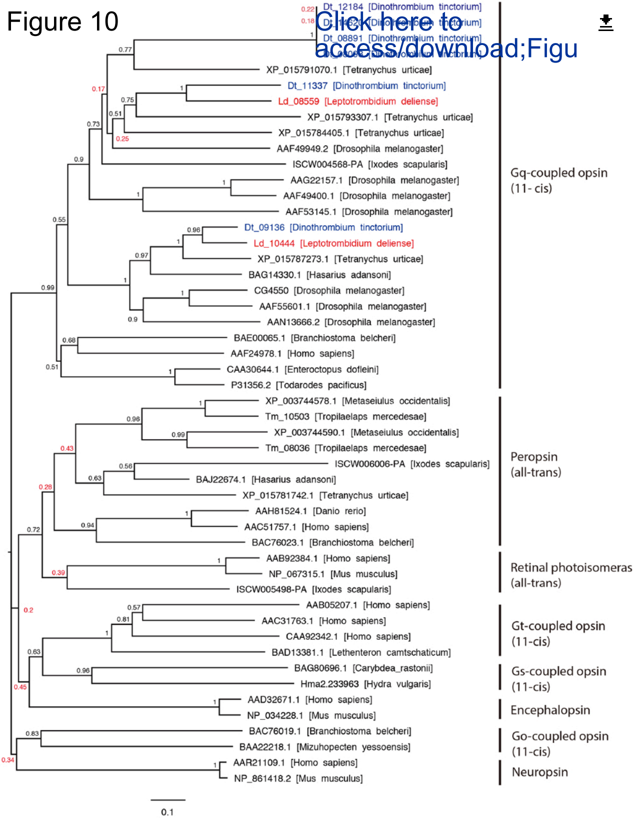

Figure 11

Click here to access/download;Figure;Figure_11.pdf

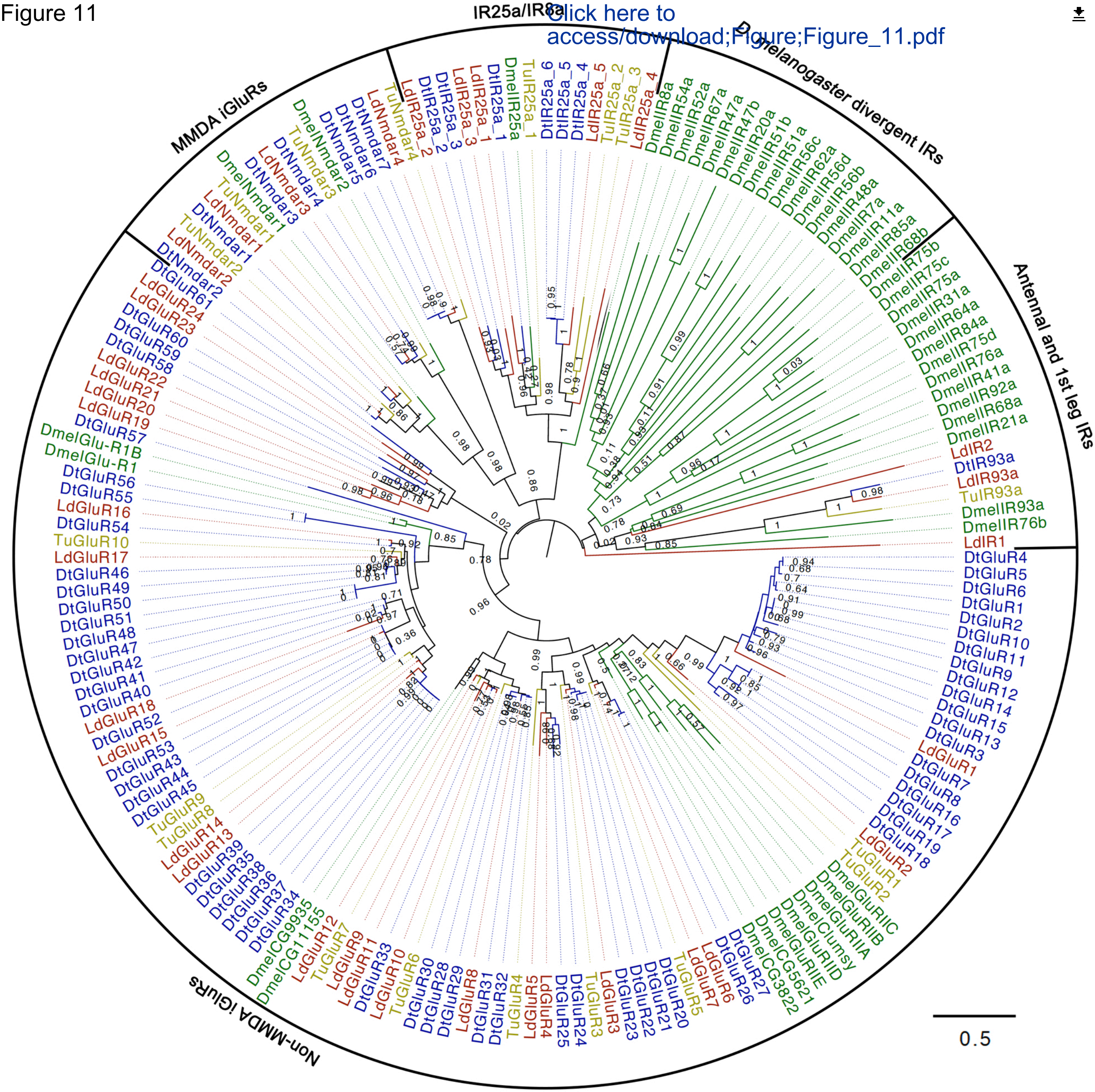

Figure 12

Click here to access/download;Figure;Figure_12.pdf

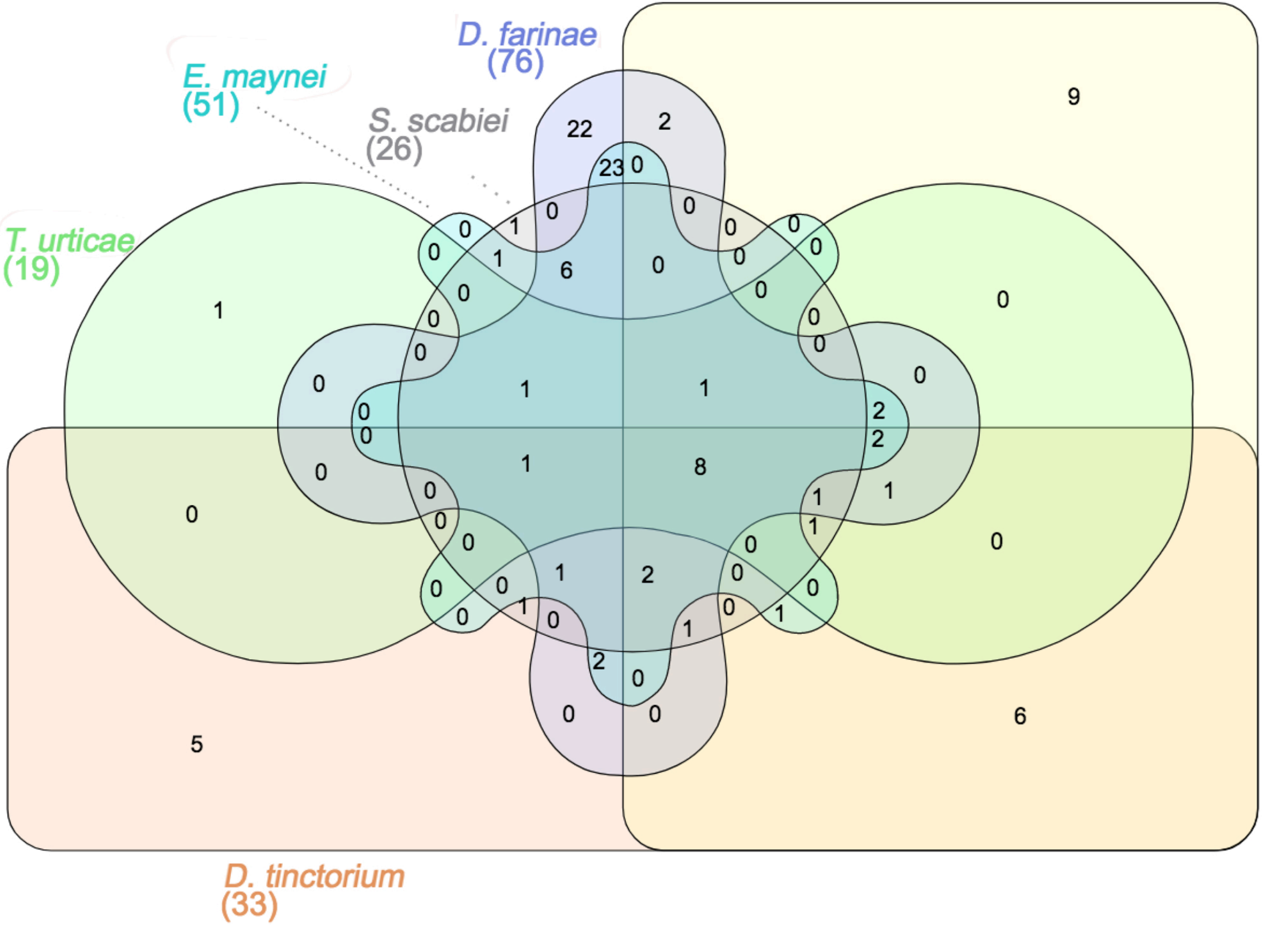

Figure 13

Click here to access/download;Figure;Figure_13.pdf

L. deliense gene ID	Orthlogous cluster	AllFam	Description	Peptides
Ld_01656	ORTHOMCL2	AF100	Myosin heavy chain	35
Ld_08426	ORTHOMCL25	AF007	EF hand family	4
Ld_09890	ORTHOMCL29	AF024	Trypsin-like serine protease	0
Ld_02714	ORTHOMCL12	AF021	Subtilase family	0
Ld_03075	ORTHOMCL12	AF021	Subtilase family	0
Ld_07213	ORTHOMCL12	AF021	Subtilase family	0
Ld_08430	ORTHOMCL12	AF021	Subtilase family	0
Ld_09243	ORTHOMCL12	AF021	Subtilase family	0
Ld_09299	ORTHOMCL12	AF021	Subtilase family	0
Ld_14039	ORTHOMCL12	AF021	Subtilase family	0
Ld_00621	ORTHOMCL10	AF054	Tropomyosin	10
Ld_11355	ORTHOMCL10	AF054	Tropomyosin	13
Ld_00109	ORTHOMCL11	AF038	Cyclophilin	1
Ld_10361	ORTHOMCL20	AF197	Fructose-bisphosphate aldolase	4
Ld_01930	ORTHOMCL1	AF025	Tubulin/FtsZ family	0
Ld_07500	ORTHOMCL16	-	Nucleoside diphosphate kinase	4
Ld_07791	ORTHOMCL17	AF030	Papain-like cysteine protease	0
Ld_00033	ORTHOMCL18	AF030	Papain-like cysteine protease	5
Ld_08227	ORTHOMCL18	AF030	Papain family cysteine protease	0
Ld_08323	ORTHOMCL19	AF020	Superoxide dismutase	0
Ld_02800	ORTHOMCL31	AF024	Trypsin-like serine protease	0
Ld_09622	ORTHOMCL31	AF024	Trypsin-like serine protease	0
Ld_09623	ORTHOMCL31	AF024	Trypsin-like serine protease	0
Ld_10525	ORTHOMCL31	AF024	Trypsin-like serine protease	0
Ld_11514	ORTHOMCL31	AF024	Trypsin-like serine protease	0
Ld_06375	ORTHOMCL3	-	Casein kinase II subunit beta	0
Ld_01868	ORTHOMCL32	AF024	Trypsin-like serine protease	0
Ld_02046	ORTHOMCL32	AF024	Trypsin-like serine protease	0
Ld_06856	ORTHOMCL32	AF024	Trypsin-like serine protease	0
Ld_07293	ORTHOMCL32	AF024	Trypsin-like serine protease	0
Ld_10686	ORTHOMCL32	AF024	Trypsin-like serine protease	0
Ld_03432	ORTHOMCL23	AF032	Triosephosphate isomerase	3
Ld_08478	ORTHOMCL33	AF142	WD domain, G-beta repeat	9
Ld_08833	ORTHOMCL30	AF024	Trypsin-like serine protease	0
Ld_02654	ORTHOMCL39	AF030	Papain-like cysteine protease	0
Ld_04617	ORTHOMCL4	AF031	Enolase and Methylaspartate ammonia-lyase	1
Ld_09789	ORTHOMCL43	AF024	Trypsin-like serine protease	0
Ld_04990	ORTHOMCL45	AF024	Trypsin-like serine protease	0
Ld_07966	ORTHOMCL85	AF037	Alpha/beta hydrolase and Pancreatic lipase family	0
Ld_05193	ORTHOMCL86	AF030	Papain-like cysteine protease	0
Ld_10503	ORTHOMCL86	AF030	Papain-like cysteine protease	0
Ld_05064	ORTHOMCL87	AF031	Enolase	0
Ld_10276	ORTHOMCL87	AF031	Enolase	3
Ld_01332	ORTHOMCL5	AF049	ATP:guanido phosphotransferase family	0
Ld_04787	ORTHOMCL88	AF024	Trypsin-like serine protease	0
Ld_11513	ORTHOMCL88	AF024	Trypsin-like serine protease	0
Ld_04574	ORTHOMCL89	AF024	Trypsin-like serine protease	0
Ld_05439	ORTHOMCL89	AF024	Trypsin-like serine protease	0
Ld_04061	ORTHOMCL90	AF024	Trypsin-like serine protease	0
Ld_07788	ORTHOMCL90	AF024	Trypsin-like serine protease	0
Ld_00721	ORTHOMCL91	AF038	Cyclophilin	0
Ld_06546	ORTHOMCL91	AF038	Cyclophilin	0
Ld_06076	ORTHOMCL96	AF024	Trypsin-like serine protease	0
Ld_09793	ORTHOMCL97	AF024	Trypsin-like serine protease	0
Ld_06725	ORTHOMCL98	AF030	Papain-like cysteine protease	0
Ld_07227	ORTHOMCL7	AF024	Trypsin-like serine protease	0
Ld_09013	ORTHOMCL7	AF024	Trypsin-like serine protease	0
Ld_11006	ORTHOMCL7	AF024	Trypsin-like serine protease	0
Ld_11041	ORTHOMCL7	AF024	Trypsin-like serine protease	0
Ld_12446	ORTHOMCL7	AF024	Trypsin-like serine protease	0
Ld_12530	ORTHOMCL7	AF024	Trypsin-like serine protease	0
Ld_01565	ORTHOMCL60	AF167/AF023	AhpC/TSA family and Thioredoxin	1
Ld_10100	ORTHOMCL100	AF024	Trypsin-like serine protease	0
Ld_03528	ORTHOMCL8	AF010	Glutathione S-transferase	1
Ld_11419	ORTHOMCL8	AF010	Glutathione S-transferase	1

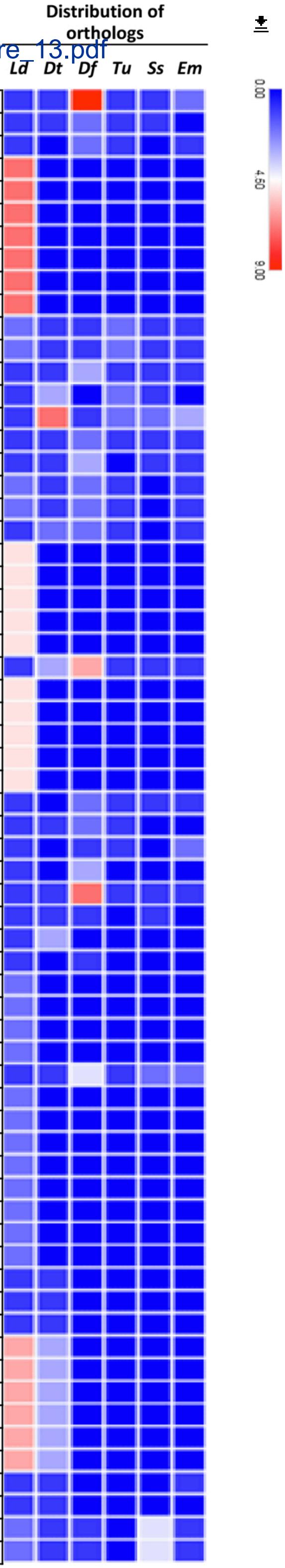

Figure 14

Click here to access/download;Figu

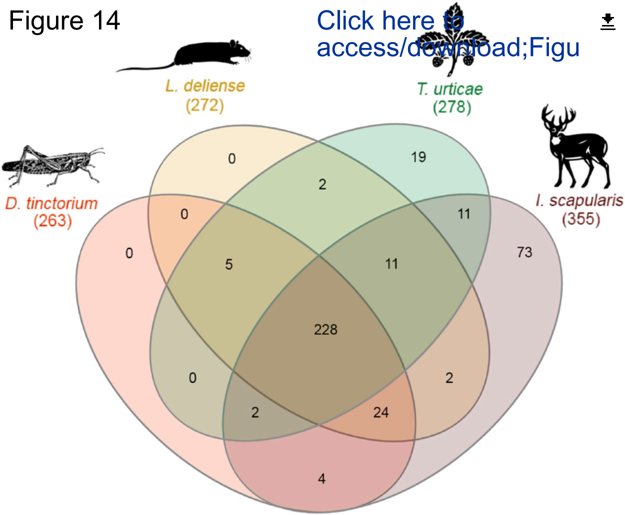

Click here to access/download
Supplementary Material
Additional file 1.pdf

Click here to access/download
Supplementary Material
Additional file 2.xlsx
