# Supplementary material for: Genomes of trombidid mites reveal novel predicted allergens and laterally transferred genes associated with secondary metabolism
Source: Gigascience. 2018 Nov 15;7(12):giy127. doi: 10.1093/gigascience/giy127 (PMC6275457; doi:10.1093/gigascience/giy127)
Supplement: Supplemental Files [file giy127_supplemental_files.zip › Additional file 1.pdf]

A

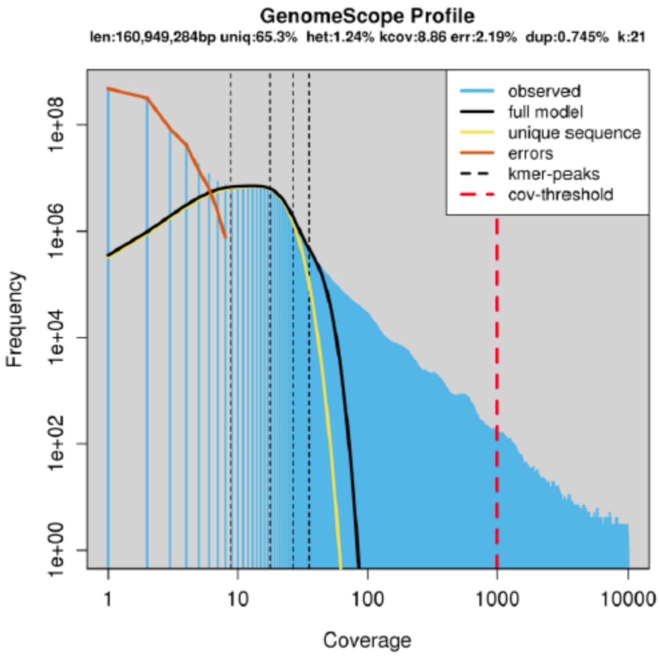

B

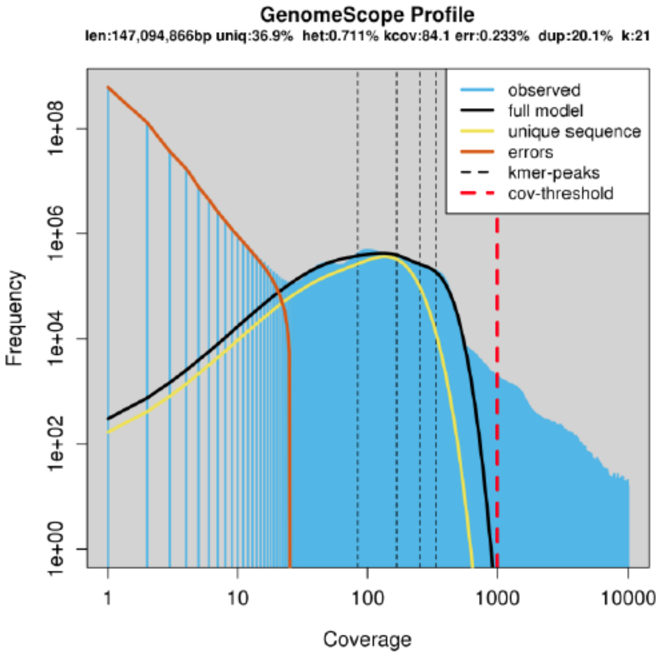

Supplemental figure S1: *K*-mer distributions for *Leptotrombidium deliense* (*A*) and *Dinothrombium tinctorium* (*B*) plotted by GenomeScope.

Supplemental table S2: Identification of repetitive sequences in the *Dinothrombium tinctorium* and *Leptotrombidium deliense* assemblies compared with other acariform mites.

Repeat Type	*D. tinctorium*		*L. deliense*		*T. urticae*		*S. scabiei*	
	Length (bp)	% of genome	Length (bp)	% of genome	Length (bp)	% of genome	Length (bp)	% of genome
**SINEs:**	208,192	0.11	250,318	0.22	10,240	0.01	17,800	0.03
**ALUs**	0	0	117,513	0.1	0	0	0	0
**MIRs**	365	0	52,546	0.04	1,728	0	335	0
**LINEs:**	1,008,042	0.56	710,254	0.61	96,225	0.1	33,262	0.06
**LINE1**	1,565	0	382,470	0.33	2,784	0	11,005	0.02
**LINE2**	41,841	0.02	44,477	0.04	9,002	0.01	1,050	0
**L3/CR1**	82,461	0.05	110,392	0.1	18,527	0.02	2,260	0
**LTR elements:**	1,458,802	0.81	729,753	0.62	903,530	0.99	82,362	0.14
**ERVL**	70	0	47,531	0.04	205	0	1,104	0
**ERVL-MaLRs**	106	0	85,526	0.07	0	0	931	0
**ERV_classI**	144,113	0.08	45,552	0.04	408	0	38,792	0.07
**ERV_classII**	219	0	32,043	0.03	221	0	144	0
**DNA elements:**	3,807,377	2.11	1,148,765	0.98	589,972	0.65	231,195	0.41
**hAT-Charlie**	1,258	0	39,098	0.03	673	0	1,397	0
**TcMar-Tigger**	2,055	0	24,417	0.02	2,852	0	80	0
**Unclassified:**	32,486,993	18.01	17,403,455	14.83	7,140,708	7.86	392,415	0.7
**Small RNA:**	127,843	0.07	43,167	0.04	331,069	0.37	18,152	0.03
**Satellites:**	0	0	823	0	0	0	0	0
**Simple repeats:**	1,887,603	1.04	958,576	0.81	1,881,927	2.07	3,930,487	6.98
**Low complexity:**	798,433	0.44	230,362	0.2	491,918	0.54	574,609	1.02
**Total:**	42,057,338	23.3	22,457,038	19.15	11,481,989	12.62	5,337,380	9.46

Supplemental table S3: Impact of Redundans analysis on genome statistics for *Dinothrombium tinctorium.*

Statistic		Original assembly	Redundans refined scaffolds
Assembled genome size		180 Mb	114 Mb
Scaffolds number		22,761	12,493
Gene number		19,258	12,322
BUSCO analysis (%)	Complete	73	73
	Duplicated	34	12
	Fragmented	8.6	8.6
	Missing	17	18

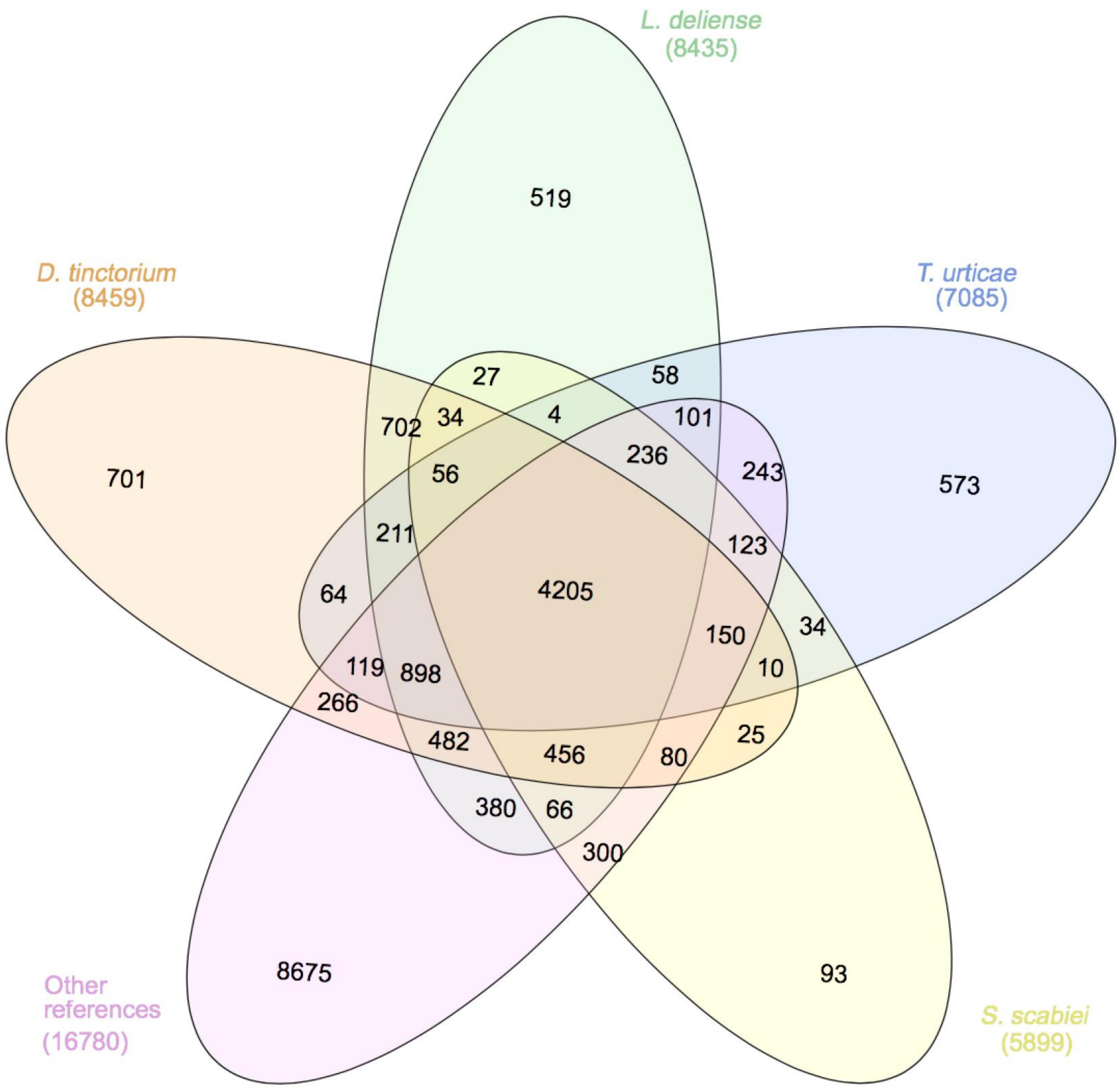

Supplemental figure S2: The number of gene families shared among acariform mites (*Dinothrombium tinctorium, Leptotrombidium deliense, Tetranychus urticae* and *Sarcoptes scabiei*); alongside other references including *Drosophila melanogaster, Apis mellifera, Tropilaelaps mercedesae, Metaseiulus occidentalis, Ixodes scapularis, Stegodyphus mimosarum* and *Caenorhabditis elegans* by the OrthoMCL classification algorithm.

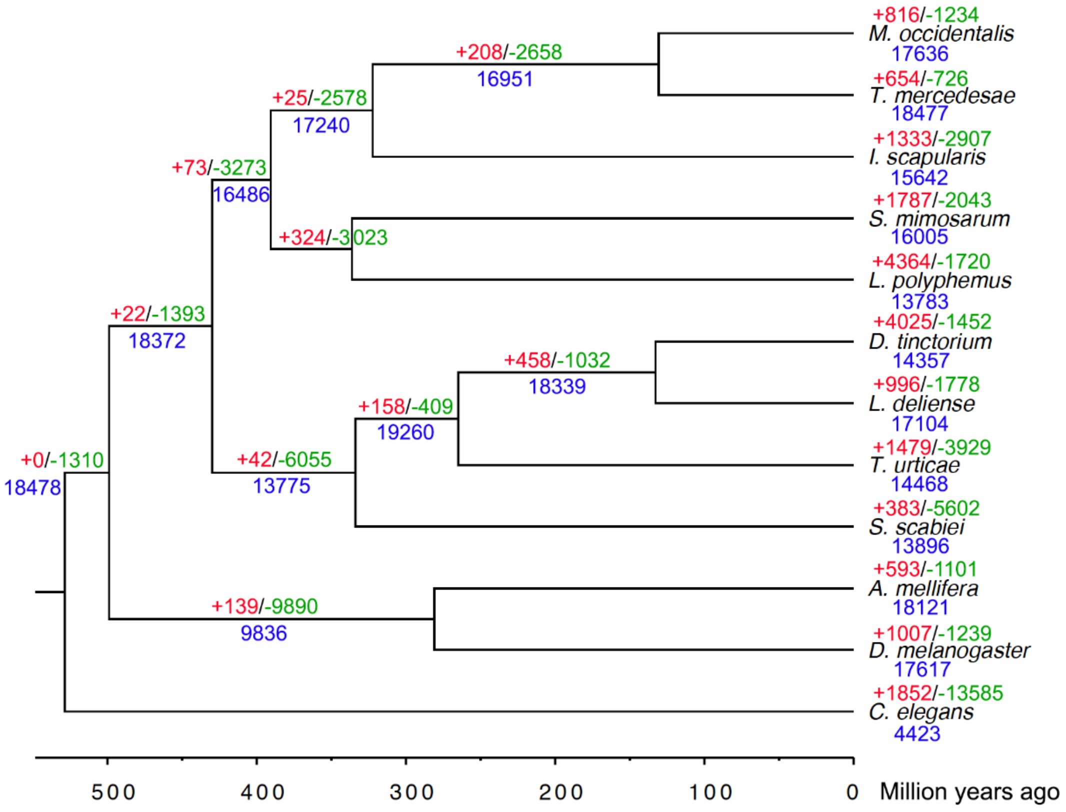

Supplemental figure S3: Gene family contraction and expansion in 12 species of Ecdysozoa. The numbers of expanded, contacted, and stable gene families in each species and node are indicated in red, green, and blue type, respectively.

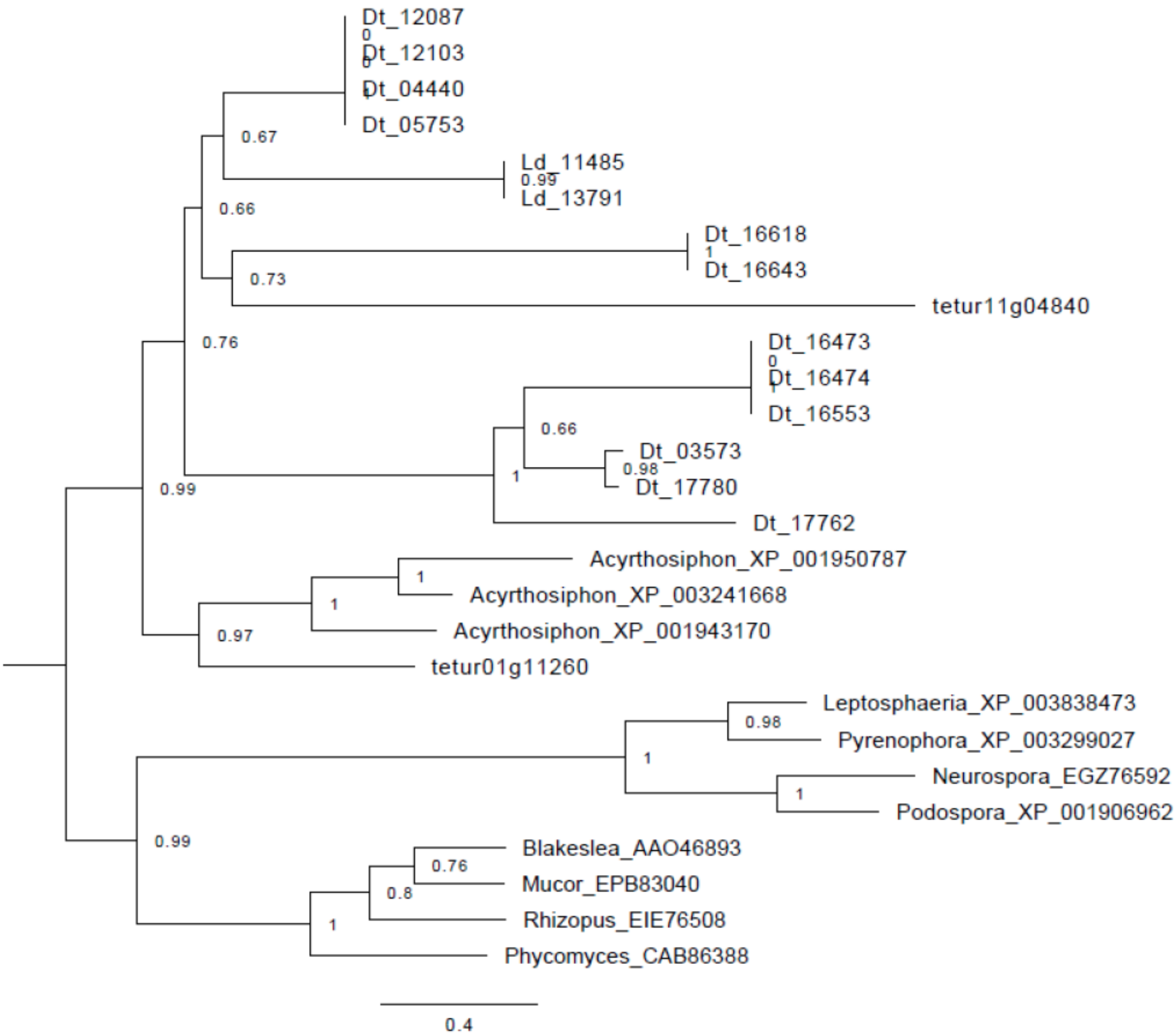

Supplemental figure S4: Phylogeny of carotenoid synthases-cyclases from trombidid mites, spider mites, aphids and fungi. The tree was constructed using a maximum-likelihood method. Prefixes: Dt = *Dinothrombium tinctorium*; Ld = *Leptotrombidium deliense*; tetur = *Tetranychus urticae*. *Acyrthosiphon* sequences derive from the pea aphid, *Acyrthosiphon pisum*. *Leptosphaeria*, *Pyrenophora*, *Neurospora*, and *Podospora* are ascomycete fungi. *Blakeslea*, *Mucor*, *Rhizopus* and *Phycomyces* are zygomycete fungi.

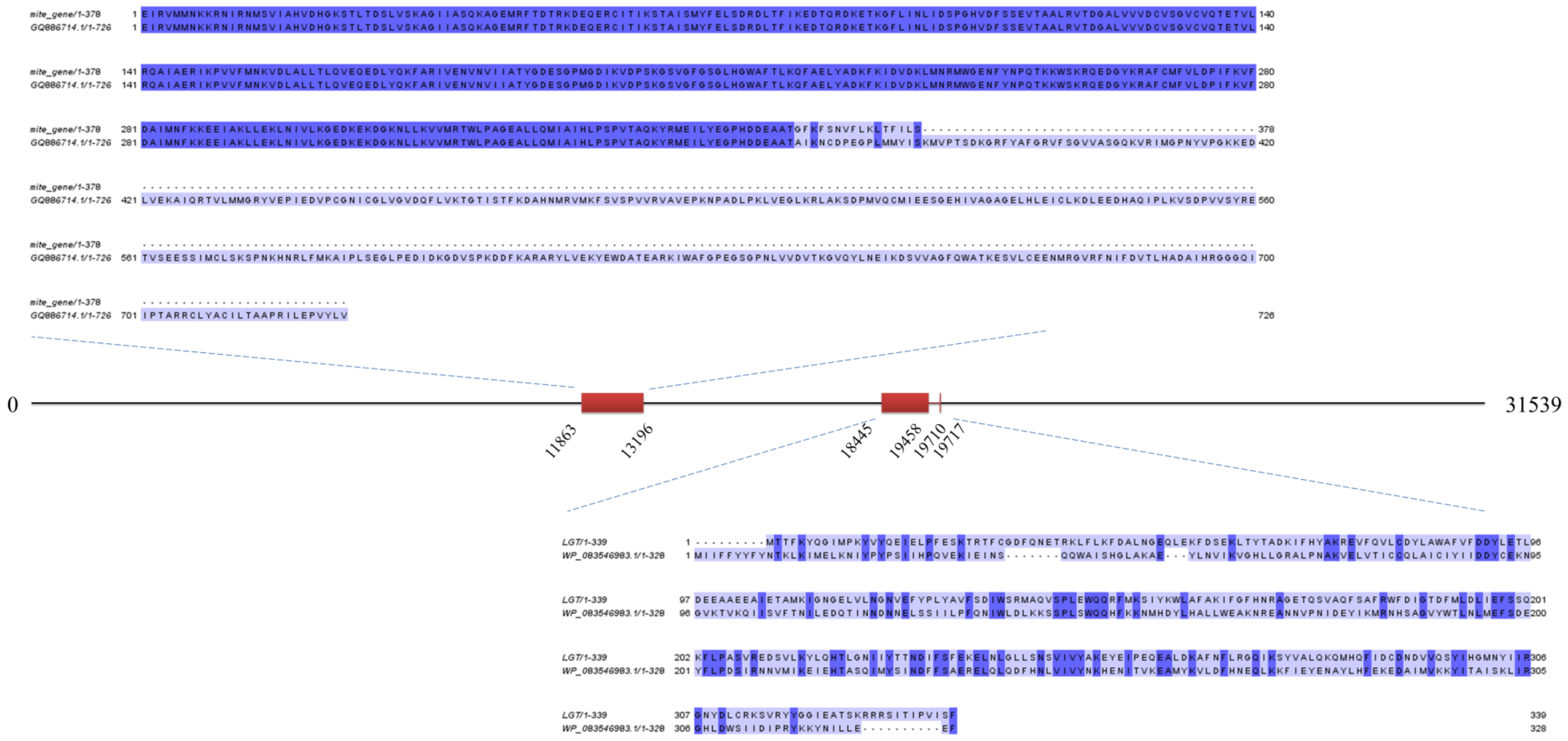

Supplemental figure S5: Genomic scaffold of *Dinothrombium tinctorium* containing a putative lateral gene transfer adjacent to an incontrovertible mite gene. <u>Top</u>: Alignment of a *D. tinctorium* translated ORF with a translated mRNA transcript (translational elongation factor-2, GenBank accession GQ886714.1) from *Dinothrombium pandorae*. <u>Bottom</u>: Alignment of a translated, putatively laterally-transferred *D. tinctorium* coding sequence with a hypothetical protein from *Chryseobacterium polytrichastri* (NCBI Reference Sequence WP_083546983.1), representing the top BLAST hit. These genes both contain a class I terpene cyclase conserved domain. Identical residues are highlighted in violet shading. <u>Center</u>: Schematic representation of the genomic scaffold with exons shown as red boxes. Nucleotide positions are numbered.

Supplemental table S9: Microbial reads identified in the trombidid genomic data by the Kraken taxonomic sequence classification system.

			Percent								
Species	Library	Number of raw reads	Classified reads	Chordate reads	Artificial reads	Unclassified reads	Microbial reads	Bacterial reads	Viral reads	Fungal reads	Protozoan reads
L. deliense	Next Ultra 550 bp	19,135,768	0.640	0	0	99.4	0.640	0.115	0.198	0	0
D. tinctorium	TruSeq 350 bp	100,545,764	0.165	0	0	99.8	0.165	0.124	0.015	0	0
	TruSeq 550 bp	65,441,221	0.0605	0	0	99.9	0.0605	0.0208	0.0144	0	0
	Nextera 3 kb	4,223,074	0.415	0	0	99.6	0.415	0.0436	0.294	0	0

```
Dt_16422     1   MTKIVYQELDIPVESKISSFAEEIQENCELLFYKYNIFDETKKSEFHEQKITTFPTRVYN    60
Dt_16258     1   MAKIIYQELDIPIESKLNSFADEIQENCELLFDKYNIFDEAKKNDFHEQKLTFYASRGFH    60
                 *:**:*******:***:.***:********** *******:**.:*****:* : :* ::

Dt_16422    61   YASKEILQIVSNFLAWTFVFDDYIETHHYDRDETCERVLQIGYGNLSLKEGEALRSLEAL   120
Dt_16258    61   YASKEVLQILANYFTWGFIFDDYMETHHNEREETCQRVLQIGNGNLCLKEGELLRPLEEL   120
                 *****:***::*:::* *:****:**** :*:***:****** ***.***** ** ** *

Dt_16422   121   FSEIWNSIKKQSPNEWQKRFIDSIRNWFVFTNILMKHKMNGTIPTMAEFISYRWFDAASD   180
Dt_16258   121   FSEIWDSIKQRSPNEWQKRFIDALRNWFVFVHILMNHKVNDTNPTLAEYISYRWFDFGSD   180
                 *****:***::***********::******.:***:**:*.* **:**:******* .**

Dt_16422   181   MAINLIEFAMEKFLPQSFHNEMVIQHLVHANGNVIGAINDIFSYEKDIRADNNLNFVEVM   240
Dt_16258   181   MIILMIEFAMQKFLPQSYHKEMVMQHIVHALGNVIASINDIFSYEKETRAGNHLNLVAVI   240
                 * * :*****:******:*:***:**:*** ****.:*********: **.*:**:* *:

Dt_16422   241   KNEMKIEVQEAIDRRYDFTRAQIQSYFLLRKQLNRIFDPNDEVIKKYVDGMDSLMRGHYD   300
Dt_16258   241   KNEMKIEVQEAMDRAYDFNRVQIQSYFSLRKQLNRIFDPNDEVIKKYVNGMDTLMRGNYD   300
                 ***********:** ***.*.****** *******************:***:****:**

Dt_16422   301   FYFDGNRYFEICPFKSDSRMLTNSNDFEF                                 329
Dt_16258   301   FYFDGNRYFENCPFKSDSTNADK---FE-                                 325
                 ********** *******    :   **
```

Supplemental figure S6: Peptides (pink shading) detected by mass spectrometry from two terpene synthases in an adult specimen of *Dinothrombium tinctorium*.

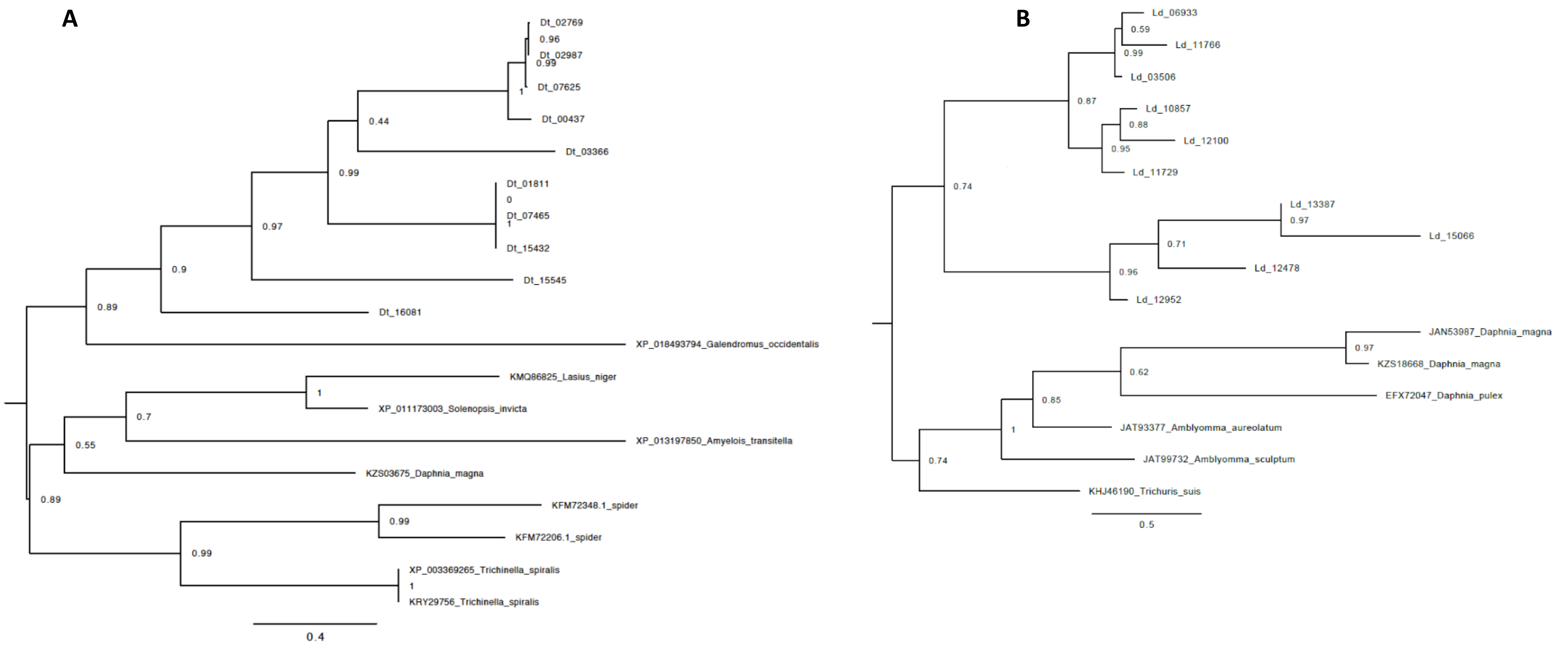

Supplemental figure S7: Phylogeny of reverse ribonuclease integrases in trombidid mites and their closest homologues in other taxa. A, ORTHOMCL3978; B, ORTHOMCL6070. The tree was constructed using a maximum-likelihood method. Prefixes: Dt = *Dinothrombium tinctorium*; Ld = *Leptotrombidium deliense*. Other integrase sequences are derived from ants (*L. niger*, *S. invicta*), lepidopterans (*A. transitella*), nematodes (*T. spiralis, T. suis*), cladocerans (*D. magna, D. pulex*) and parasitiform mites or ticks (*G. occidentalis*, *T. mercedesae, A. aureolatum, A. sculptum*).

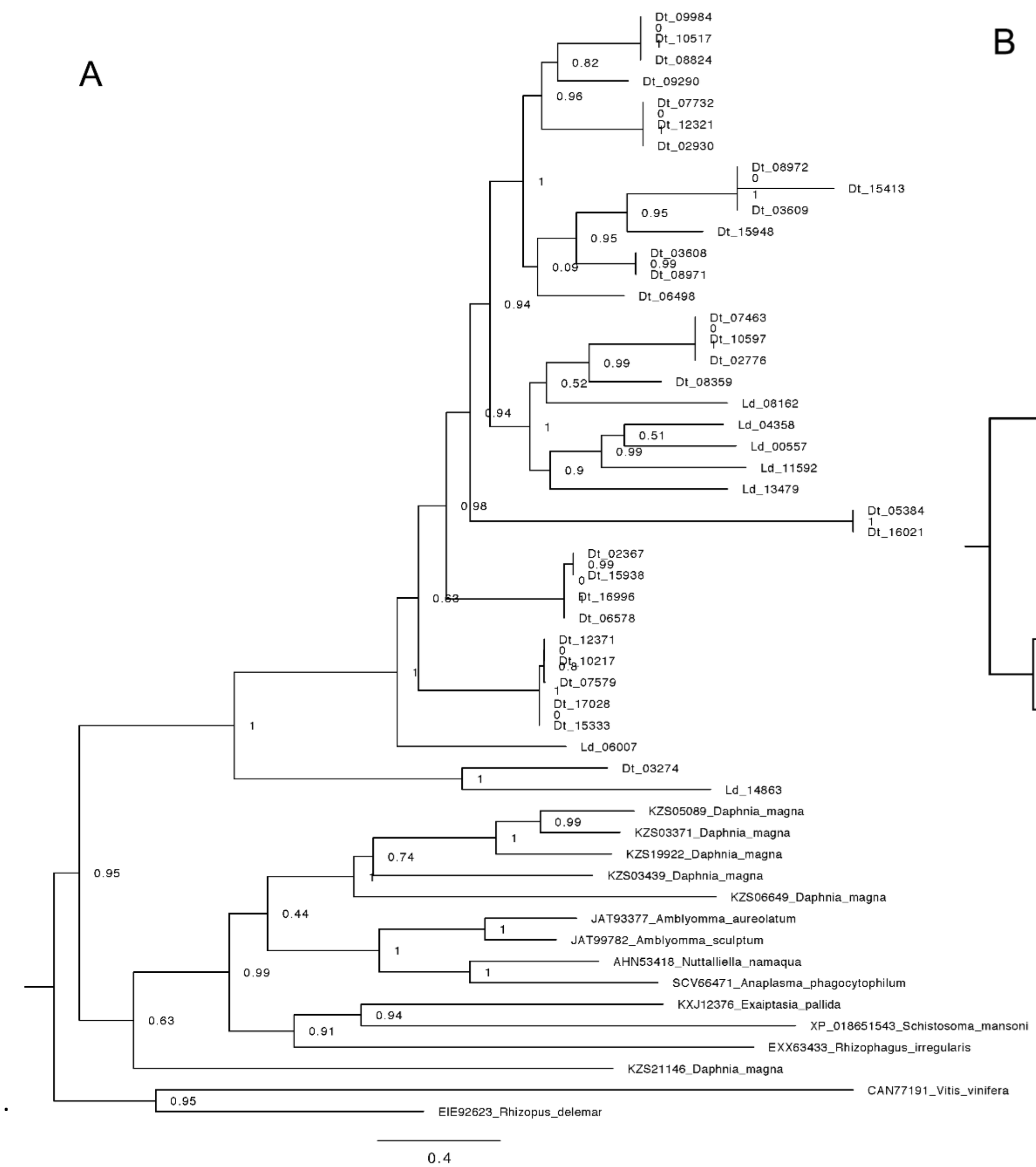

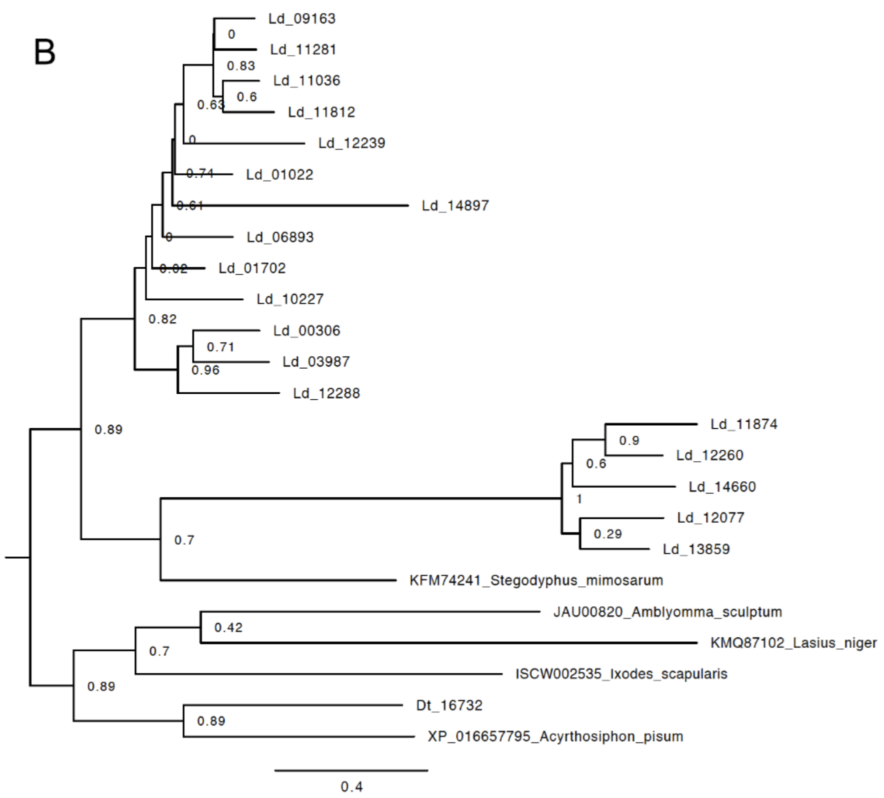

Supplemental figure S8: Phylogeny of Pol-like polyproteins in trombidid mites and their closest homologues in other taxa. A, ORTHOMCL161; B, ORTHOMCL682. Trees were constructed using a maximum-likelihood method. Prefixes: Dt = *Dinothrombium tinctorium*; Ld = *Leptotrombidium deliense*. Other Pol-like polyprotein sequences are derived from aphids (*A. pisum*), ants (*L. niger*), cladocerans (*D. magna*), ticks (*I. scapularis, A. aureolatum*, *A. sculptum*, *N. namaqua*), spiders (*S. mimosarum*), α-proteobacteria (*A. phagocytophilum*), actiniarians (*E. pallida*), trematodes (*S. mansoni*), rosid angiosperms (*V. vinifera*), arbuscular mycorrhizae (*R. irregularis*) and zygomycete fungi (*R. delemar*).

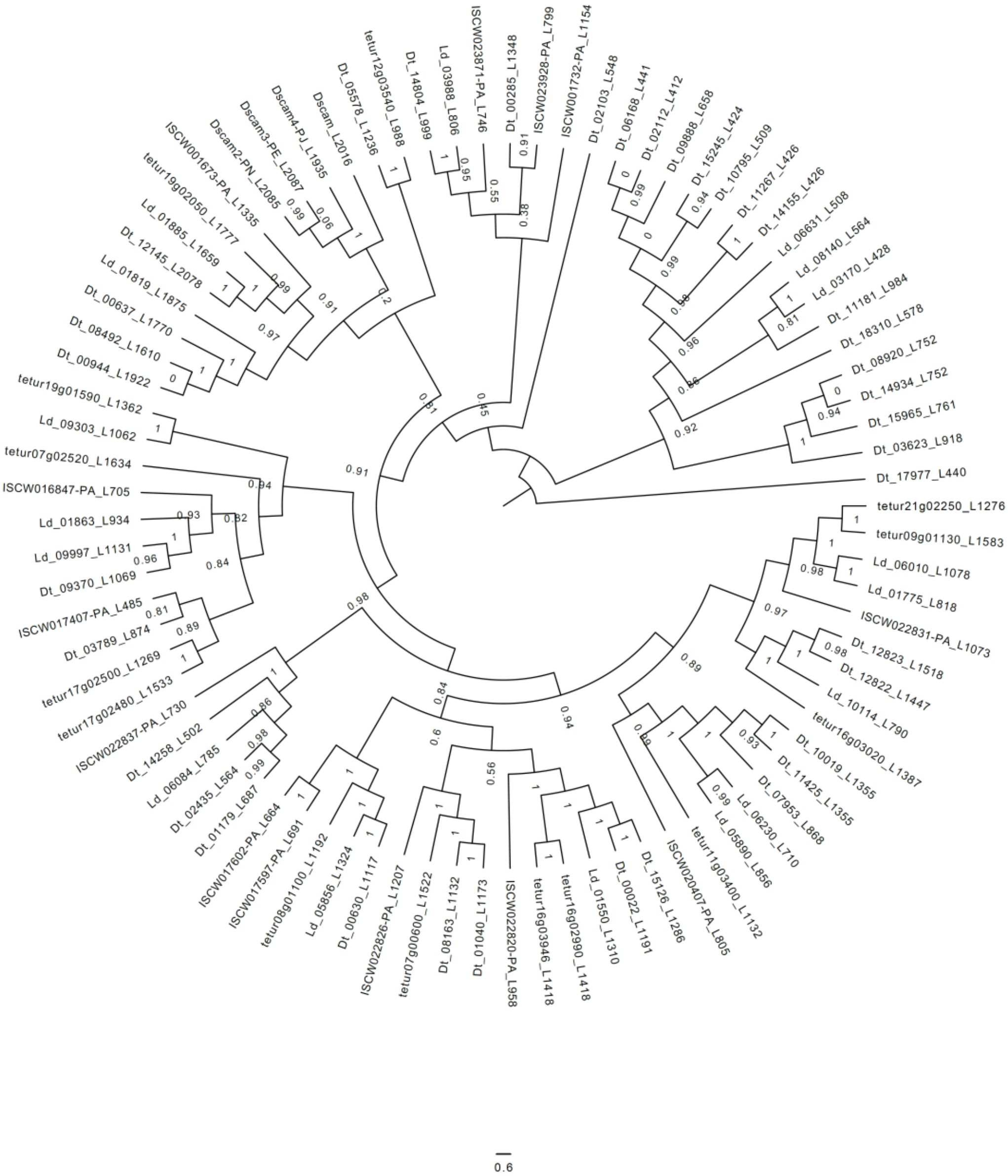

Supplemental figure S9: Phylogeny of Dscam protein-coding sequences in *Dinothrombium tinctorium*, *Leptotrombidium deliense*, *Tetranychus urticae* and *Ixodes scapularis*. The tree was constructed using a maximum-likelihood method. Prefixes: Dt = *D. tinctorium*; Ld = *L. deliense*; tetur = *T. urticae*; ISCW = *I. scapularis*. Dscam gene names without a prefix are from Drosophila melanogaster. Suffixes "LXXXX" refer to the number of amino-acid residues in the sequence. Only sequences with >400 residues were included in the tree to reduce the risk of including split gene models or pseudogenes.

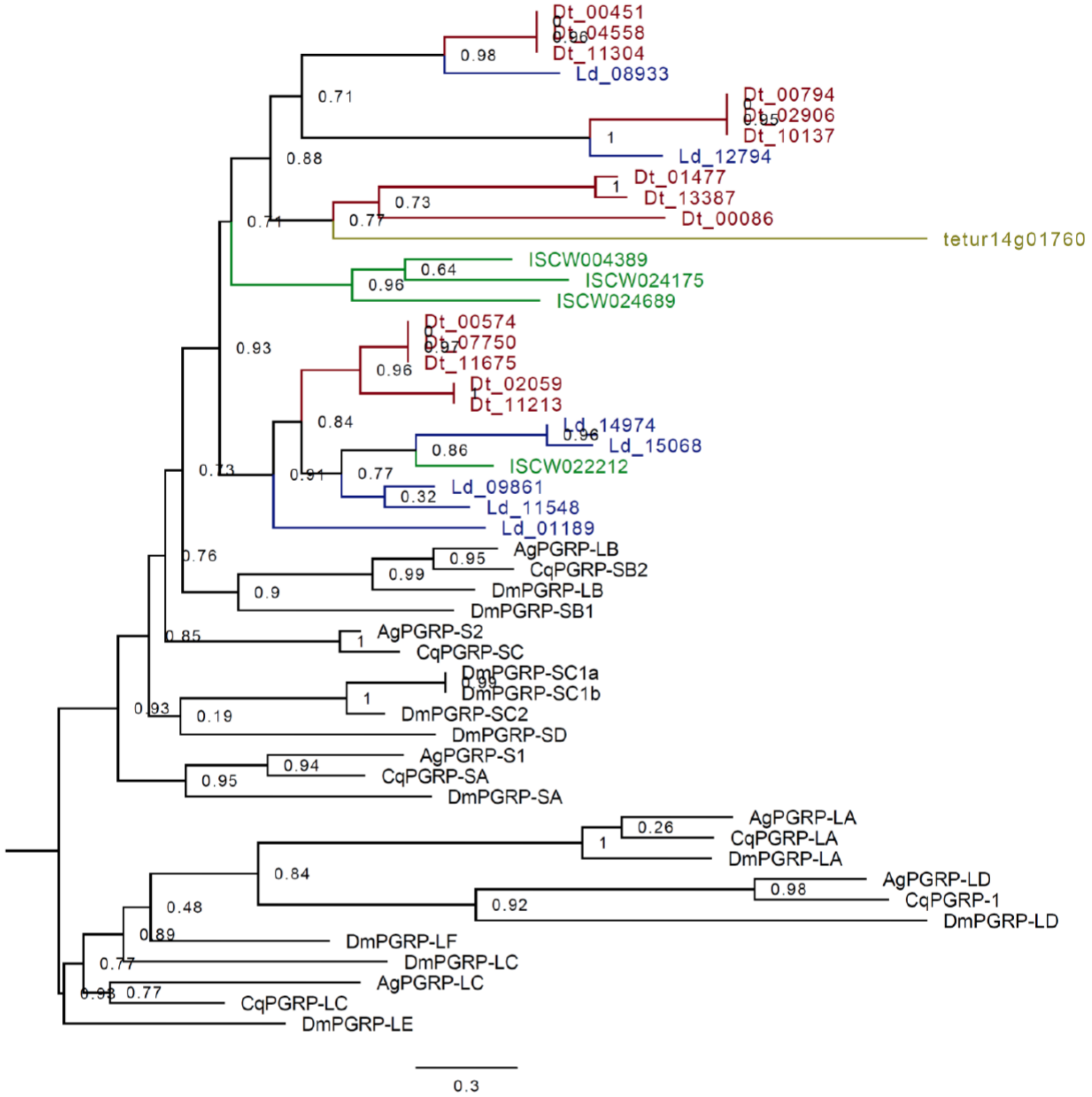

Supplemental figure S10: Phylogeny of peptidoglycan recognition protein sequences in *Dinothrombium tinctorium*, *Leptotrombidium deliense*, *Tetranychus urticae* and *Ixodes scapularis* alongside homologous sequences from insects. The tree was constructed using a maximum-likelihood method. Colour key: Red = *D. tinctorium*; blue = *L. deliense*; yellow = *T. urticae*; green = *I. scapularis*. Prefixes for insect genes: Dm = *Drosophila melanogaster*; Ag = *Anopheles gambiae*; Cq = *Culex quinquefasciatus*.

Supplemental table S12: Orthologous clusters of tick cement proteins in the genomes of *Dinothrombium tinctorium* and *Leptotrombidium deliense*.

**OrthoMCL group**	***D. tinctorium* gene ID**	***L. deliense* gene ID**	**Tick gene ID[a]**
ORTHOMCL443	Dt_11640; Dt_14591; Dt_14932; Dt_15944	Ld_02702	Aa_AIR95099.1; Aa_AIR95100.1
ORTHOMCL748	Dt_05514; Dt_09034; Dt_12049	Ld_11492	Is_EEC15669.1
ORTHOMCL4226	Dt_02264	Ld_00535	At_JAC30256.1
ORTHOMCL6307	Dt_10354	-	Is_EEC02709.1

[a]Aa: (*Amblyomma americanum*); Is: (*Ixodes scapularis*); At: (*Amblyomma triste*).

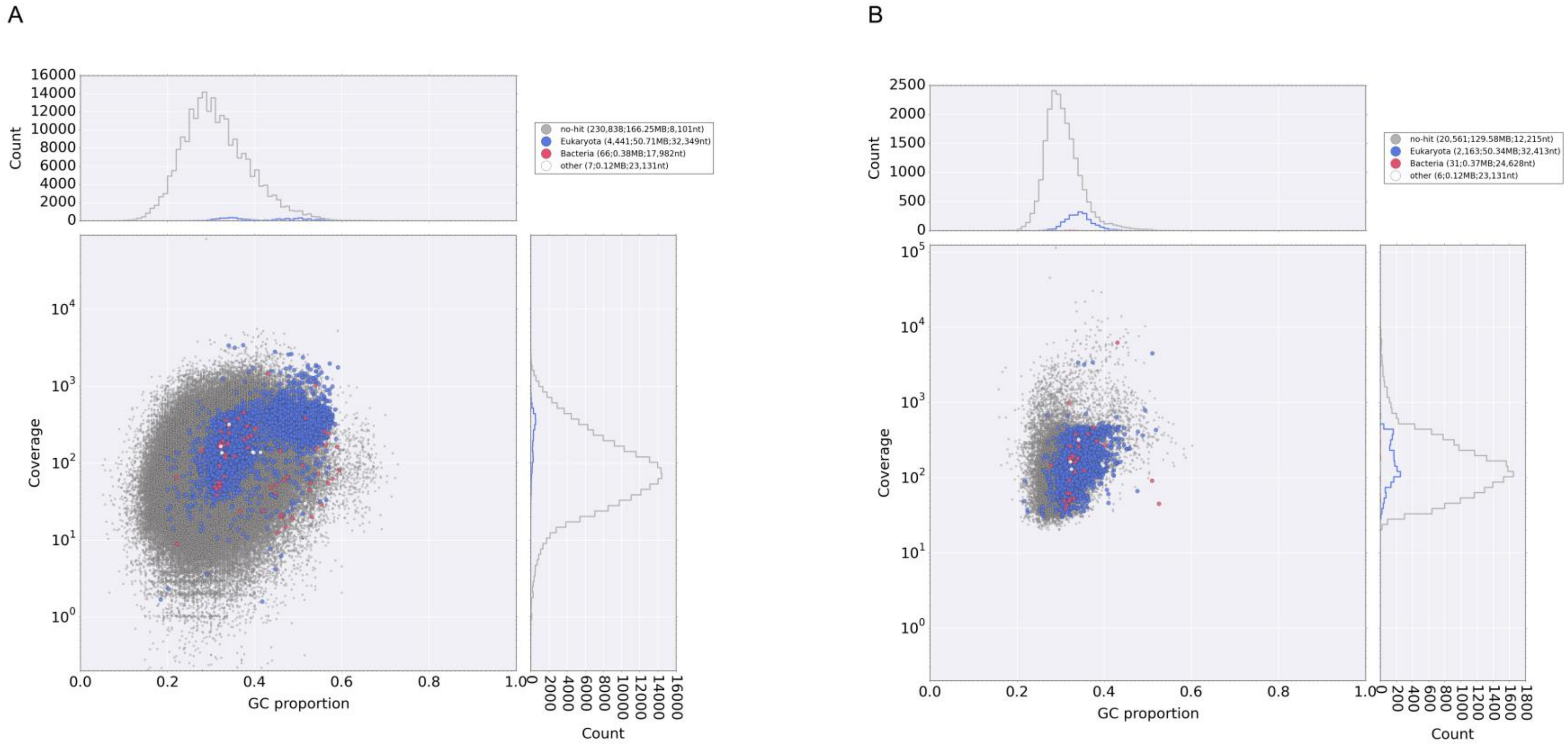

Supplemental figure S11: Blob-plot of *D. tinctorium* genomic scaffolds before (A) and after (B) removal of small scaffolds (<1,000 bp).
