# Supplementary material for: Genomes of trombidid mites reveal novel predicted allergens and laterally transferred genes associated with secondary metabolism
Source: Gigascience. 2018 Nov 15;7(12):giy127. doi: 10.1093/gigascience/giy127 (PMC6275457; doi:10.1093/gigascience/giy127)
Supplement: Response_to_Reviewer_Comments_Original_Submission.pdf [file giy127_response_to_reviewer_comments_original_submission.pdf]

Author's Response To Reviewer Comments

Close

Dear Dr Zauner,

Thank you for communicating the referee reports for our manuscript and your own comments.

Please see our detailed point-by-point rebuttal below. Page and line numbers refer to the "Track Changes" version.

Editor's comments:

When discussing your submission internally with the GigaScience team, we also wondered whether the proteomics part may be a bit too preliminary, given that only a limited selection of developmental stages and in one case only a single individual was analysed, if we understand correctly. Actually, I intended to ask "reviewer 3" specifically about technical aspects of the proteomics part, but this reviewer unfortunately had to withdraw from the review process, as mentioned above. I don't want to delay our initial decision any further at this point, but I hope you can explain limitations of the preliminary proteomics part in more detail in the manuscript. We may need to briefly discuss this specific aspect with an external proteomics expert on re-review.

>We agree that the proteomics analysis could be considered preliminary, and we describe it as such in the abstract. However, we have now also emphasised this is the Results text too (p. 12, line 236) and in our responses to Referee 1's critique of the proteomics (leading to a new concluding sentence for the whole manuscript). We would maintain that preliminary proteomic data are better than no expression data for these elusive species, and that these data complement the genomes. For instance, the chigger proteomic data provide important insights into the expression of potentially allergenic proteins in a pooled larval sample. While the D. tinctorium proteomic analysis is based on a single individual, its large size allowed fractionation and the robust identification of >1,600 proteins, which is quite respectable for a single sample. Importantly, these high-confidence identifications included one of the laterally-transferred terpene synthases (supplemental fig. S5).

It is important to emphasise the extreme difficulty of obtaining trombidid mite material, especially from more than one lifecycle stage. Engorged larval chiggers are relatively straightforward to obtain, but are 0.25 mm long and contaminated with host material. Questing chiggers in the environment are very difficult to collect in any numbers, and are even smaller; while the nymphs and adults live mainly underground and are virtually impossible to locate in their natural environment. We only know of one lab colony of L. deliense maintained worldwide, located in the Armed Forces Research Institute of Medical Sciences in Bangkok. We are actively seeking material from additional stages from this colony, but do expect to be able to obtain additional data in the short term due to import/export requirements. For D. tinctorium, no lab colonies exist and obtaining material is dependent on mass emergence of adults after rains, which tend to occur once annually. The parasitic larvae are dispersed at low rates of infection, mainly on orthopteran hosts.

Reviewer reports:

Reviewer #1: The authors report the genomes of two important trombidid mites, namely the giant red velvet mite, Dinothrombidium tinctorium, the world's largest acarid species and the chigger, Leptotrombidium delicense, the primary scrub typhus vector in South-East Asia. This brings the number of Acariform genomes to four and allows important insights into the biology and evolution of the Acari. Specific horizontal gene transfer of terpene and carotenoid synthesis genes from soil-derived bacteria and fungi are illuminated as unique features of trombidid mites. A variety of protein families involved in sensing were analyzed including gustatory, olfactory and ioinotropic receptors, odorant binding and chemosensory proteins and opsins. The results support earlier observations that olfactory receptors and odorant binding proteins are absent in the Arachnida. Potential new allergens were predicted and salivary orthologs shared among Acari identified. Proteomic analysis of whole body extracts from adult red velvet mites and larval chiggers also identified an extensive number of proteins encoded in the genome.
The sequencing and genome assembly follows well validated methods and BUSCO analysis indicate that completeness of the genomes are similar to other published acarid genomes to date. Similarly, the proteomics methods may be considered part of standard workflows and the results obtained should be seen in the light of the complexity of whole organism extracts. The statistics reported and employed are appropriate in all analyses. Overall, the study is comparable to others of its kind. The study adds an extensive amount of new data that makes this an important contribution within its field. It is well written, the methodology followed clear and understandable. Most of the conclusions are warranted and the manuscript was instructive and enjoyable to read. I have no doubt that this study is worthy of publication. Some issues follow below that should be considered.

>We thank the referee for their very positive view of our manuscript.

Page 9, lines 162-169: The author's presents an interesting divergence date analysis (Fig. 4). The exclusion of the horse-shoe crab from the analysis, for which genomes have been sequenced is noteworthy and could affect some of the estimated divergence dates. Other nodes that may need to be considered is that of the crown Ecdysozoa (Nematode-Arthopod split) that was recently calibrated at 528.8-636 MYA (Benton et al. 2015). The split between Chelicerata and Mandibulata should also be considered to be constrained at 514-636 MYA (Benton et al. 2015). This would make all molecular clock estimates slightly older than in the current analysis. It would also affect some of the conclusions made in the discussion (Page 26, line 588).

>Following the referee's suggestion, we included the horseshoe crab data in the phylogenomic analysis and have changed Figures 1 and 4 accordingly. As the referee noted, this addition does change our divergence times slightly (for instance, the split between our newly sequenced mite species is now ~10 million years earlier than previously estimated). However, this difference is too small to affect our conclusions. The modified molecular clock estimates also affect the CAFE analysis slightly, necessitating small changes to Figure 9 and supplemental fig. S2, supplemental table S5, and supplemental figs. S7 and S8. We have also included some new text (p. 29, lines 662-667) in which we highlight the problems with resolving the position of the major divisions (classes and orders) within the Chelicerata caused by long-branch attraction, which has been considered in depth by other authors (see doi:10.1093/molbev/msu235).

Page 9, line 158; Page 37, line 867: The impressive CEGMA score may be amended to also include the BUSCO scores that is not so impressive and does indicate that the genomes are not as complete as suggested. While the completeness of the genomes compare well with other genomes sequenced to date, it may be good to indicate that for the majority of acarid genomes we still miss ~15-20% of predicted gene data. It should also be underscored that CEGMA/BUSCO gives an indication of housekeeping genes and that numbers for lineage specific expanded genes (gene duplicates) may be under represented.

>We agree that there appear to be significant issues with genome completeness across the Chelicerata. Challenging genomic features like repeat content and gene duplication (or even whole genome duplication; see DOI 10.1186/s12915-017-0399-x) may account for a large proportion of the missing data in short-read assemblies. While our manuscript has been in revision, two acarine long-read assemblies have been published with BUSCO scores of >90%, one from a tick cell line (10.12688/f1000research.13635.1) and another from a very large pool of sheep scab mites (doi.org/10.1128/genomeA.00265-18). Unfortunately, neither approach for obtaining large quantities of high molecular weight DNA (cell line development or pooling of thousands of individuals) is currently practicable for trombidid mites. Due to the very large size of D. tinctorium, a long-read assembly from a single individual may be possible in the future if additional fresh material can be located (see discussion under "Editor's comments" above). These issues are discussed on p. 27 – 28, lines 620 – 646.

Page 11, lines 201-231: It may be indicated that organ specific proteins (salivary proteins) may be underrepresented in the whole body proteomes, even if they might be highly abundant and present at concentrations that would make them important allergens or host modulatory molecules.

>This is a very important point, so now we actually emphasise it in the Conclusions (p. 37, lines 872 – 875).

Page 14, lines 279-282: Could the authors also state whether the average coverage for the terpene synthase was the same as the contig it was found in and whether this hold for the other terpene synthases as well. This would also support it as a bona fide LGT. On this, the authors do not explicitly mention this, but the phylogenetic trees suggest lineage specific expansions (gene duplication) of this family in these mites. Does this mean that the genes are scattered through the genome or are they found on limited scaffolds/chromosomes?

>In almost all cases, the coverage for the terpene synthase gene and its contig were very similar (please see new supplemental tables S8). Although we cannot comment on the structural organisation of the LGT genes due to lack of contiguity, the presence of some identical terpene synthase gene copies suggests tandem duplication.

Page 16, line 333-346: A third explanation for absence of the IMD pathway in acariform mites is that the Acari are paraphyletic and that immune deficiency pathways evolved in independent lineages after divergence of the chelicerata.

>We agree and have now included this possibility in the relevant text (p. 17, lines 366 – 367).

Page 22-25, line 487-564: The study by Kim et al. (2016) described proteins found in tick

salivary secretions. Of these, ~50% could be considered house-keeping with the caveat that ticks may secrete non-secretory proteins by apocrine secretion. Their functionality in feeding still needs to be demonstrated. The majority of orthologs found in Table 3 all seem to resemble house-keeping proteins and singletons. Given the absence of secretory protein families, this approach seems tenuous to identify salivary proteins from chiggers. What these results seem to suggest is that chiggers have a different salivary repertoire compared to ticks. It may be fruitful to look at highly expanded secretory families such as those reported in additional file 6.

>We agree that direct analysis of trombidid mite saliva would be greatly superior to in silico inferences, but is likely to remain beyond what is technically possible for some time due to the minute size of the larvae and the lack of an artificial feeding substrate. We believe that the number of orthologous clusters identified (as shown in Table 3) is not low when one considers that these are only the clusters shared uniquely between trombidid mites and the tick. Figure 14 shows that there are an additional >200 clusters shared between all four acarine species included in the analysis. The absence of certain tick gene homologues in the trombidid mite genomes, such as those involved in haem detoxification, makes good sense, as these mites are not blood feeders.

Regarding the number of putative salivary proteins that show features suggesting secretion, approximately 1/3 of the proteins in Table 3 contain classical or non-classical (internal) secretion signatures as predicted by SecretomeP. This information has been added to Table 3.

We agree that our analysis can only highlight conserved proteins that have a strong likelihood of being present in mite saliva, in contrast to secreted protein families in trombidid mites that are unique to this taxon. However, assuming that the expanded families of secreted proteins in supplemental table S5 are related to salivary function would be a big leap, as these proteins might be important in several other anatomical locations, such as the gut and haemolymph.

Page 47, line 1110: It was not clear that the genome assembly and related data has been submitted to the SRA database or Genbank public repositories, since no accession numbers will be associated with these sequences, nor will future BLAST analysis against the public databases be possible.

>The NCBI BioProjects for Dt and Ld are PRJNA377800 and PRJNA377801, respectively. The SRA raw datasets are linked to these, but the annotated genome datasets are still under review by the NCBI team. All data will also be made available on the GigaScience website if the manuscript is accepted.

Minor

Page 34, lines 790-798: The date for the collection of the chiggers is indicated, but not for the red velvet mites.

>We have now provided the collection date of the velvet mites (June 2010).

Page 38: line 882: Three ab initio.

>Corrected thank you.

Page 39: line 920: 926 orthologous are indicated, while the phylogenetic tree (Figure 1) states 527. No indication in text as to what happened to the 399 orthologs not included in the tree.

>We apologise for this error. The number of 1:1 orthologues is now reduced to 360 due to the addition of the horseshoe crab data.

Page 42, line 981: …chemosensory proteins in…

>Corrected thank you.

Page 44, line 1042: …prepared by homogenisation…

>Corrected thank you.

Reviewer #2: The authors report two draft genomes (one with quite low coverage) of trombidid mites, and provide a comprehensive analysis of the gene families that they found in these two species. At a time of long range sequencing with nanopore/pacbio/dovetail/hi-C, some might argue that these aren't great assemblies. However, I firmly believe that even lower quality draft genomes should be made available and analyses published as long as the limitations of the resources / analyses are clearly spelled out.

My biggest concern is that although some very comprehensive gene-family analyses have been presented in this manuscript, the genomes are of such low qualities that some of the findings may be artefacts. It is possible that it is very hard to get more DNA material from these species and therefore this is the best genomic resource we are going to have for a while. However, if the species can be resequenced to higher depths (thus mitigating the poor kmer plots in Addnl file 3), then the manuscript would be much more valuable. Either way, I'd like to see the issue of a poor genome assembly addressed more explicitly in the text, and all analyses put in that context.

>We certainly do believe that these trombidid mites genomes are the “best genomic resource we are going to have for a while”. The chelicerates provide substantial challenges to generating highly contiguous assemblies due to quirks of their biology not seen in most insects. In essence, small species tend to have very small genomes (such as the spider mite with a 90 Mb genome) and large species such as ticks, spiders and scorpions have large (several Gb) highly repetitive genomes, sometimes exhibiting whole-genome duplications. The genome size estimates for the trombidid mites fall somewhere between these two extremes.

Chelicerate researchers are thus forced to pool substantial numbers of individuals to obtain sufficient DNA from the smaller species, while the larger species would cost tens of thousands of pounds to sequence at high coverage with long-read technologies. These considerations explain why only two tick genomes using PacBio have been published recently. One of these had the advantage of being derived from a continuous cell line and achieved a BUSCO score of 95% (10.12688/f1000research.13635.1; published March 2018). The other (for the cattle tick Rhipicephalus microplus; see doi.org/10.1016/j.ijpara.2017.03.007) used relatively little long-read data in a hybrid

assembly and attained a BUSCO gene score (including fragments) of only ~40%. In addition, a spider genome was published last year using Dovetail but did not report BUSCO statistics (doi 10.1186/s12915-017-0399-x). While our manuscript was in revision, a PacBio assembly for the tiny genome (63.2 Mb) of another acariform mite (Psoroptes ovis) was published with a BUSCO score of 91% (see doi.org/10.1128/genomeA.00265-18), but used a pool of >3,000 individuals to obtain sufficient DNA. This would never be a practicable option for chiggers. A discussion of these issues is now included on p. 27 – 28, lines 622 – 636.

The velvet mite (Dt) might make a good target for a long-read assembly due to its large size, but we only obtained the material used for the Illumina libraries due to a chance event (a collaborator was present during a mass mite emergence from underground during heavy rains; a once-per-year occurrence). As explained above in response to the editor's comments, chigger larvae are miniscule and contaminated with host material, while the free-living adults are only a few mm in size and are almost never encountered in the wild. While we are negotiating to obtain adult chiggers from AFRIMS in Bangkok, we estimate that a long-read assembly could take at least 18 months to sequence, analyse and submit for publication, as multiple extraction attempts will be required to obtain sufficient high molecular-weight DNA.

Manuscript presentation comments: This is more of a formatting request but one that will hopefully make life easier for the readers of the final paper: please consider consolidating all supplemental tables into one excel (or other spreadsheet software) file with separate tables; please put all supplemental figures into one pdf rather than a separate pdf for each figure. Some of the tables are also submitted as additional PDF files (eg Additional Files 3, 20) when they could clearly be an extra separate supplemental tables inside a single excel file.

>We have now combined the Excel tables and document files into two consolidated supplemental files as requested.

Several figures in the main manuscript could also be consolidated into single figures. For example, all the phylogenetic trees could be subparts of one figure, which would make it easier to compare the findings of different analyses.

>In order to retain legibility, we prefer to keep most of the trees as separate figures, although some of the supplemental trees have been combined (see supplemental figs. S7 and S8).

Please also consider sending the resubmission and future manuscripts with inline figures and tables (like a thesis). Modern production systems can easily deal with such manuscripts. It doubles the effort for both the writers and reviewers if there are separate documents for text, figures, tables, legends.

>We sympathise with the reviewer on this point, but are following current GigaScience manuscript preparation guidelines (which may change soon).

Background:
Excellently written - thank you. I learnt lots about why these species / clades are important. A general note: I freely admit I'm no systematist, and I don't know what the convention is, but I would have guessed taxon names should be considered as plural nouns unless prefixed with the rank. Eg, "Most Nematoda are ...", but "The phylum Nematoda is ..." sound correct

to me. Am happy to be told I'm wrong if that's not the convention, but this manuscript needs to be consistent. Some lines treat taxa as singular "The Acari is ...", "The Parasitiformes harbours ...", whereas others treat taxon names as plural "of the Trombidiformes in response to their ...".

>We have changed the text to be more consistent on this point, treating taxa as plural unless preceded by rank.

In the data description, I suggest using the scientific names or acronyms (Dt / Ld) rather than switching back and forth between Dt and velvet mite, and Ld and chigger.

>We have now used the species names rather than the colloquial names throughout, apart from the Introduction and Discussion, where the colloquial names are sometime useful to emphasize certain points.

Analyses:
130: blobtools fig 3 - How were the mammalian host reads separated? The detailed methods [Lines 869-874] don't specify any GC/Cov cutoffs. If you just go by the blast hits in the blobtools report, you're likely to miss a lot of mammalian host contigs. The blobtools figure is also very incomplete - it doesn't have the marginal histograms which help assess coverage/levels of contamination.

>Reads mapped on the contigs with node coverage <3, but with the contigs annotated as Arthropoda, were kept; while all of the contigs annotated as Chordata were removed (see new text; p. 41, lines 969 -971 A new version of Figure 3 with marginal histograms has been created.

Also - is there a blobplot for Dt? Lines 857-868 don't describe it. That's the one that needs it. Please do the taxonomic annotation at the level of order rather than superkingdom as the contamination could be from other eukaryotes too.

>We have now included blobplots for Dt with taxonomic annotation at superkingdom level (supplemental fig. S11). Restricting the final assembly to scaffolds of >1,000 bp eliminated a high-GC “Eukaryota” blob that we assume represents gut contents (compare panels of fig. S11). Increasing taxonomic resolution to order level and re-plotting the data using MegaBLAST was not informative, as most of the contigs were not annotated (see “Figure A for Review”). This appears to be due to the lack of closely related mite sequences in the databases.

157: Please provide numbers in text. Numbers are very low esp as arthropoda should be complete. Addnl File 1 - Dt BUSCO Duplicated REALLY high compared to others. Try BUSCO Metazoa/Eukaryota?

>The BUSCO Arthropoda gene set appears to work well with the acarine long-read assemblies that have been published while our manuscript has been in revision, including for another acariform mite (P. ovis; see above). Thus, our lower BUSCO scores are due to the use of short-read technology but hold up very well against the closest thing we have to an acariform reference, which is the T. urticae genome. That genome is considerably more contiguous than our trombidid assemblies and has a much lower repeat content, but its complete BUSCO score (69%) is equivalent to those for our assemblies.

We concede that the BUSCO duplicated gene rate in the D. tinctorium assembly is very high and deserves further exploration. Therefore, we ran a Redundans analysis on the scaffolds generated by our Abyss assembly. This reduced the number of scaffolds from 22,761 to 12,493 and the assembly size from 180 Mb to 114 Mb. Gene content was reduced dramatically, from 19,258 to 12,322. However, the BUSCO score for complete genes was unaffected (~72%) and the proportion of duplicated genes was reduced from 34% to 12% (see new supplemental table S3).

While on first glance, Redundans appears to have helped resolve a problem caused by high levels of heterozygosity, on closer inspection we believe the situation is more complex. The GenomeScope analysis for Dt (supplemental Fig. S1b) indicated very low levels of heterozygosity, and we validated this by comparing the gene sequences of the duplicates, which are 100% identical at the nucleotide level. However, while the scaffolds on which the duplicates are located are often very similar, they exhibit small deletions and SNPs outside the boundaries of the genes (see new supplemental table S4).

It is important to emphasize that the Dt assembly is the only genome obtained from a single mite to date; those published for other species typically used a pool containing thousands of individuals. Yet, high rates of duplication were not observed in these other mite genome assemblies. Unlike many other mite species, Dt cannot be readily sexed by external characteristics and it is possible that we sequenced a female containing a spermatophore or fertilised eggs (or even gut contents containing other individuals, as cannibalism is common in this taxon). However, in those scenarios, we would expect some allelic variation between the “duplicated” genes.

While only a long-read approach could rule-out possible assembly errors conclusively, plausible biological explanations for the data include recent large-scale ectopic recombination of a chromosome segment, aneuploidy, or the presence of a B chromosome. This would be compatible with the higher repeat content in this genome compared with that for other acariform mites, which might facilitate large-scale duplications. Notably, whole genome duplication has been reported in other chelicerates, although this is thought to be ancient in the spider-scorpion lineage (see DOI 10.1186/s12915-017-0399-x).

Therefore, as we believe the Redundans analysis to be overly conservative in this specific case, we have retained the original Abyss assembly and downstream analyses based on this. We also judge that the CAFE gene expansion analysis is reliable, as the program was designed to work on fragmented genomes (and our genomes show a similar or lower level of gene fragmentation by BUSCO compared with other arachnid genomes; i.e., ~10%). Moreover, several families of expanding genes (e.g., the terpene synthases – a major focus of the manuscript – and some of the retroviral genes) are shared between Dt and Ld, even though the latter displays very low levels of duplication. This indicates that even if the high duplication level in Dt is an artefact, the comparisons between the two genomes appear to be robust and make biological sense.

New text has been added to the Results (p. 9, lines 168 – 181) and Discussion (p. 28, lines 637 – 659) for the Redundans analysis and its interpretation.

159: Comparing favourably with other arachnid genomes could be because of poor arachnid genomes?

>See response above to previous query.

160: Greatest num of genes could be an artefact of gene prediction. Fig 4 is an excellent representation - I wish more papers would use such a representation. Addnl File 2 is helpful but these kmer plots indicate a very hard to deal with sequencing project - the error peak is too close to the main peak. I know of labs where this kmer plot would be used for quality control and the sequencing would be redone to get higher coverage, perhaps even from re-extracted DNA. Is there a good reason why this was not done in this case?

>Regarding the point about the number of genes in Dt, please see the response to the duplication issue above. We agree that the k-mer plots indicate a deficiency in sequencing depth for Ld, but a significant increase in coverage would have required more material, and this was simply not available (see points raised under the editor’s comments).

Addnl File 3 What does "unclassified" mean in this table?

>Unclassified repeats are simply those that cannot be assigned to known categories.

170: Did you try OrthoFinder? OrthoMCL is venerable but outdated and has known problems with predicting orthology/homology at larger distances. OrthoFinder accounts for greater phylogenetic distance by normalising pairwise scores for each pair of species. I realise that redoing the orthology analyses would change EVERY gene family result/figure/table in the manuscript, which is a big ask, but I would appreciate if you could run OrthoFinder just to double check that the results aren't dramatically different. If they are roughly the same (eg: number of 1:1, patchy 1:1, singletons, etc) then go ahead with the existing OrthoMCL analyses, but if they change dramatically, then it would imply that the gene-family findings aren't robust and are highly sensitive to the methods used, and should perhaps be re-examined.

>We investigated this comparison between OrthoMCL and OrthoFinder in some depth. The former found more 1:1:1 and patchy orthologues, as well as species-specific genes, compared with the latter (see “Table A for review”). On the other hand, OrthoFinder found more N:N:N orthologues than OrthoMCL. The main disadvantage of OrthoMCL that OrthoFinder was designed to correct is length bias in BLAST scores (longer sequences produce higher bit scores and lower e-values than short sequences). We compared the length distribution of the 1:1:1 orthologues identified by OrthoFinder or OrthoMCL. Although the OrthoMCL-specific group included a more pronounced positive skew of longer (>1,000 aa) sequences compared with OrthoFinder, the median length was very similar between the two programs (see “Figure B for review”). In fact, the median length of the OrthoFinder gene clusters was slightly greater than those that were identified by OrthoMCL.

We assessed the impact of using OrthoFinder on the two main conclusions of the manuscript as highlighted in the manuscript title: lateral gene transfers and specific allergens. In the CAFE analysis of expanding/contracting gene families, the terpene synthase genes (putative lateral gene transfers) were identified as significantly expanded by OrthoFinder in agreement with OrthoMCL. However, for the predicted allergens, OrthoFinder placed most Ld and Dt clusters in a common core shared with the other four mite species included in the analysis (see “Figure C for review”). In contrast to OrthoMCL, no species-specific clusters were identified in Ld or Dt.

To determine if the specific allergen clusters originally identified by OrthoMCL were phylogenetically distinct, we generated a tree for all predicted allergen sequences. In the vast majority of cases, the species-specific OrthoMCL clusters were recovered as distinct branches on the tree (see “Figure D for review”), indicating that OrthoFinder could be considered “overzealous” in its clustering in this context.

Our conclusion from this exercise is that while different clustering programs will generate different results, it is important to assess the appropriateness of the programs in the context of the specific study. In our study, OrthoMCL clearly provided finer-resolution clustering among acarine species than did OrthoFinder, and these data are more biologically plausible than the OrthoFinder output, especially for the allergens.

172: "substantially greater number of unique paralogous groups" could be an indicator of poor assembly quality with uncollapsed haplotypes. This finding would also be consistent with the BUSCO duplicates percentage.

>Addressed above.

183: Could this be a viral contaminant?

>This is a misunderstanding: FLVCR domain is related to the mammalian receptor for feline leukemia virus subgroup C, so is not a viral protein.

195: dN/dS = 1.06 is quite close to 1 and I don't think this implies a case for positive selection.

>We have removed all reference to this gene from the text.

Overrepresented protein families detected by LC-MS/MS: I can't comment on this section as I have no expertise, but it reads clearly

233-274 - Agree. The terpene synthases seem to come from bacteria

275-290 - Agree. Bacterial contamination seems unlikely.

291-300 - If we can't say for sure where these sequences came from (see blobplot comments as well) then perhaps it is too early to claim that trombidid genomes exhibit ERV dynamism?

>Only the Ld material was significantly contaminated by host material (adult Dt are free-living), but ERV gene expansions were seen in both genomes. Moreover, in addition to the Gag polyprotein-like genes of potential mammalian origin in Ld (which we concede might come from host contamination), other significant ERV gene expansions (Pol polyprotein-like and integrases) was identified in this genome when CAFE was run again following incorporation of the horseshoe crab data (see response to referee 1 above). Unlike the Gag proteins, the closest homologues of these Pol polyproteins and integrases are found in other arthropods. Trees for these viral proteins from Ld are now included in supplemental fig. S7 and S8. Minor changes have been made to the Results text to reflect this (page 16, line 317 – 344).

Immune System, Photoreceptor and chemosensory systems, Predicted allergens, Putative salivary proteins - I have no expertise in these gene families and cannot comment.

Discussion:

570-571: Agree that the data are useful. Although I feel unqualified to comment on the gene family analyses, a quick skim of the discussion section suggested that it was quite long and often reiterated the results/analyses section. Perhaps the main findings should be summarised in one place rather than both?

>Due to the impact of new molecular clock estimates that include the horseshoe crab genome data and constrained dates for evolutionary splits between key phyla (see response to referee 1), a small number of changes in the CAFE analysis were observed. For instance, the 4-coumarate--CoA ligase and dsRNA-activated kinase genes are no longer detected to be expanding significantly, so some corresponding text from the Results and Discussion has been cut (see deleted paragraphs on pages 11, 19, 33 and 35). We have now also tried to cover the basic genome statistics and sequencing limitations in the Results only, using the Discussion to raise the broader question of chelicerate genome quality more generally. The other gene-family analysis sections follow a consistent style in which some limited but essential context is provided in the Results; whereas in the Discussion, the biological implications are considered in more depth.

Detailed Methods:

852: Kraken - individual reads could be HGT. So calling it contamination seems harsh. What kraken database was chosen?

>We used the standard (Mini-Kraken) database (clarified in the Methods) and now refer to “reads of bacterial origin” rather than contamination (p. 40, lines 942 - 944).

857-868: No blobplot for Dt genome?

>This has now been included as supplemental figure S11

950: We used a modification of the Crisp method - what was the modification?

>The class A criterion and that no gene in the orthologous group had a best metazoan bit-score of <100 was not applied, as it was considered too strict for large gene clusters. This has been clarified in the text (p. 45, lines 1062 – 1064).

1187: Figure 8 Legend should say blob plot of contigs containing genes. With contigs containing terpene synthase genes in red and all other contigs containing other genes in blue.

>The legend is correct: genes rather than contigs are shown in this particular case.

We hope that following the comprehensive QC we have performed on the genome analyses and the corresponding thorough revision of the manuscript, it will now be found suitable for publication in GigaScience.

Yours sincerely,
Ben Makepeace

Close
