# Supplementary material for: Genomes of trombidid mites reveal novel predicted allergens and laterally transferred genes associated with secondary metabolism
Source: Gigascience. 2018 Nov 15;7(12):giy127. doi: 10.1093/gigascience/giy127 (PMC6275457; doi:10.1093/gigascience/giy127)
Supplement: Response_to_Reviewer_Comments_Revision_1.pdf [file giy127_response_to_reviewer_comments_revision_1.pdf]

Author's Response To Reviewer Comments

Close

Dear Dr Zauner,
Thank you very much for indicating a provisional acceptance of our manuscript, subject to incorporating some additional discussion pertaining to referee 1's comments below.
"Reviewer #1: The authors addressed the majority of the issues raised by the reviewers in a satisfactory manner. I have to revisit the issue of the molecular clock analysis since this would still impact on the paper in a significant manner.
Molecular clock: Based on the current analysis spiders and horseshoe crabs diverged 336 MYA (263-394). However, the oldest horseshoe crab fossils are dated at 445 MYA, even if crown Xiphosura has younger minimum age estimates. This would imply that the current tree presented are not dated correctly and that age estimates across the Arachnida would be much older (the split between spiders and horseshoe crabs at least 445 MYA in the current tree topology). I suspect that the phylogenetic placement of the Xiphosura in the current tree is probably artefactual and contribute towards the problems in dating their tree correctly.
The comments made by the authors regarding uncertainty in arachnid systematics is acknowledged. However, these analyses have very specific and important implications for interpretation of their data, as well as its technical correctness."
Our response:
We think that the referee's original suggestion of incorporating the Limulus genome into our phylogenomic analysis was raised in part to address dating issues within our tree, as the fossil record for xiphosurans is quite extensive. In revising the tree, we paid attention to the referee's advice considering constrained nodes [Ecdysozoa (Nematode-Arthropod split) recently calibrated at 528.8-636 MYA and the split between Chelicerata and Mandibulata constrained at 514-636 MYA]. These constraints had a modest effect (~10 million years) on the dating of the radiation with the Trombidiformes, which did not materially affect our conclusions. While we agree that the placement of Limulus appears erroneous, it probably has a relatively straightforward explanation (a major difference in evolutionary rates between xiphosurans and mites), which other authors have raised previously (PMID: 25107551).
In our view, following review of the literature on the arachnid fossil record (PMID: 20093195) and earlier molecular phylogenetic studies using a few gene loci (PMID: 20060051), the dates of real interest to our study (branches within the Trombidiformes) remain highly plausible. The key point is that the Trombiculoidea are not a Paleogene novelty as had been purported previously. It is possible that resolving the problems around the placement of the Xiphosura will push back the key periods of diversification within the Trombidiformes to an even earlier period, but we have presented the more conservative interpretation at this point until additional data clarifies the key dates in the origin of the major chelicerate groups.
Text in the Discussion has been revised accordingly with additional references as follows (see also manuscript file with highlights).
"…This can lead to paraphyletic models of arachnid evolution, including the nested placement of the horseshoe crabs (order Xiphosura) within the Arachnida as we observed here, and a node age for divergence from the Araneae (263.2 – 394.7 MYA, Fig. 4) that appears much too recent compared with the oldest xiphosuran fossil, which has been dated to the late Ordovician [144]. However, other key nodes, such as the divergence between the Acariformes and the (Araneae + Parasitiformes), as well as the Araneae-Parasitiformes split, were dated within the range of previous estimates based on the fossil record [145], or more

recently, nuclear genomes [64]. While the controversial phylogeny of the Chelicerata at higher taxonomic levels is beyond the scope of our study, at finer scales, the phylogenomic analyses supported the conventional morphology-based taxonomy within the Acariformes, confirming that the trombidid mites are closely related to the phytophagous Tetranychoidea [15]. Moreover, based on an earlier analysis using nuclear and mitochondrial markers [146], our dating of the split between the Tetranychoidea and the Trombidiae is consistent with a secondary radiation within the Trombidiformes that is hypothesised to have occurred during the Carboniferous."

Yours sincerely,

Ben Makepeace

Close
