# Supplementary material for: Genomes of trombidid mites reveal novel predicted allergens and laterally transferred genes associated with secondary metabolism
Source: Gigascience. 2018 Nov 15;7(12):giy127. doi: 10.1093/gigascience/giy127 (PMC6275457; doi:10.1093/gigascience/giy127)
Supplement: Response_to_Reviewer_Comments_Revision_2.pdf [file giy127_response_to_reviewer_comments_revision_2.pdf]

Author's Response To Reviewer Comments

Close

Dear Dr Zauner,

Following our e-mail discussion, I have removed the "data not shown" statement pertaining to the Bayesian tree method. I have also uploaded new figures at higher resolution.

With thanks for your assistance,
Ben Makepeace

Close
