# Supplementary material for: Genomes of trombidid mites reveal novel predicted allergens and laterally transferred genes associated with secondary metabolism
Source: Gigascience. 2018 Nov 15;7(12):giy127. doi: 10.1093/gigascience/giy127 (PMC6275457; doi:10.1093/gigascience/giy127)
Supplement: Reviewer_1_Report_(Original_Submission) -- Ben Mans — 05 Mar 2018 Reviewed [file giy127_reviewer_1_report_(original_submission).pdf]

Reviewer Report

Title: Genomes of trombidid mites reveal novel predicted allergens and laterally-transferred genes associated with secondary metabolism

Version: Original Submission **Date:** 3/5/2018

Reviewer name: Ben Mans

Reviewer Comments to Author:

The authors report the genomes of two important trombidid mites, namely the giant red velvet mite, Dinothrombidium tinctorium, the world's largest acarid species and the chigger, Leptotrombidium delicense, the primary scrub typhus vector in South-East Asia. This brings the number of Acariform genomes to four and allows important insights into the biology and evolution of the Acari. Specific horizontal gene transfer of terpene and carotenoid synthesis genes from soil-derived bacteria and fungi are illuminated as unique features of trombidid mites. A variety of protein families involved in sensing were analyzed including gustatory, olfactory and ioinotropic receptors, odorant binding and chemosensory proteins and opsins. The results support earlier observations that olfactory receptors and odorant binding proteins are absent in the Arachnida. Potential new allergens were predicted and salivary orthologs shared among Acari identified. Proteomic analysis of whole body extracts from adult red velvet mites and larval chiggers also identified an extensive number of proteins encoded in the genome.

The sequencing and genome assembly follows well validated methods and BUSCO analysis indicate that completeness of the genomes are similar to other published acarid genomes to date. Similarly, the proteomics methods may be considered part of standard workflows and the results obtained should be seen in the light of the complexity of whole organism extracts. The statistics reported and employed are appropriate in all analyses. Overall, the study is comparable to others of its kind. The study adds an extensive amount of new data that makes this an important contribution within its field. It is well written, the methodology followed clear and understandable. Most of the conclusions are warranted and the manuscript was instructive and enjoyable to read. I have no doubt that this study is worthy of publication. Some issues follow below that should be considered.

Page 9, lines 162-169: The author's presents an interesting divergence date analysis (Fig. 4). The exclusion of the horse-shoe crab from the analysis, for which genomes have been sequenced is noteworthy and could affect some of the estimated divergence dates. Other nodes that may need to be considered is that of the crown Ecdysozoa (Nematode-Arthopod split) that was recently calibrated at 528.8-636 MYA (Benton et al. 2015). The split between Chelicerata and Mandibulata should also be considered to be constrained at 514-636 MYA (Benton et al. 2015). This would make all molecular clock estimates slightly older than in the current analysis. It would also affect some of the conclusions made in the discussion (Page 26, line 588).
Page 9, line 158; Page 37, line 867: The impressive CEGMA score may be amended to also include the BUSCO scores that is not so impressive and does indicate that the genomes are not as complete as suggested. While the completeness of the genomes compare well with other genomes sequenced to date, it may be good to indicate that for the majority of acarid genomes we still miss ~15-20% of predicted gene data. It should also be underscored that CEGMA/BUSCO gives an indication of housekeeping genes and that numbers for lineage specific expanded genes (gene duplicates) may be under represented.
Page 11, lines 201-231: It may be indicated that organ specific proteins (salivary proteins) may be underrepresented in the whole body proteomes, even if they might be highly abundant and present at concentrations that would make them important allergens or host modulatory molecules.
Page 14, lines 279-282: Could the authors also state whether the average coverage for the terpene synthase was the same as the contig it was found in and whether this hold for the other terpene synthases as well. This would also support it as a bona fide LGT. On this, the authors do not explicitly mention this, but the phylogenetic trees suggest lineage specific expansions (gene duplication) of this family in these mites.

Does this mean that the genes are scattered through the genome or are they found on limited scaffolds/chromosomes?
Page 16, line 333-346: A third explanation for absence of the IMD pathway in acariform mites is that the Acari are paraphyletic and that immune deficiency pathways evolved in independent lineages after divergence of the chelicerata.
Page 22-25, line 487-564: The study by Kim et al. (2016) described proteins found in tick salivary secretions. Of these, ~50% could be considered house-keeping with the caveat that ticks may secrete non-secretory proteins by apocrine secretion. Their functionality in feeding still needs to be demonstrated. The majority of orthologs found in Table 3 all seem to resemble house-keeping proteins and singletons. Given the absence of secretory protein families, this approach seems tenuous to identify salivary proteins from chiggers. What these results seem to suggest is that chiggers have a different salivary repertoire compared to ticks. It may be fruitful to look at highly expanded secretory families such as those reported in additional file 6.

Page 47, line 1110: It was not clear that the genome assembly and related data has been submitted to the SRA database or Genbank public repositories, since no accession numbers will be associated with these sequences, nor will future BLAST analysis against the public databases be possible.

Minor
Page 34, lines 790-798: The date for the collection of the chiggers is indicated, but not for the red velvet mites.
Page 38: line 882: Three ab initio…
Page 39: line 920: 926 orthologous are indicated, while the phylogenetic tree (Figure 1) states 527. No indication in text as to what happened to the 399 orthologs not included in the tree.
Page 42, line 981: …chemosensory proteins in….
Page 44, line 1042: …prepared by homogenisation…

Methods

Are the methods appropriate to the aims of the study, are they well described, and are necessary controls included? Yes

Conclusions

Are the conclusions adequately supported by the data shown? Yes

Reporting Standards

Does the manuscript adhere to the journal's guidelines on minimum standards of reporting? Yes

Choose an item.

Statistics

Are you able to assess all statistics in the manuscript, including the appropriateness of statistical tests used? Yes, and I have assessed the statistics in my report.

Quality of Written English

Please indicate the quality of language in the manuscript: Acceptable

Declaration of Competing Interests

Please complete a declaration of competing interests, considering the following questions:

- Have you in the past five years received reimbursements, fees, funding, or salary from an organisation that may in any way gain or lose financially from the publication of this manuscript, either now or in the future?
- Do you hold any stocks or shares in an organisation that may in any way gain or lose financially from the publication of this manuscript, either now or in the future?
- Do you hold or are you currently applying for any patents relating to the content of the manuscript?
- Have you received reimbursements, fees, funding, or salary from an organization that holds or has applied for patents relating to the content of the manuscript?
- Do you have any other financial competing interests?
- Do you have any non-financial competing interests in relation to this paper?

If you can answer no to all of the above, write 'I declare that I have no competing interests' below. If your reply is yes to any, please give details below.

I declare that I have no competing interests

I agree to the open peer review policy of the journal. I understand that my name will be included on my report to the authors and, if the manuscript is accepted for publication, my named report including any attachments I upload will be posted on the website along with the authors' responses. I agree for my report to be made available under an Open Access Creative Commons CC-BY license (http://creativecommons.org/licenses/by/4.0/). I understand that any comments which I do not wish to be included in my named report can be included as confidential comments to the editors, which will not be published.

I agree to the open peer review policy of the journal

To further support our reviewers, we have joined with Publons, where you can gain additional credit to further highlight your hard work (see: https://publons.com/journal/530/gigascience). On publication of this paper, your review will be automatically added to Publons, you can then choose whether or not to claim your Publons credit. I understand this statement.

Yes Choose an item.
