# Supplementary material for: Genomes of trombidid mites reveal novel predicted allergens and laterally transferred genes associated with secondary metabolism
Source: Gigascience. 2018 Nov 15;7(12):giy127. doi: 10.1093/gigascience/giy127 (PMC6275457; doi:10.1093/gigascience/giy127)
Supplement: Reviewer_1_Report_Revision_1 -- Ben Mans — 8/14/2018 Reviewed [file giy127_reviewer_1_report_revision_1.pdf]

Reviewer Report

Title: Genomes of trombidid mites reveal novel predicted allergens and laterally-transferred genes associated with secondary metabolism

Version: Revision 1 **Date:** 8/14/2018

Reviewer name: Ben Mans

Reviewer Comments to Author:

The authors addressed the majority of the issues raised by the reviewers in a satisfactory manner. I have to revisit the issue of the molecular clock analysis since this would still impact on the paper in a significant manner.

Molecular clock: Based on the current analysis spiders and horseshoe crabs diverged 336 MYA (263-394). However, the oldest horseshoe crab fossils are dated at 445 MYA, even if crown Xiphosura has younger minimum age estimates. This would imply that the current tree presented are not dated correctly and that age estimates across the Arachnida would be much older (the split between spiders and horseshoe crabs at least 445 MYA in the current tree topology). I suspect that the phylogenetic placement of the Xiphosura in the current tree is probably artefactual and contribute towards the problems in dating their tree correctly. The comments made by the authors regarding uncertainty in arachnid systematics is acknowledged. However, these analyses have very specific and important implications for interpretation of their data, as well as its technical correctness.

Methods

Are the methods appropriate to the aims of the study, are they well described, and are necessary controls included? Yes

Conclusions

Are the conclusions adequately supported by the data shown? Yes

Reporting Standards

Does the manuscript adhere to the journal's guidelines on minimum standards of reporting? Yes

Choose an item.

Statistics

Are you able to assess all statistics in the manuscript, including the appropriateness of statistical tests used? Yes, and I have assessed the statistics in my report.

Quality of Written English

Please indicate the quality of language in the manuscript: Acceptable

Declaration of Competing Interests

Please complete a declaration of competing interests, considering the following questions:

- Have you in the past five years received reimbursements, fees, funding, or salary from an organisation that may in any way gain or lose financially from the publication of this manuscript, either now or in the future?
- Do you hold any stocks or shares in an organisation that may in any way gain or lose financially from the publication of this manuscript, either now or in the future?
- Do you hold or are you currently applying for any patents relating to the content of the manuscript?
- Have you received reimbursements, fees, funding, or salary from an organization that holds or has applied for patents relating to the content of the manuscript?
- Do you have any other financial competing interests?
- Do you have any non-financial competing interests in relation to this paper?

If you can answer no to all of the above, write 'I declare that I have no competing interests' below. If your reply is yes to any, please give details below.

The corresponding author and myself are part of a recently formed consortium focusing on the genome sequencing of "1000 Ixodes" genomes. I do not have other professional or personal association with him beyond reviewing a paper of the same group that was also published in GigaScience.

I agree to the open peer review policy of the journal. I understand that my name will be included on my report to the authors and, if the manuscript is accepted for publication, my named report including any attachments I upload will be posted on the website along with the authors' responses. I agree for my report to be made available under an Open Access Creative Commons CC-BY license (http://creativecommons.org/licenses/by/4.0/). I understand that any comments which I do not wish to be included in my named report can be included as confidential comments to the editors, which will not be published.

I agree to the open peer review policy of the journal

To further support our reviewers, we have joined with Publons, where you can gain additional credit to further highlight your hard work (see: https://publons.com/journal/530/gigascience). On publication of this paper, your review will be automatically added to Publons, you can then choose whether or not to claim your Publons credit. I understand this statement.

Yes Choose an item.
