# Supplementary material for: Genomes of trombidid mites reveal novel predicted allergens and laterally transferred genes associated with secondary metabolism
Source: Gigascience. 2018 Nov 15;7(12):giy127. doi: 10.1093/gigascience/giy127 (PMC6275457; doi:10.1093/gigascience/giy127)
Supplement: Reviewer_2_Report_(Original_Submission) -- Sujai Kumar — 4/23/2018 Reviewed [file giy127_reviewer_2_report_(original_submission).pdf]

Reviewer Report

Title: Genomes of trombidid mites reveal novel predicted allergens and laterally-transferred genes associated with secondary metabolism

Version: Original Submission **Date:** 4/23/2018

Reviewer name: Sujai Kumar

Reviewer Comments to Author:

The authors report two draft genomes (one with quite low coverage) of trombidid mites, and provide a comprehensive analysis of the gene families that they found in these two species. At a time of long range sequencing with nanopore/pacbio/dovetail/hi-C, some might argue that these aren't great assemblies. However, I firmly believe that even lower quality draft genomes should be made available and analyses published as long as the limitations of the resources / analyses are clearly spelled out.

My biggest concern is that although some very comprehensive gene-family analyses have been presented in this manuscript, the genomes are of such low qualities that some of the findings may be artefacts. It is possible that it is very hard to get more DNA material from these species and therefore this is the best genomic resource we are going to have for a while. However, if the species can be resequenced to higher depths (thus mitigating the poor kmer plots in Addnl file 3), then the manuscript would be much more valuable. Either way, I'd like to see the issue of a poor genome assembly addressed more explicitly in the text, and all analyses put in that context.

Manuscript presentation comments:

This is more of a formatting request but one that will hopefully make life easier for the readers of the final paper: please consider consolidating all supplemental tables into one excel (or other spreadsheet software) file with separate tables; please put all supplemental figures into one pdf rather than a separate pdf for each figure. Some of the tables are also submitted as additional PDF files (eg Additional Files 3, 20) when they could clearly be an extra separate supplemental tables inside a single excel file.

Several figures in the main manuscript could also be consolidated into single figures. For example, all the phylogenetic trees could be subparts of one figure, which would make it easier to compare the findings of different analyses.

Please also consider sending the resubmission and future manuscripts with inline figures and tables (like a thesis). Modern production systems can easily deal with such manuscripts. It doubles the effort for both the writers and reviewers if there are separate documents for text, figures, tables, legends.

Background:

Excellently written - thank you. I learnt lots about why these species / clades are important. A general note: I freely admit I'm no systematist, and I don't know what the convention is, but I would have guessed taxon names should be considered as plural nouns unless prefixed with the rank. Eg, "Most Nematoda are ...", but "The phylum Nematoda is ..." sound correct to me. Am happy to be told I'm wrong if that's not the convention, but this manuscript needs to be consistent. Some lines treat taxa as singular "The Acari is ...", "The Parasitiformes harbours ...", whereas others treat taxon names as plural "of the Trombidiformes in response to their ...".

In the data description, I suggest using the scientific names or acronyms (Dt / Ld) rather than switching back and forth between Dt and velvet mite, and Ld and chigger.

Analyses:

130: blobtools fig 3 - How were the mammalian host reads separated? The detailed methods [Lines 869-874] don't specify any GC/Cov cutoffs. If you just go by the blast hits in the blobtools report, you're likely to miss a lot of mammalian host contigs. The blobtools figure is also very incomplete - it doesn't have the marginal histograms which help assess coverage/levels of contamination.

Also - is there a blobplot for Dt? Lines 857-868 don't describe it. That's the one that needs it.
Please do the taxonomic annotation at the level of order rather than superkingdom as the contamination could be from other eukaryotes too.

157: Please provide numbers in text. Numbers are very low esp as arthropoda should be complete.
Addnl File 1 - Dt BUSCO Duplicated REALLY high compared to others. Try BUSCO Metazoa/Eukaryota?
159: Comparing favourably with other arachnid genomes could be because of poor arachnid genomes?
160: Greatest num of genes could be an artefact of gene prediction.
Fig 4 is an excellent representation - I wish more papers would use such a representation.
Addnl File 2 is helpful but these kmer plots indicate a very hard to deal with sequencing project - the error peak is too close to the mmain peak. I know of labs where this kmer plot would be used for quality control and the sequencing would be redone to get higher coverage, perhaps even from re-extracted DNA. Is there a good reason why this was not done in this case?

Addnl File 3 What does "unclassified" mean in this table?

170: Did you try OrthoFinder? OrthoMCL is venerable but outdated and has known problems with predicting orthology/homology at larger distances. OrthoFinder accounts for greater phylogenetic distance by normalising pairwise scores for each pair of species. I realise that redoing the orthology analyses would change EVERY gene family result/figure/table in the manuscript, which is a big ask, but I would appreciate if you could run OrthoFinder just to double check that the results aren't dramatically different. If they are roughly the same (eg: number of 1:1, patchy 1:1, singletons, etc) then go ahead with the existing OrthoMCL analyses, but if they change dramatically, then it would imply that the gene-family findings aren't robust and are highly sensitive to the methods used, and should perhaps be re-examined.

172: "substantially greater number of unique paralogous groups" could be an indicator of poor assembly quality with uncollapsed haplotypes. This finding would also be consistent with the BUSCO duplicates percentage.

183: Could this be a viral contaminant?
195: dN/dS = 1.06 is quite close to 1 and I don't think this implies a case for positive selection.

Overrepresented protein families detected by LC-MS/MS: I can't comment on this section as I have no expertise, but it reads clearly

233-274 - Agree. The terpene synthases seem to come from bacteria

275-290 - Agree. Bacterial contamination seems unlikely.

291-300 - If we can't say for sure where these sequences came from (see blobplot comments as well) then perhaps it is too early to claim that trombidid genomes exhibit ERV dynamism?

Immune System, Photoreceptor and chemosensory systems, Predicted allergens, Putative salivary proteins - I have no expertise in these gene families and cannot comment.

Discussion:

570-571: Agree that the data are useful.
Although I feel unqualified to comment on the gene family analyses, a quick skim of the discussion section suggested that it was quite long and often reiterated the results/analyses section. Perhaps the main findings should be summarised in one place rather than both?

Detailed Methods:

852: Kraken - individual reads could be HGT. So calling it contamination seems harsh. What kraken database was chosen?

857-868: No blobplot for Dt genome?

950: We used a modification of the Crisp method - what was the modification?

1187: Figure 8 Legend should say blob plot of contigs containing genes. With contigs containing terpene synthase genes in red and all other contigs containing other genes in blue.

Methods

Are the methods appropriate to the aims of the study, are they well described, and are necessary controls included? Choose an item.

Conclusions

Are the conclusions adequately supported by the data shown? Choose an item.

Reporting Standards

Does the manuscript adhere to the journal's guidelines on minimum standards of reporting? Choose an item.

Choose an item.

Statistics

Are you able to assess all statistics in the manuscript, including the appropriateness of statistical tests used? Yes, and I have assessed the statistics in my report.

Quality of Written English

Please indicate the quality of language in the manuscript: Choose an item.

Declaration of Competing Interests

Please complete a declaration of competing interests, considering the following questions:

- Have you in the past five years received reimbursements, fees, funding, or salary from an organisation that may in any way gain or lose financially from the publication of this manuscript, either now or in the future?

- Do you hold any stocks or shares in an organisation that may in any way gain or lose financially from the publication of this manuscript, either now or in the future?
- Do you hold or are you currently applying for any patents relating to the content of the manuscript?
- Have you received reimbursements, fees, funding, or salary from an organization that holds or has applied for patents relating to the content of the manuscript?
- Do you have any other financial competing interests?
- Do you have any non-financial competing interests in relation to this paper?

If you can answer no to all of the above, write 'I declare that I have no competing interests' below. If your reply is yes to any, please give details below.

I declare that I have no competing interests

I agree to the open peer review policy of the journal. I understand that my name will be included on my report to the authors and, if the manuscript is accepted for publication, my named report including any attachments I upload will be posted on the website along with the authors' responses. I agree for my report to be made available under an Open Access Creative Commons CC-BY license (http://creativecommons.org/licenses/by/4.0/). I understand that any comments which I do not wish to be included in my named report can be included as confidential comments to the editors, which will not be published.

I agree to the open peer review policy of the journal

To further support our reviewers, we have joined with Publons, where you can gain additional credit to further highlight your hard work (see: https://publons.com/journal/530/gigascience). On publication of this paper, your review will be automatically added to Publons, you can then choose whether or not to claim your Publons credit. I understand this statement.

Yes Choose an item.
